
JOURNAL INFORMATION
==============================
NLM Title Abbreviation: Sci Pharm
Journal ID: Sci Pharm
NLM Journal ID: 0026251
Journal ID: Scientia Pharmaceutica

Scientia Pharmaceutica

ISSN: 0036-8709
EISSN: 2218-0532
Publisher: Österreichische Apotheker-Verlagsgesellschaft, m. b. H.

ARTICLE INFORMATION
==============================
PMCID: PMC3002819
Article ID: Scipharm.2010.78.590
Article version: 1
Subjects: Abstracts of the 8th Central European Symposium on Pharmaceutical Technology (CESPT), Satellite Symposium: 4th International Graz Congress on Pharmaceutical Engineering, September 16th-18th, Graz, Austria

Posters (PDD, PMS, PNM, PPAT, POT)

Electronic publication date: 2010 Sep 30
Print publication date: 2010 Jul-Sep
Volume: 78
Issue: 3
First page: 590
Last page: 727
Copyright: © author(s); licensee Österreichische Apotheker-Verlagsgesellschaft m. b. H., Vienna, Austria.
Copyright year: 2010
License: This is an Open Access article distributed under the terms of the Creative Commons Attribution License (http://creativecommons.org/licenses/by/3.0/), which permits unrestricted use, distribution, and reproduction in any medium, provided the original work is properly cited.
License URL: https://creativecommons.org/licenses/by/3.0/

==============================
JOURNAL INFORMATION
==============================
NLM Title Abbreviation: Sci Pharm
Journal ID: Sci Pharm
NLM Journal ID: 0026251
Journal ID: Scientia Pharmaceutica

Scientia Pharmaceutica

ISSN: 0036-8709
EISSN: 2218-0532
Publisher: Österreichische Apotheker-Verlagsgesellschaft, m. b. H.

ARTICLE INFORMATION
==============================
DOI: 10.3797/scipharm.cespt.8.PDD01
Article ID: scipharm.2010.78.590a
Subjects: Conference abstract PDD01

Interaction of β-Cyclodextrin with DNA-Bases

Liedl E. 1
Beyer A. 1
Wolschann P. 1
Viernstein H. 2
1 Institute of Theoretical Chemistry, University of Vienna, 1090 Vienna, Austria
2 Department of Pharmaceutical Technology and Biopharmaceutics, University of Vienna, 1090 Vienna, Austria
E-mail: helmut.viernstein@univie.ac.at (H. Viernstein)
Electronic publication date: 2010 Sep 30
Print publication date: 2010 Jul-Sep
Volume: 78
Issue: 3
First page: 590
Last page: 590
Copyright: © author(s); licensee Österreichische Apotheker-Verlagsgesellschaft m. b. H., Vienna, Austria.
Copyright year: 2010
License: This is an Open Access article distributed under the terms of the Creative Commons Attribution License (http://creativecommons.org/licenses/by/3.0/), which permits unrestricted use, distribution, and reproduction in any medium, provided the original work is properly cited.

JOURNAL INFORMATION
==============================
NLM Title Abbreviation: Sci Pharm
Journal ID: Sci Pharm
NLM Journal ID: 0026251
Journal ID: Scientia Pharmaceutica

Scientia Pharmaceutica

ISSN: 0036-8709
EISSN: 2218-0532
Publisher: Österreichische Apotheker-Verlagsgesellschaft, m. b. H.

ARTICLE INFORMATION
==============================
DOI: 10.3797/scipharm.cespt.8.PDD02
Article ID: scipharm.2010.78.591
Subjects: Conference abstract PDD02

Quantifying nuclear import of lipoplex-delivered-pDNA in A10 and MDCK cells

Steinbach A.
Süss R.
Department of Pharmaceutical Technology and Biopharmacy, University of Freiburg, Freiburg, Germany
E-mail: annette.steinbach@pharmazie.uni-freiburg.de (A. Steinbach)
Electronic publication date: 2010 Sep 30
Print publication date: 2010 Jul-Sep
Volume: 78
Issue: 3
First page: 591
Last page: 591
Copyright: © author(s); licensee Österreichische Apotheker-Verlagsgesellschaft m. b. H., Vienna, Austria.
Copyright year: 2010
License: This is an Open Access article distributed under the terms of the Creative Commons Attribution License (http://creativecommons.org/licenses/by/3.0/), which permits unrestricted use, distribution, and reproduction in any medium, provided the original work is properly cited.

JOURNAL INFORMATION
==============================
NLM Title Abbreviation: Sci Pharm
Journal ID: Sci Pharm
NLM Journal ID: 0026251
Journal ID: Scientia Pharmaceutica

Scientia Pharmaceutica

ISSN: 0036-8709
EISSN: 2218-0532
Publisher: Österreichische Apotheker-Verlagsgesellschaft, m. b. H.

ARTICLE INFORMATION
==============================
DOI: 10.3797/scipharm.cespt.8.PDD03
Article ID: scipharm.2010.78.592
Subjects: Conference abstract PDD03

Preparation of Recombinant Human Hydroxysteroid Dehydrogenases and Study of their Inhibitors

Brožič P. 1
Gobec S. 2
Rižner T. Lanišnik 3
1 Sandoz Development Center, Ljubljana, Slovenia (work was performed at 3 )
2 Faculty of Pharmacy, University of Ljubljana, Ljubljana, Slovenia
3 Institute of Biochemistry, Faculty of Medicine, University of Ljubljana, Ljubljana, Slovenia
E-mails: petra.brozic@sandoz.com (P. Brožič), stanislav.gobec@ffa.uni-lj.si (S. Gobec), tea.lanisnik-rizner@mf.uni-lj.si (T. Lanišnik Rižner)
Electronic publication date: 2010 Sep 30
Print publication date: 2010 Jul-Sep
Volume: 78
Issue: 3
First page: 592
Last page: 592
Copyright: © author(s); licensee Österreichische Apotheker-Verlagsgesellschaft m. b. H., Vienna, Austria.
Copyright year: 2010
License: This is an Open Access article distributed under the terms of the Creative Commons Attribution License (http://creativecommons.org/licenses/by/3.0/), which permits unrestricted use, distribution, and reproduction in any medium, provided the original work is properly cited.

JOURNAL INFORMATION
==============================
NLM Title Abbreviation: Sci Pharm
Journal ID: Sci Pharm
NLM Journal ID: 0026251
Journal ID: Scientia Pharmaceutica

Scientia Pharmaceutica

ISSN: 0036-8709
EISSN: 2218-0532
Publisher: Österreichische Apotheker-Verlagsgesellschaft, m. b. H.

ARTICLE INFORMATION
==============================
DOI: 10.3797/scipharm.cespt.8.PDD04
Article ID: scipharm.2010.78.593
Subjects: Conference abstract PDD04

Stabilization of Human Growth Hormone Against Deamidation by Interaction with Strongly Basic Protamines from Salmon

Ablinger E. 1 2
Wegscheider S. 2
Pavkov-Keller T. 3
Keller W. 3
Prassl R. 4
Zimmer A. 2
1 Research Center Pharmaceutical Engineering, Graz, Austria
2 Institute of Pharmaceutical Sciences, Department of Pharmaceutical Technology, University of Graz, Graz, Austria
3 Institute of Molecular Biosciences. Structural Biology, University of Graz, Graz, Austria
4 Institute of Biophysics and Nanosystems Research, Austrian Academy of Sciences, Graz, Austria
E-mail: elisabeth.ablinger@tugraz.at (E. Ablinger)
Electronic publication date: 2010 Sep 30
Print publication date: 2010 Jul-Sep
Volume: 78
Issue: 3
First page: 593
Last page: 593
Copyright: © author(s); licensee Österreichische Apotheker-Verlagsgesellschaft m. b. H., Vienna, Austria.
Copyright year: 2010
License: This is an Open Access article distributed under the terms of the Creative Commons Attribution License (http://creativecommons.org/licenses/by/3.0/), which permits unrestricted use, distribution, and reproduction in any medium, provided the original work is properly cited.

This work was supported by FFG, Land Steiermark and SFG.

JOURNAL INFORMATION
==============================
NLM Title Abbreviation: Sci Pharm
Journal ID: Sci Pharm
NLM Journal ID: 0026251
Journal ID: Scientia Pharmaceutica

Scientia Pharmaceutica

ISSN: 0036-8709
EISSN: 2218-0532
Publisher: Österreichische Apotheker-Verlagsgesellschaft, m. b. H.

ARTICLE INFORMATION
==============================
DOI: 10.3797/scipharm.cespt.8.PDD05
Article ID: scipharm.2010.78.594
Subjects: Conference abstract PDD05

Human Growth Hormone – Insights into its Aggregation Behavior using Denaturation Pathway Characterization

Wiesbauer J. 1 2
Leitgeb S. 1 2
Nidetzky B. 2
1 Research Center Pharmaceutical Engineering GmbH, Graz, Austria
2 Institute of Biotechnology and Biochemical Engineering, University of Technology Graz, Graz, Austria
E-mail: johanna.wiesbauer@rcpe.at (J. Wiesbauer)
Electronic publication date: 2010 Sep 30
Print publication date: 2010 Jul-Sep
Volume: 78
Issue: 3
First page: 594
Last page: 594
Copyright: © author(s); licensee Österreichische Apotheker-Verlagsgesellschaft m. b. H., Vienna, Austria.
Copyright year: 2010
License: This is an Open Access article distributed under the terms of the Creative Commons Attribution License (http://creativecommons.org/licenses/by/3.0/), which permits unrestricted use, distribution, and reproduction in any medium, provided the original work is properly cited.

JOURNAL INFORMATION
==============================
NLM Title Abbreviation: Sci Pharm
Journal ID: Sci Pharm
NLM Journal ID: 0026251
Journal ID: Scientia Pharmaceutica

Scientia Pharmaceutica

ISSN: 0036-8709
EISSN: 2218-0532
Publisher: Österreichische Apotheker-Verlagsgesellschaft, m. b. H.

ARTICLE INFORMATION
==============================
DOI: 10.3797/scipharm.cespt.8.PDD06
Article ID: scipharm.2010.78.595
Subjects: Conference abstract PDD06

Nanostructured Dispersions as Delivery Systems

Chemelli A.
Glatter O.
Department of Chemistry, Karl-Franzens University, Graz, Austria
E-mail: angela.chemelli@uni-graz.at (A. Chemelli)
Electronic publication date: 2010 Sep 30
Print publication date: 2010 Jul-Sep
Volume: 78
Issue: 3
First page: 595
Last page: 595
Copyright: © author(s); licensee Österreichische Apotheker-Verlagsgesellschaft m. b. H., Vienna, Austria.
Copyright year: 2010
License: This is an Open Access article distributed under the terms of the Creative Commons Attribution License (http://creativecommons.org/licenses/by/3.0/), which permits unrestricted use, distribution, and reproduction in any medium, provided the original work is properly cited.

JOURNAL INFORMATION
==============================
NLM Title Abbreviation: Sci Pharm
Journal ID: Sci Pharm
NLM Journal ID: 0026251
Journal ID: Scientia Pharmaceutica

Scientia Pharmaceutica

ISSN: 0036-8709
EISSN: 2218-0532
Publisher: Österreichische Apotheker-Verlagsgesellschaft, m. b. H.

ARTICLE INFORMATION
==============================
DOI: 10.3797/scipharm.cespt.8.PDD07
Article ID: scipharm.2010.78.596
Subjects: Conference abstract PDD07

In Vitro Characterisation of Vancomycin Loaded Glyceryl Monooleate-Water Liquid Crystalline Gel/Implants for Ocular Application

Milak S. 1
Pardeike J. 1
Chemelli A. 2
Glatter O. 2
Zimmer A. 1
1 Institute for Pharmaceutical Sciences, University of Graz, Graz, Austria
2 Institute for Chemistry, University of Graz, Graz, Austria
E-mail: spomenka.milak@edu.uni-graz.at (S. Milak)
Electronic publication date: 2010 Sep 30
Print publication date: 2010 Jul-Sep
Volume: 78
Issue: 3
First page: 596
Last page: 596
Copyright: © author(s); licensee Österreichische Apotheker-Verlagsgesellschaft m. b. H., Vienna, Austria.
Copyright year: 2010
License: This is an Open Access article distributed under the terms of the Creative Commons Attribution License (http://creativecommons.org/licenses/by/3.0/), which permits unrestricted use, distribution, and reproduction in any medium, provided the original work is properly cited.

JOURNAL INFORMATION
==============================
NLM Title Abbreviation: Sci Pharm
Journal ID: Sci Pharm
NLM Journal ID: 0026251
Journal ID: Scientia Pharmaceutica

Scientia Pharmaceutica

ISSN: 0036-8709
EISSN: 2218-0532
Publisher: Österreichische Apotheker-Verlagsgesellschaft, m. b. H.

ARTICLE INFORMATION
==============================
DOI: 10.3797/scipharm.cespt.8.PDD08
Article ID: scipharm.2010.78.597
Subjects: Conference abstract PDD08

In Vitro and In Vivo Evaluation of Drug Release from Semisolid Dosage Forms

Petró É. 1
Csóka I. 1
Balogh Á. 2
Blazsó G. 2
Erős I. 3
1 Department of Drug Regulatory Affairs, University of Szeged, Szeged, Hungary
2 Department of Pharmacodynamics and Biopharmacy, University of Szeged, Szeged, Hungary
3 Department of Pharmaceutical Technology, University of Szeged, Szeged, Hungary
E-mails: petro@pharm.u-szeged.hu (É. Petró), csoka@pharm.u-szeged.hu (I. Csóka), eros@pharm.u-szeged.hu (I. Erős)
Electronic publication date: 2010 Sep 30
Print publication date: 2010 Jul-Sep
Volume: 78
Issue: 3
First page: 597
Last page: 597
Copyright: © author(s); licensee Österreichische Apotheker-Verlagsgesellschaft m. b. H., Vienna, Austria.
Copyright year: 2010
License: This is an Open Access article distributed under the terms of the Creative Commons Attribution License (http://creativecommons.org/licenses/by/3.0/), which permits unrestricted use, distribution, and reproduction in any medium, provided the original work is properly cited.

Fig. 1 In vitro drug release and penetration of 1% diclofenac sodium containing 1% Carbomer 934 P gel (DC1)

Fig. 2 In vivo paw edema test of 1 % diclofenac sodium containing 1% Carbomer 934 P(DC1)

JOURNAL INFORMATION
==============================
NLM Title Abbreviation: Sci Pharm
Journal ID: Sci Pharm
NLM Journal ID: 0026251
Journal ID: Scientia Pharmaceutica

Scientia Pharmaceutica

ISSN: 0036-8709
EISSN: 2218-0532
Publisher: Österreichische Apotheker-Verlagsgesellschaft, m. b. H.

ARTICLE INFORMATION
==============================
DOI: 10.3797/scipharm.cespt.8.PDD09
Article ID: scipharm.2010.78.598
Subjects: Conference abstract PDD09

Gel Emulsions as Controlled Drug Delivery Systems

Budai-Szűcs M.
Erős I.
Department of Pharmaceutical Technology, University of Szeged, H-6720 Szeged
E-mail: eros@pharm.u-szeged.hu (E. Eros)
Electronic publication date: 2010 Sep 30
Print publication date: 2010 Jul-Sep
Volume: 78
Issue: 3
First page: 598
Last page: 598
Copyright: © author(s); licensee Österreichische Apotheker-Verlagsgesellschaft m. b. H., Vienna, Austria.
Copyright year: 2010
License: This is an Open Access article distributed under the terms of the Creative Commons Attribution License (http://creativecommons.org/licenses/by/3.0/), which permits unrestricted use, distribution, and reproduction in any medium, provided the original work is properly cited.

JOURNAL INFORMATION
==============================
NLM Title Abbreviation: Sci Pharm
Journal ID: Sci Pharm
NLM Journal ID: 0026251
Journal ID: Scientia Pharmaceutica

Scientia Pharmaceutica

ISSN: 0036-8709
EISSN: 2218-0532
Publisher: Österreichische Apotheker-Verlagsgesellschaft, m. b. H.

ARTICLE INFORMATION
==============================
DOI: 10.3797/scipharm.cespt.8.PDD10
Article ID: scipharm.2010.78.599
Subjects: Conference abstract PDD10

The Study of Flowing Properties of Hydrogels

Herdová P.
Vitková Z.
Faculty of Pharmacy, Commenius University, Bratislava, Slovakia
E-mail: herdova@fpharm.uniba.sk (P. Herdová)
Electronic publication date: 2010 Sep 30
Print publication date: 2010 Jul-Sep
Volume: 78
Issue: 3
First page: 599
Last page: 599
Copyright: © author(s); licensee Österreichische Apotheker-Verlagsgesellschaft m. b. H., Vienna, Austria.
Copyright year: 2010
License: This is an Open Access article distributed under the terms of the Creative Commons Attribution License (http://creativecommons.org/licenses/by/3.0/), which permits unrestricted use, distribution, and reproduction in any medium, provided the original work is properly cited.

JOURNAL INFORMATION
==============================
NLM Title Abbreviation: Sci Pharm
Journal ID: Sci Pharm
NLM Journal ID: 0026251
Journal ID: Scientia Pharmaceutica

Scientia Pharmaceutica

ISSN: 0036-8709
EISSN: 2218-0532
Publisher: Österreichische Apotheker-Verlagsgesellschaft, m. b. H.

ARTICLE INFORMATION
==============================
DOI: 10.3797/scipharm.cespt.8.PDD11
Article ID: scipharm.2010.78.600
Subjects: Conference abstract PDD11

A New Microemulsion for the Transdermal Delivery of Quercetin

Malaj L. 1
Martena V. 2
Giovenali S. 2
Di Martino P. 2
1 University of Tirana, Tirana, Albania
2 University of Camerino, Camerino, Italy
E-mail: piera.dimartino@unicam.it (P. Di Martino).
Electronic publication date: 2010 Sep 30
Print publication date: 2010 Jul-Sep
Volume: 78
Issue: 3
First page: 600
Last page: 600
Copyright: © author(s); licensee Österreichische Apotheker-Verlagsgesellschaft m. b. H., Vienna, Austria.
Copyright year: 2010
License: This is an Open Access article distributed under the terms of the Creative Commons Attribution License (http://creativecommons.org/licenses/by/3.0/), which permits unrestricted use, distribution, and reproduction in any medium, provided the original work is properly cited.

JOURNAL INFORMATION
==============================
NLM Title Abbreviation: Sci Pharm
Journal ID: Sci Pharm
NLM Journal ID: 0026251
Journal ID: Scientia Pharmaceutica

Scientia Pharmaceutica

ISSN: 0036-8709
EISSN: 2218-0532
Publisher: Österreichische Apotheker-Verlagsgesellschaft, m. b. H.

ARTICLE INFORMATION
==============================
DOI: 10.3797/scipharm.cespt.8.PDD12
Article ID: scipharm.2010.78.601
Subjects: Conference abstract PDD12

In Vitro Release Behavior of Naproxen in Alginate–Chitosan Microparticles as Oral Drug Delivery Systems

Čalija B. 1
Cekić N. 2
Savić S. 1
Milić J. 1
1 Faculty of Pharmacy, Belgrade University, Belgrade, Serbia
2 DCP Hemigal, Leskovac, Serbia
E-mail: bojanc@pharmacy.bg.ac.rs (B. Čalija)
Electronic publication date: 2010 Sep 30
Print publication date: 2010 Jul-Sep
Volume: 78
Issue: 3
First page: 601
Last page: 601
Copyright: © author(s); licensee Österreichische Apotheker-Verlagsgesellschaft m. b. H., Vienna, Austria.
Copyright year: 2010
License: This is an Open Access article distributed under the terms of the Creative Commons Attribution License (http://creativecommons.org/licenses/by/3.0/), which permits unrestricted use, distribution, and reproduction in any medium, provided the original work is properly cited.

JOURNAL INFORMATION
==============================
NLM Title Abbreviation: Sci Pharm
Journal ID: Sci Pharm
NLM Journal ID: 0026251
Journal ID: Scientia Pharmaceutica

Scientia Pharmaceutica

ISSN: 0036-8709
EISSN: 2218-0532
Publisher: Österreichische Apotheker-Verlagsgesellschaft, m. b. H.

ARTICLE INFORMATION
==============================
DOI: 10.3797/scipharm.cespt.8.PDD13
Article ID: scipharm.2010.78.602
Subjects: Conference abstract PDD13

Study of Formulation Factors Influencing Phenytoin Release Rate from Alginate–Chitosan Microparticles using an Experimental Design Approach

Cekić N. 1
Čalija B. 2
Savić S. 2
Milić J. 2
1 DCP Hemigal, Leskovac, Serbia
2 Faculty of Pharmacy, Belgrade University, Belgrade, Serbia
E-mail: nesafarm@gmail.com (N. Cekić)
Electronic publication date: 2010 Sep 30
Print publication date: 2010 Jul-Sep
Volume: 78
Issue: 3
First page: 602
Last page: 602
Copyright: © author(s); licensee Österreichische Apotheker-Verlagsgesellschaft m. b. H., Vienna, Austria.
Copyright year: 2010
License: This is an Open Access article distributed under the terms of the Creative Commons Attribution License (http://creativecommons.org/licenses/by/3.0/), which permits unrestricted use, distribution, and reproduction in any medium, provided the original work is properly cited.

JOURNAL INFORMATION
==============================
NLM Title Abbreviation: Sci Pharm
Journal ID: Sci Pharm
NLM Journal ID: 0026251
Journal ID: Scientia Pharmaceutica

Scientia Pharmaceutica

ISSN: 0036-8709
EISSN: 2218-0532
Publisher: Österreichische Apotheker-Verlagsgesellschaft, m. b. H.

ARTICLE INFORMATION
==============================
DOI: 10.3797/scipharm.cespt.8.PDD14
Article ID: scipharm.2010.78.603
Subjects: Conference abstract PDD14

Probing the Surface Properties of Solid Lipid Nanoparticles by Atomic Force Microscopy

Govedarica B.
Teskač K.
Pajk S.
Pečar S.
Srčič S.
Kristl J.
University of Ljubljana, Faculty of Pharmacy, Ljubljana, Slovenia
E-mail: julijana.kristl@ffa.uni-lj.si (J. Kristl)
Electronic publication date: 2010 Sep 30
Print publication date: 2010 Jul-Sep
Volume: 78
Issue: 3
First page: 603
Last page: 603
Copyright: © author(s); licensee Österreichische Apotheker-Verlagsgesellschaft m. b. H., Vienna, Austria.
Copyright year: 2010
License: This is an Open Access article distributed under the terms of the Creative Commons Attribution License (http://creativecommons.org/licenses/by/3.0/), which permits unrestricted use, distribution, and reproduction in any medium, provided the original work is properly cited.

JOURNAL INFORMATION
==============================
NLM Title Abbreviation: Sci Pharm
Journal ID: Sci Pharm
NLM Journal ID: 0026251
Journal ID: Scientia Pharmaceutica

Scientia Pharmaceutica

ISSN: 0036-8709
EISSN: 2218-0532
Publisher: Österreichische Apotheker-Verlagsgesellschaft, m. b. H.

ARTICLE INFORMATION
==============================
DOI: 10.3797/scipharm.cespt.8.PDD15
Article ID: scipharm.2010.78.604
Subjects: Conference abstract PDD15

Microparticles Based on Low Melting Hydrophilic Polymers Prepared by Spray Drying

Homar M.
Kerč J.
Lek Pharmaceuticals,d.d., Sandoz Development Center Slovenia, Verovškova 57, 1526 Ljubljana, Slovenia
E-mail: miha.homar@sandoz.com (M. Homar)
Electronic publication date: 2010 Sep 30
Print publication date: 2010 Jul-Sep
Volume: 78
Issue: 3
First page: 604
Last page: 604
Copyright: © author(s); licensee Österreichische Apotheker-Verlagsgesellschaft m. b. H., Vienna, Austria.
Copyright year: 2010
License: This is an Open Access article distributed under the terms of the Creative Commons Attribution License (http://creativecommons.org/licenses/by/3.0/), which permits unrestricted use, distribution, and reproduction in any medium, provided the original work is properly cited.

JOURNAL INFORMATION
==============================
NLM Title Abbreviation: Sci Pharm
Journal ID: Sci Pharm
NLM Journal ID: 0026251
Journal ID: Scientia Pharmaceutica

Scientia Pharmaceutica

ISSN: 0036-8709
EISSN: 2218-0532
Publisher: Österreichische Apotheker-Verlagsgesellschaft, m. b. H.

ARTICLE INFORMATION
==============================
DOI: 10.3797/scipharm.cespt.8.PDD16
Article ID: scipharm.2010.78.605
Subjects: Conference abstract PDD16

Furosemide-Loaded Microcapsules: The Influence of Lactose Content on Encapsulation Efficiency and Drug Release

Zvonar A.
Gosenca M.
Gašperlin M.
Faculty of pharmacy, University of Ljubljana, Ljubljana, Slovenia
E-mails: alenka.zvonar@ffa.uni-lj.si (A. Zvonar), Mirjam.gosenca@ffa.uni-lj.si (M. Gosenca), mirjana.gasperlin@ffa.uni-lj.si (M. Gašperlin)
Electronic publication date: 2010 Sep 30
Print publication date: 2010 Jul-Sep
Volume: 78
Issue: 3
First page: 605
Last page: 605
Copyright: © author(s); licensee Österreichische Apotheker-Verlagsgesellschaft m. b. H., Vienna, Austria.
Copyright year: 2010
License: This is an Open Access article distributed under the terms of the Creative Commons Attribution License (http://creativecommons.org/licenses/by/3.0/), which permits unrestricted use, distribution, and reproduction in any medium, provided the original work is properly cited.

JOURNAL INFORMATION
==============================
NLM Title Abbreviation: Sci Pharm
Journal ID: Sci Pharm
NLM Journal ID: 0026251
Journal ID: Scientia Pharmaceutica

Scientia Pharmaceutica

ISSN: 0036-8709
EISSN: 2218-0532
Publisher: Österreichische Apotheker-Verlagsgesellschaft, m. b. H.

ARTICLE INFORMATION
==============================
DOI: 10.3797/scipharm.cespt.8.PDD17
Article ID: scipharm.2010.78.606
Subjects: Conference abstract PDD17

Solubilization of Drugs by Aqueous Lecithin Dispersions Intended for Parenteral Use

Sznitowska M.
Placzek M.
Kluk A.
Department of Pharmaceutical Technology, Medical University of Gdansk, Poland
E-mails: msznito@gumed.edu.pl (M. Sznitowska), mpl@gumed.edu.pl (M. Placzek)
Electronic publication date: 2010 Sep 30
Print publication date: 2010 Jul-Sep
Volume: 78
Issue: 3
First page: 606
Last page: 606
Copyright: © author(s); licensee Österreichische Apotheker-Verlagsgesellschaft m. b. H., Vienna, Austria.
Copyright year: 2010
License: This is an Open Access article distributed under the terms of the Creative Commons Attribution License (http://creativecommons.org/licenses/by/3.0/), which permits unrestricted use, distribution, and reproduction in any medium, provided the original work is properly cited.

JOURNAL INFORMATION
==============================
NLM Title Abbreviation: Sci Pharm
Journal ID: Sci Pharm
NLM Journal ID: 0026251
Journal ID: Scientia Pharmaceutica

Scientia Pharmaceutica

ISSN: 0036-8709
EISSN: 2218-0532
Publisher: Österreichische Apotheker-Verlagsgesellschaft, m. b. H.

ARTICLE INFORMATION
==============================
DOI: 10.3797/scipharm.cespt.8.PDD18
Article ID: scipharm.2010.78.607
Subjects: Conference abstract PDD18

Dissolution Rate of Microencapsulated Active Substances

Erős I.
Péter D.
Sipos P.
Kása P.
Hódi K.
Department of Pharmaceutical technology, University of Szeged, Hungary
E-mails: eros@pharm.u-szeged.hu (I. Erős), hodi@pharm.u-szeged.hu (K. Hódi)
Electronic publication date: 2010 Sep 30
Print publication date: 2010 Jul-Sep
Volume: 78
Issue: 3
First page: 607
Last page: 607
Copyright: © author(s); licensee Österreichische Apotheker-Verlagsgesellschaft m. b. H., Vienna, Austria.
Copyright year: 2010
License: This is an Open Access article distributed under the terms of the Creative Commons Attribution License (http://creativecommons.org/licenses/by/3.0/), which permits unrestricted use, distribution, and reproduction in any medium, provided the original work is properly cited.

Fig. 1. Flow diagram

JOURNAL INFORMATION
==============================
NLM Title Abbreviation: Sci Pharm
Journal ID: Sci Pharm
NLM Journal ID: 0026251
Journal ID: Scientia Pharmaceutica

Scientia Pharmaceutica

ISSN: 0036-8709
EISSN: 2218-0532
Publisher: Österreichische Apotheker-Verlagsgesellschaft, m. b. H.

ARTICLE INFORMATION
==============================
DOI: 10.3797/scipharm.cespt.8.PDD19
Article ID: scipharm.2010.78.608
Subjects: Conference abstract PDD19

Impact of Particles Size Distribution on Diazepam 5mg Tablets Dissolution

Pilipović B.
Vehabović M.
Hadžović E.
Šarić N.
Bosnalijek d.d. Jukićeva 53, Sarajevo, Bosnia and Herzegovina
E-mail: berina.p@bosnalijek.ba (B. Pilipović)
Electronic publication date: 2010 Sep 30
Print publication date: 2010 Jul-Sep
Volume: 78
Issue: 3
First page: 608
Last page: 608
Copyright: © author(s); licensee Österreichische Apotheker-Verlagsgesellschaft m. b. H., Vienna, Austria.
Copyright year: 2010
License: This is an Open Access article distributed under the terms of the Creative Commons Attribution License (http://creativecommons.org/licenses/by/3.0/), which permits unrestricted use, distribution, and reproduction in any medium, provided the original work is properly cited.

Tab. 1. minutes	Formulation A	Formulation B	
10	15	20	25	30	10	15	20	25	30	
S.V.	85.34	99.05	100.79	100.39	99.88	99,08	98,49	97,90	97,36	96,89	
SD	4.65	2.57	3.20	3.34	3.31	1,32	1,32	1,28	1,27	1,28	
RSD	5.45	2.60	3.17	3.32	3.31	1,33	1,34	1,31	1,31	1,32	
f2					68,15					99,44	

JOURNAL INFORMATION
==============================
NLM Title Abbreviation: Sci Pharm
Journal ID: Sci Pharm
NLM Journal ID: 0026251
Journal ID: Scientia Pharmaceutica

Scientia Pharmaceutica

ISSN: 0036-8709
EISSN: 2218-0532
Publisher: Österreichische Apotheker-Verlagsgesellschaft, m. b. H.

ARTICLE INFORMATION
==============================
DOI: 10.3797/scipharm.cespt.8.PDD20
Article ID: scipharm.2010.78.609
Subjects: Conference abstract PDD20

In Vitro Release of Indomethacin and Hydrocortisone from Suspensions and Self-Emulsifying Oils

Czajkowska-Kośnik A. 1
Sznitowska M. 2
1 Department of Pharmaceutical Technology, Medical University of Bialystok, Poland
2 Department of Pharmaceutical Technology, Medical University of Gdansk, Poland
E-mails: aczaj@umb.edu.pl (A. Czajkowska-Kośnik), msznito@gumed.edu.pl (M. Sznitowska)
Electronic publication date: 2010 Sep 30
Print publication date: 2010 Jul-Sep
Volume: 78
Issue: 3
First page: 609
Last page: 609
Copyright: © author(s); licensee Österreichische Apotheker-Verlagsgesellschaft m. b. H., Vienna, Austria.
Copyright year: 2010
License: This is an Open Access article distributed under the terms of the Creative Commons Attribution License (http://creativecommons.org/licenses/by/3.0/), which permits unrestricted use, distribution, and reproduction in any medium, provided the original work is properly cited.

JOURNAL INFORMATION
==============================
NLM Title Abbreviation: Sci Pharm
Journal ID: Sci Pharm
NLM Journal ID: 0026251
Journal ID: Scientia Pharmaceutica

Scientia Pharmaceutica

ISSN: 0036-8709
EISSN: 2218-0532
Publisher: Österreichische Apotheker-Verlagsgesellschaft, m. b. H.

ARTICLE INFORMATION
==============================
DOI: 10.3797/scipharm.cespt.8.PDD21
Article ID: scipharm.2010.78.610
Subjects: Conference abstract PDD21

Influence of Dissolution Media Composition on Cefaclor Release form Capsules

Spasić A.
Homšek I.
R&D Institute, Galenika a.d., Batajnički drium b.b., 11080 Belgrade, Serbia
E-mail: sandraspasic80@yahoo.com (A. Spasic)
Electronic publication date: 2010 Sep 30
Print publication date: 2010 Jul-Sep
Volume: 78
Issue: 3
First page: 610
Last page: 610
Copyright: © author(s); licensee Österreichische Apotheker-Verlagsgesellschaft m. b. H., Vienna, Austria.
Copyright year: 2010
License: This is an Open Access article distributed under the terms of the Creative Commons Attribution License (http://creativecommons.org/licenses/by/3.0/), which permits unrestricted use, distribution, and reproduction in any medium, provided the original work is properly cited.

JOURNAL INFORMATION
==============================
NLM Title Abbreviation: Sci Pharm
Journal ID: Sci Pharm
NLM Journal ID: 0026251
Journal ID: Scientia Pharmaceutica

Scientia Pharmaceutica

ISSN: 0036-8709
EISSN: 2218-0532
Publisher: Österreichische Apotheker-Verlagsgesellschaft, m. b. H.

ARTICLE INFORMATION
==============================
DOI: 10.3797/scipharm.cespt.8.PDD22
Article ID: scipharm.2010.78.611
Subjects: Conference abstract PDD22

In Vitro Evaluation of Extended Release HPMC Matrix Tablets and Correlation with In Vivo Data

Klančar U.
Legen I.
Horvat M.
Lek Pharmaceuticals d.d., Sandoz Development Center Slovenia, Verovškova 57, SI-1526 Ljubljana, Slovenia
E-mail: uros.klancar@sandoz.com (U. Klancar)
Electronic publication date: 2010 Sep 30
Print publication date: 2010 Jul-Sep
Volume: 78
Issue: 3
First page: 611
Last page: 611
Copyright: © author(s); licensee Österreichische Apotheker-Verlagsgesellschaft m. b. H., Vienna, Austria.
Copyright year: 2010
License: This is an Open Access article distributed under the terms of the Creative Commons Attribution License (http://creativecommons.org/licenses/by/3.0/), which permits unrestricted use, distribution, and reproduction in any medium, provided the original work is properly cited.

JOURNAL INFORMATION
==============================
NLM Title Abbreviation: Sci Pharm
Journal ID: Sci Pharm
NLM Journal ID: 0026251
Journal ID: Scientia Pharmaceutica

Scientia Pharmaceutica

ISSN: 0036-8709
EISSN: 2218-0532
Publisher: Österreichische Apotheker-Verlagsgesellschaft, m. b. H.

ARTICLE INFORMATION
==============================
DOI: 10.3797/scipharm.cespt.8.PDD23
Article ID: scipharm.2010.78.612
Subjects: Conference abstract PDD23

Release of Theophylline from Sustained Release Cylindrical Dosage Forms

Bogataj M. 1
Ružić B. 1 2
Perissutti B. 2
Voinovich D. 2
Mrhar A. 1
1 Faculty of Pharmacy, University of Ljubljana, Aškerčeva 7, 1000 Ljubljana, Slovenia
2 Faculty of Pharmacy, University of Trieste, Piazzale Europa 1, 34000 Trieste, Italy
E-mails: marija.bogataj@ffa.uni-lj.si (M. Bogataj), blanka.ruzic@ri.t-com.hr (B. Ružić)
Electronic publication date: 2010 Sep 30
Print publication date: 2010 Jul-Sep
Volume: 78
Issue: 3
First page: 612
Last page: 612
Copyright: © author(s); licensee Österreichische Apotheker-Verlagsgesellschaft m. b. H., Vienna, Austria.
Copyright year: 2010
License: This is an Open Access article distributed under the terms of the Creative Commons Attribution License (http://creativecommons.org/licenses/by/3.0/), which permits unrestricted use, distribution, and reproduction in any medium, provided the original work is properly cited.

Fig. 1. Percentage of released drug after 7h of release in dependence on stirring rate

Fig. 2. Stereomicroscopic view of co-extrudates. Arrow shows void space between two parts of cylinder.

JOURNAL INFORMATION
==============================
NLM Title Abbreviation: Sci Pharm
Journal ID: Sci Pharm
NLM Journal ID: 0026251
Journal ID: Scientia Pharmaceutica

Scientia Pharmaceutica

ISSN: 0036-8709
EISSN: 2218-0532
Publisher: Österreichische Apotheker-Verlagsgesellschaft, m. b. H.

ARTICLE INFORMATION
==============================
DOI: 10.3797/scipharm.cespt.8.PDD24
Article ID: scipharm.2010.78.613
Subjects: Conference abstract PDD24

Formulation Development of Immediate Release Tablets Containing Fluoroquinolone Antibiotic by use of Experimental Design

Božić J.
Đokić M.
Institute Hemofarm, Hemofarm STADA, Vršac, Serbia
E-mails: jovana.bozic@hemofarm.com (J. Božić), marija.djokic@hemofarm.com (M.Đokić)
Electronic publication date: 2010 Sep 30
Print publication date: 2010 Jul-Sep
Volume: 78
Issue: 3
First page: 613
Last page: 613
Copyright: © author(s); licensee Österreichische Apotheker-Verlagsgesellschaft m. b. H., Vienna, Austria.
Copyright year: 2010
License: This is an Open Access article distributed under the terms of the Creative Commons Attribution License (http://creativecommons.org/licenses/by/3.0/), which permits unrestricted use, distribution, and reproduction in any medium, provided the original work is properly cited.

JOURNAL INFORMATION
==============================
NLM Title Abbreviation: Sci Pharm
Journal ID: Sci Pharm
NLM Journal ID: 0026251
Journal ID: Scientia Pharmaceutica

Scientia Pharmaceutica

ISSN: 0036-8709
EISSN: 2218-0532
Publisher: Österreichische Apotheker-Verlagsgesellschaft, m. b. H.

ARTICLE INFORMATION
==============================
DOI: 10.3797/scipharm.cespt.8.PDD25
Article ID: scipharm.2010.78.614
Subjects: Conference abstract PDD25

Influence of Caloric Value of Food on Gastric Emptying of Pellets

Bürmen B.
Locatelli I.
Mrhar A.
Bogataj M.
Faculty of Pharmacy, University of Ljubljana, Aškerčeva cesta 7, SI-1000 Ljubljana, Slovenia
E-mail: igor.locatelli@ffa.uni-lj.si (I. Locatelli)
Electronic publication date: 2010 Sep 30
Print publication date: 2010 Jul-Sep
Volume: 78
Issue: 3
First page: 614
Last page: 614
Copyright: © author(s); licensee Österreichische Apotheker-Verlagsgesellschaft m. b. H., Vienna, Austria.
Copyright year: 2010
License: This is an Open Access article distributed under the terms of the Creative Commons Attribution License (http://creativecommons.org/licenses/by/3.0/), which permits unrestricted use, distribution, and reproduction in any medium, provided the original work is properly cited.

JOURNAL INFORMATION
==============================
NLM Title Abbreviation: Sci Pharm
Journal ID: Sci Pharm
NLM Journal ID: 0026251
Journal ID: Scientia Pharmaceutica

Scientia Pharmaceutica

ISSN: 0036-8709
EISSN: 2218-0532
Publisher: Österreichische Apotheker-Verlagsgesellschaft, m. b. H.

ARTICLE INFORMATION
==============================
DOI: 10.3797/scipharm.cespt.8.PDD26
Article ID: scipharm.2010.78.615
Subjects: Conference abstract PDD26

Development of Micropellets for Buccal Drug Delivery

Schrank S. 1 2
Khinast J. 2 3
Zimmer A. 1
Roblegg E. 1 3
1 Institute for Pharmaceutical Sciences/Department of Pharmaceutical Technology, Karl-Franzens University, Graz, Austria
2 Institute for Process and Particle Engineering, University of Technology, Graz, Austria
3 Research Center Pharmaceutical Engineering GmbH, Graz, Austria
E-mail: simone.schrank@uni-graz.at (S. Schrank)
Electronic publication date: 2010 Sep 30
Print publication date: 2010 Jul-Sep
Volume: 78
Issue: 3
First page: 615
Last page: 615
Copyright: © author(s); licensee Österreichische Apotheker-Verlagsgesellschaft m. b. H., Vienna, Austria.
Copyright year: 2010
License: This is an Open Access article distributed under the terms of the Creative Commons Attribution License (http://creativecommons.org/licenses/by/3.0/), which permits unrestricted use, distribution, and reproduction in any medium, provided the original work is properly cited.

JOURNAL INFORMATION
==============================
NLM Title Abbreviation: Sci Pharm
Journal ID: Sci Pharm
NLM Journal ID: 0026251
Journal ID: Scientia Pharmaceutica

Scientia Pharmaceutica

ISSN: 0036-8709
EISSN: 2218-0532
Publisher: Österreichische Apotheker-Verlagsgesellschaft, m. b. H.

ARTICLE INFORMATION
==============================
DOI: 10.3797/scipharm.cespt.8.PDD27
Article ID: scipharm.2010.78.616
Subjects: Conference abstract PDD27

Gastro-Intestinal Lymphatic Absorption of Sylibum Marianum Formulated inSelf-Emulsifying Pellets

Voinovich D. 1
Serdoz F. 1
Perissutti B. 1
Hasa D. 1
Pinto J. 2
Zara G. P. 3
Bargoni A. 3
Grabnar I. 4
1 Department of Pharmaceutical Sciences, University of Trieste, Trieste, Italy
2 Dep.Tecnologia Farmacêutica, Universidade de Lisboa, Lisboa, Portugal
3 Department of Anatomy, Pharmacology and Forensic Medicine, University of Torino, Torino, Italy
4 Chair of Biopharmaceutics and Pharmacokinetics, University of Ljubljana, Ljubljana, Slovenia
E-mail: vojnovic@units.it (D. Voinovich)
Electronic publication date: 2010 Sep 30
Print publication date: 2010 Jul-Sep
Volume: 78
Issue: 3
First page: 616
Last page: 616
Copyright: © author(s); licensee Österreichische Apotheker-Verlagsgesellschaft m. b. H., Vienna, Austria.
Copyright year: 2010
License: This is an Open Access article distributed under the terms of the Creative Commons Attribution License (http://creativecommons.org/licenses/by/3.0/), which permits unrestricted use, distribution, and reproduction in any medium, provided the original work is properly cited.

The authors thank Indena for the kind gift of Sylibum Marianum dry extract.

JOURNAL INFORMATION
==============================
NLM Title Abbreviation: Sci Pharm
Journal ID: Sci Pharm
NLM Journal ID: 0026251
Journal ID: Scientia Pharmaceutica

Scientia Pharmaceutica

ISSN: 0036-8709
EISSN: 2218-0532
Publisher: Österreichische Apotheker-Verlagsgesellschaft, m. b. H.

ARTICLE INFORMATION
==============================
DOI: 10.3797/scipharm.cespt.8.PDD28
Article ID: scipharm.2010.78.617
Subjects: Conference abstract PDD28

Design and Characterization of Polymeric Blends for Biomedical Applications

Barba A. A. 1
Lamberti G. 2
Rabbia L. 2
Grassi M. 3
Grassi G. 4
Larobina D. 5
1 Dipartimento di Scienze Farmaceutiche, Università di Salerno, Fisciano (SA), Italia
2 Dipartimento di Ingegneria Chimica e Alimentare, Università di Salerno, Fisciano (SA), Italia
3 Dipartimento dei Materiali e delle Risorse Naturali, Università di Trieste, Trieste, Italia
4 Dipartimento di Scienze della Vita, Università di Trieste, Trieste, Italia
5 Istituto per i Materiali Compositi e Biomedici, CNR, Portici (NA), Italia
E-mails: aabarba@unisa.it (A.A. Barba), glamberti@unisa.it (G. Lamberti), mario.grassi@dicamp.units.it (M. Grassi), ggrassi@units.it (G. Grassi), larobina@unina.it (D. Larobina)
Electronic publication date: 2010 Sep 30
Print publication date: 2010 Jul-Sep
Volume: 78
Issue: 3
First page: 617
Last page: 617
Copyright: © author(s); licensee Österreichische Apotheker-Verlagsgesellschaft m. b. H., Vienna, Austria.
Copyright year: 2010
License: This is an Open Access article distributed under the terms of the Creative Commons Attribution License (http://creativecommons.org/licenses/by/3.0/), which permits unrestricted use, distribution, and reproduction in any medium, provided the original work is properly cited.

JOURNAL INFORMATION
==============================
NLM Title Abbreviation: Sci Pharm
Journal ID: Sci Pharm
NLM Journal ID: 0026251
Journal ID: Scientia Pharmaceutica

Scientia Pharmaceutica

ISSN: 0036-8709
EISSN: 2218-0532
Publisher: Österreichische Apotheker-Verlagsgesellschaft, m. b. H.

ARTICLE INFORMATION
==============================
DOI: 10.3797/scipharm.cespt.8.PDD29
Article ID: scipharm.2010.78.618
Subjects: Conference abstract PDD29

Effect of Polymer Charge on Permeability of Urinary Bladder Wall

Kerec Kos M.
Bogataj M.
Mrhar A.
Faculty of Pharmacy, University of Ljubljana, Aškerčeva 7, 1000 Ljubljana, Slovenia
E-mail: kerecm@ffa.uni-lj.si (M. Kerec Kos)
Electronic publication date: 2010 Sep 30
Print publication date: 2010 Jul-Sep
Volume: 78
Issue: 3
First page: 618
Last page: 618
Copyright: © author(s); licensee Österreichische Apotheker-Verlagsgesellschaft m. b. H., Vienna, Austria.
Copyright year: 2010
License: This is an Open Access article distributed under the terms of the Creative Commons Attribution License (http://creativecommons.org/licenses/by/3.0/), which permits unrestricted use, distribution, and reproduction in any medium, provided the original work is properly cited.

JOURNAL INFORMATION
==============================
NLM Title Abbreviation: Sci Pharm
Journal ID: Sci Pharm
NLM Journal ID: 0026251
Journal ID: Scientia Pharmaceutica

Scientia Pharmaceutica

ISSN: 0036-8709
EISSN: 2218-0532
Publisher: Österreichische Apotheker-Verlagsgesellschaft, m. b. H.

ARTICLE INFORMATION
==============================
DOI: 10.3797/scipharm.cespt.8.PDD30
Article ID: scipharm.2010.78.619
Subjects: Conference abstract PDD30

Membrane-Mediated Effects of General Anesthetics on Ion Channels

Pabst G. 1
Jerabek H. 2
Rappolt M. 1
Stockner T. 2 3
1 Institute of Biophysics and Nanosystems Research, Austrian Academy of Sciences, Graz, Austria
2 Department of Health & Environment, Austrian Institute of Technology, Seibersdorf, Austria
3 Department of Medical Chemistry, Medical University of Vienna, Vienna, Austria
E-mails: georg.pabst@oeaw.ac.at (G. Pabst), thomas.stockner@meduniwien.ac.at (T. Stockner)
Electronic publication date: 2010 Sep 30
Print publication date: 2010 Jul-Sep
Volume: 78
Issue: 3
First page: 619
Last page: 619
Copyright: © author(s); licensee Österreichische Apotheker-Verlagsgesellschaft m. b. H., Vienna, Austria.
Copyright year: 2010
License: This is an Open Access article distributed under the terms of the Creative Commons Attribution License (http://creativecommons.org/licenses/by/3.0/), which permits unrestricted use, distribution, and reproduction in any medium, provided the original work is properly cited.

JOURNAL INFORMATION
==============================
NLM Title Abbreviation: Sci Pharm
Journal ID: Sci Pharm
NLM Journal ID: 0026251
Journal ID: Scientia Pharmaceutica

Scientia Pharmaceutica

ISSN: 0036-8709
EISSN: 2218-0532
Publisher: Österreichische Apotheker-Verlagsgesellschaft, m. b. H.

ARTICLE INFORMATION
==============================
DOI: 10.3797/scipharm.cespt.8.PDD31
Article ID: scipharm.2010.78.620
Subjects: Conference abstract PDD31

Intra-Articular Drug Delivery Systems for Osteoarthritis Treatment

Pradal J. 1
Jordan O. 1
Gabay C. 2
Guerne P. A. 2
Bagley M. C. 3
Doelker E. 1
Waldburger J. M. 2
Allémann E. 1
1 School of Pharmaceutical Sciences, University of Geneva, University of Lausanne, Geneva, Switzerland
2 Division of Rheumatology, Department of Internal Medicine, University Hospital, 26 avenue Beau-Séjour, 1211 Geneva 14, Switzerland and Department of Pathology and Immunology, University of Geneva School of Medicine, 1 rue Michel-Servet, 1211 Geneva 4, Switzerland
3 School of Chemistry, Main Building, Cardiff University, Park Place, Cardiff CF10 3AT, United Kingdom
E-mails: julie.pradal@unige.ch (J. Pradal), eric.allemann@unige.ch (E. Allémann)
Electronic publication date: 2010 Sep 30
Print publication date: 2010 Jul-Sep
Volume: 78
Issue: 3
First page: 620
Last page: 620
Copyright: © author(s); licensee Österreichische Apotheker-Verlagsgesellschaft m. b. H., Vienna, Austria.
Copyright year: 2010
License: This is an Open Access article distributed under the terms of the Creative Commons Attribution License (http://creativecommons.org/licenses/by/3.0/), which permits unrestricted use, distribution, and reproduction in any medium, provided the original work is properly cited.

JOURNAL INFORMATION
==============================
NLM Title Abbreviation: Sci Pharm
Journal ID: Sci Pharm
NLM Journal ID: 0026251
Journal ID: Scientia Pharmaceutica

Scientia Pharmaceutica

ISSN: 0036-8709
EISSN: 2218-0532
Publisher: Österreichische Apotheker-Verlagsgesellschaft, m. b. H.

ARTICLE INFORMATION
==============================
DOI: 10.3797/scipharm.cespt.8.PDD32
Article ID: scipharm.2010.78.621
Subjects: Conference abstract PDD32

Case by Case Physicochemical and In Vitro/In Vivo Biopharmaceutical Characterization of Alkyl Polyglucoside-Based Vehicles: Ketoprofen as a Model Drug

Jaksic I. 1
Lukic M. 1
Daniels R. 2
Reichel S. 3
Milic J. 1
Savic S. 1
1 Department of Pharmaceutical Technology and Cosmetology, University of Belgrade, Belgrade, Serbia
2 Department of Pharmaceutical Technology, Eberhard-Karls Universität Tübingen, Tübingen, Germany
3 Department of Pharmaceutical Technology, Technische Universität Braunschweig, Braunschweig, Germany
E-mail: ijaksic@pharmacy.bg.ac.rs (I. Jaksic)
Electronic publication date: 2010 Sep 30
Print publication date: 2010 Jul-Sep
Volume: 78
Issue: 3
First page: 621
Last page: 621
Copyright: © author(s); licensee Österreichische Apotheker-Verlagsgesellschaft m. b. H., Vienna, Austria.
Copyright year: 2010
License: This is an Open Access article distributed under the terms of the Creative Commons Attribution License (http://creativecommons.org/licenses/by/3.0/), which permits unrestricted use, distribution, and reproduction in any medium, provided the original work is properly cited.

JOURNAL INFORMATION
==============================
NLM Title Abbreviation: Sci Pharm
Journal ID: Sci Pharm
NLM Journal ID: 0026251
Journal ID: Scientia Pharmaceutica

Scientia Pharmaceutica

ISSN: 0036-8709
EISSN: 2218-0532
Publisher: Österreichische Apotheker-Verlagsgesellschaft, m. b. H.

ARTICLE INFORMATION
==============================
DOI: 10.3797/scipharm.cespt.8.PDD33
Article ID: scipharm.2010.78.622
Subjects: Conference abstract PDD33

Spontaneous Formation of Theophylline–Nicotinamide Cocrystals

Ervasti T.
Ketolainen J.
Aaltonen J.
School of Pharmacy, Pharmaceutical Technology, Faculty of Health Sciences, University of Eastern Finland, Kuopio Campus, Finland
E-mail: Tuomas.Ervasti@uef.fi (T. Ervasti)
Electronic publication date: 2010 Sep 30
Print publication date: 2010 Jul-Sep
Volume: 78
Issue: 3
First page: 622
Last page: 622
Copyright: © author(s); licensee Österreichische Apotheker-Verlagsgesellschaft m. b. H., Vienna, Austria.
Copyright year: 2010
License: This is an Open Access article distributed under the terms of the Creative Commons Attribution License (http://creativecommons.org/licenses/by/3.0/), which permits unrestricted use, distribution, and reproduction in any medium, provided the original work is properly cited.

JOURNAL INFORMATION
==============================
NLM Title Abbreviation: Sci Pharm
Journal ID: Sci Pharm
NLM Journal ID: 0026251
Journal ID: Scientia Pharmaceutica

Scientia Pharmaceutica

ISSN: 0036-8709
EISSN: 2218-0532
Publisher: Österreichische Apotheker-Verlagsgesellschaft, m. b. H.

ARTICLE INFORMATION
==============================
DOI: 10.3797/scipharm.cespt.8.PDD34
Article ID: scipharm.2010.78.623
Subjects: Conference abstract PDD34

Electrostatic Charge on Pharmaceutical Powders for Pulmonary Application

Karner S. 1
Urbanetz N. A. 1 2
1 Institute for Process and Particle Engineering, Graz University of technology, Graz, Austria
2 Research Center Pharmaceutical Engineering GmbH, Graz, Austria
E-mails: stefan.karner@tugraz.at (S. Karner), nora.urbanetz@tugraz.at (N. A. Urbanetz)
Electronic publication date: 2010 Sep 30
Print publication date: 2010 Jul-Sep
Volume: 78
Issue: 3
First page: 623
Last page: 623
Copyright: © author(s); licensee Österreichische Apotheker-Verlagsgesellschaft m. b. H., Vienna, Austria.
Copyright year: 2010
License: This is an Open Access article distributed under the terms of the Creative Commons Attribution License (http://creativecommons.org/licenses/by/3.0/), which permits unrestricted use, distribution, and reproduction in any medium, provided the original work is properly cited.

JOURNAL INFORMATION
==============================
NLM Title Abbreviation: Sci Pharm
Journal ID: Sci Pharm
NLM Journal ID: 0026251
Journal ID: Scientia Pharmaceutica

Scientia Pharmaceutica

ISSN: 0036-8709
EISSN: 2218-0532
Publisher: Österreichische Apotheker-Verlagsgesellschaft, m. b. H.

ARTICLE INFORMATION
==============================
DOI: 10.3797/scipharm.cespt.8.PDD35
Article ID: scipharm.2010.78.624
Subjects: Conference abstract PDD35

Characterization of Antifungal Nail Lacquer Formulations Containing Fluconazole

Elezović A. 1
Hadžović S. 1
Hadžidedić Š. 2
Kostić S. 3
1 Faculty of Pharmacy, University of Sarajevo, Sarajevo, Bosnia and Herzegovina
2 Agency for Medicines and Medical Devices, Sarajevo, Bosnia and Herzegovina
3 Bosnalijek, d.d. Sarajevo, Bosnia and Herzegovina
E-mail: akaric@ffsa.unsa.ba (A. Elezović)
Electronic publication date: 2010 Sep 30
Print publication date: 2010 Jul-Sep
Volume: 78
Issue: 3
First page: 624
Last page: 624
Copyright: © author(s); licensee Österreichische Apotheker-Verlagsgesellschaft m. b. H., Vienna, Austria.
Copyright year: 2010
License: This is an Open Access article distributed under the terms of the Creative Commons Attribution License (http://creativecommons.org/licenses/by/3.0/), which permits unrestricted use, distribution, and reproduction in any medium, provided the original work is properly cited.

JOURNAL INFORMATION
==============================
NLM Title Abbreviation: Sci Pharm
Journal ID: Sci Pharm
NLM Journal ID: 0026251
Journal ID: Scientia Pharmaceutica

Scientia Pharmaceutica

ISSN: 0036-8709
EISSN: 2218-0532
Publisher: Österreichische Apotheker-Verlagsgesellschaft, m. b. H.

ARTICLE INFORMATION
==============================
DOI: 10.3797/scipharm.cespt.8.PDD36
Article ID: scipharm.2010.78.625
Subjects: Conference abstract PDD36

Modelling Swelling, Erosion and Release of Drug from Hydrogel Based Solid Matrices

Lamberti G. 1
Galdi I. 1
Titomanlio G. 1
Barba A. A. 2
d’Amore M. 2
1 Dipartimento di Ingegneria Chimica e Alimentare, Università di Salerno, Fisciano, Italia
2 Dipartimento di Scienze Farmaceutiche, Università di Salerno, Fisciano, Italia
E-mails: glamberti@unisa.it (G. Lamberti), igaldi@unisa.it (I. Galdi), gtitomanlio@unisa.it (G. Titomanlio), aabarba@unisa.it (A. A. Barba), mdamore@unisa.it (M. d’Amore)
Electronic publication date: 2010 Sep 30
Print publication date: 2010 Jul-Sep
Volume: 78
Issue: 3
First page: 625
Last page: 625
Copyright: © author(s); licensee Österreichische Apotheker-Verlagsgesellschaft m. b. H., Vienna, Austria.
Copyright year: 2010
License: This is an Open Access article distributed under the terms of the Creative Commons Attribution License (http://creativecommons.org/licenses/by/3.0/), which permits unrestricted use, distribution, and reproduction in any medium, provided the original work is properly cited.

Fig. 1. Shape and hydration levels for the HPMC-TP 1:1 matrices subjected to the hydration. Snapshot of cut matrices (experimental) and superimposed simulated profiles (model) after 12 hours of hydration. The initial, non-swollen half-matrices shape is a rectangle (diameter 13 mm, semi-thickness 1.1 mm).

JOURNAL INFORMATION
==============================
NLM Title Abbreviation: Sci Pharm
Journal ID: Sci Pharm
NLM Journal ID: 0026251
Journal ID: Scientia Pharmaceutica

Scientia Pharmaceutica

ISSN: 0036-8709
EISSN: 2218-0532
Publisher: Österreichische Apotheker-Verlagsgesellschaft, m. b. H.

ARTICLE INFORMATION
==============================
DOI: 10.3797/scipharm.cespt.8.PDD37
Article ID: scipharm.2010.78.626
Subjects: Conference abstract PDD37

Influence of Concentration of Selected Cationic Surfactants on Drug Liberation

Vitková Z.
Antonyová D.
Herdová P.
Žabka M.
Oremusová J.
Faculty of Pharmacy, Commenius University, Bratislava, Slovakia
E-mail: herdova@fpharm.uniba.sk (P. Herdová)
Electronic publication date: 2010 Sep 30
Print publication date: 2010 Jul-Sep
Volume: 78
Issue: 3
First page: 626
Last page: 626
Copyright: © author(s); licensee Österreichische Apotheker-Verlagsgesellschaft m. b. H., Vienna, Austria.
Copyright year: 2010
License: This is an Open Access article distributed under the terms of the Creative Commons Attribution License (http://creativecommons.org/licenses/by/3.0/), which permits unrestricted use, distribution, and reproduction in any medium, provided the original work is properly cited.

JOURNAL INFORMATION
==============================
NLM Title Abbreviation: Sci Pharm
Journal ID: Sci Pharm
NLM Journal ID: 0026251
Journal ID: Scientia Pharmaceutica

Scientia Pharmaceutica

ISSN: 0036-8709
EISSN: 2218-0532
Publisher: Österreichische Apotheker-Verlagsgesellschaft, m. b. H.

ARTICLE INFORMATION
==============================
DOI: 10.3797/scipharm.cespt.8.PDD38
Article ID: scipharm.2010.78.627
Subjects: Conference abstract PDD38

Influence of Adjuvances on the Drug Release from Hydrogels

Oremusová J. 1
Vitková Z. 2
Herdová P. 2
1 Department of Physical Chemistry of Drugs, Faculty of Pharmacy, Commenius University, Bratislava, Slovakia
2 Department of Galenic Pharmacy, Faculty of Pharmacy, Commenius University, Bratislava, Slovakia
E-mail: herdova@fpharm.uniba.sk (P. Herdová)
Electronic publication date: 2010 Sep 30
Print publication date: 2010 Jul-Sep
Volume: 78
Issue: 3
First page: 627
Last page: 627
Copyright: © author(s); licensee Österreichische Apotheker-Verlagsgesellschaft m. b. H., Vienna, Austria.
Copyright year: 2010
License: This is an Open Access article distributed under the terms of the Creative Commons Attribution License (http://creativecommons.org/licenses/by/3.0/), which permits unrestricted use, distribution, and reproduction in any medium, provided the original work is properly cited.

JOURNAL INFORMATION
==============================
NLM Title Abbreviation: Sci Pharm
Journal ID: Sci Pharm
NLM Journal ID: 0026251
Journal ID: Scientia Pharmaceutica

Scientia Pharmaceutica

ISSN: 0036-8709
EISSN: 2218-0532
Publisher: Österreichische Apotheker-Verlagsgesellschaft, m. b. H.

ARTICLE INFORMATION
==============================
DOI: 10.3797/scipharm.cespt.8.PMS01
Article ID: scipharm.2010.78.628
Subjects: Conference abstract PMS01

Continuous API Synthesis using Heterogeneous Catalysis

Gruber-Wölfler H. 1
Radaschitz P. 1
Feenstra P. 1
Lichtenegger G. J. 1
Polo E. 2
Khinast J. G. 1
1 Institute for Process and Particle Engineering, Graz University of Technology, Graz, Austria
2 C.N.R.-ISOF Sez. Ferrara, Dip. di Chimica, Via L.Borsari 46, I-44100 Ferrara, Italy
E-mail: woelfler@tugraz.at (H. Gruber-Wölfler)
Electronic publication date: 2010 Sep 30
Print publication date: 2010 Jul-Sep
Volume: 78
Issue: 3
First page: 628
Last page: 628
Copyright: © author(s); licensee Österreichische Apotheker-Verlagsgesellschaft m. b. H., Vienna, Austria.
Copyright year: 2010
License: This is an Open Access article distributed under the terms of the Creative Commons Attribution License (http://creativecommons.org/licenses/by/3.0/), which permits unrestricted use, distribution, and reproduction in any medium, provided the original work is properly cited.

JOURNAL INFORMATION
==============================
NLM Title Abbreviation: Sci Pharm
Journal ID: Sci Pharm
NLM Journal ID: 0026251
Journal ID: Scientia Pharmaceutica

Scientia Pharmaceutica

ISSN: 0036-8709
EISSN: 2218-0532
Publisher: Österreichische Apotheker-Verlagsgesellschaft, m. b. H.

ARTICLE INFORMATION
==============================
DOI: 10.3797/scipharm.cespt.8.PMS02
Article ID: scipharm.2010.78.629
Subjects: Conference abstract PMS02

A Microreactor Strategy for the Synthesis of Tetrazoles

Gutmann B. 1
Roduit J. P. 2
Roberge D. 2
Kappe C. O. 1
1 Christian Doppler Laboratory for Microwave Chemistry and Institute of Chemistry, Karl-Franzens-University Graz, A-8010 Graz, Austria
2 Microreactor Technology, Lonza AG, CH-3930 Visp, Switzerland
E-mail: oliver.kappe@uni-graz.at (C. O. Kappe)
Electronic publication date: 2010 Sep 30
Print publication date: 2010 Jul-Sep
Volume: 78
Issue: 3
First page: 629
Last page: 629
Copyright: © author(s); licensee Österreichische Apotheker-Verlagsgesellschaft m. b. H., Vienna, Austria.
Copyright year: 2010
License: This is an Open Access article distributed under the terms of the Creative Commons Attribution License (http://creativecommons.org/licenses/by/3.0/), which permits unrestricted use, distribution, and reproduction in any medium, provided the original work is properly cited.

JOURNAL INFORMATION
==============================
NLM Title Abbreviation: Sci Pharm
Journal ID: Sci Pharm
NLM Journal ID: 0026251
Journal ID: Scientia Pharmaceutica

Scientia Pharmaceutica

ISSN: 0036-8709
EISSN: 2218-0532
Publisher: Österreichische Apotheker-Verlagsgesellschaft, m. b. H.

ARTICLE INFORMATION
==============================
DOI: 10.3797/scipharm.cespt.8.PMS03
Article ID: scipharm.2010.78.630
Subjects: Conference abstract PMS03

Continuous Flow Chemistry under High-Temperature / High-Pressure Conditions

Obermayer D. 1
Glasnov T. N. 1
Razzaq T. 1
Irfan M. 1
Damm M. 1
Kappe C. O. 1
Christian Doppler Laboratory for Microwave Chemistry and Institute of Chemistry, Karl-Franzens-University, Graz, Austria
E-mails: oliver.kappe@uni-graz.at (C. O. Kappe)
Electronic publication date: 2010 Sep 30
Print publication date: 2010 Jul-Sep
Volume: 78
Issue: 3
First page: 630
Last page: 630
Copyright: © author(s); licensee Österreichische Apotheker-Verlagsgesellschaft m. b. H., Vienna, Austria.
Copyright year: 2010
License: This is an Open Access article distributed under the terms of the Creative Commons Attribution License (http://creativecommons.org/licenses/by/3.0/), which permits unrestricted use, distribution, and reproduction in any medium, provided the original work is properly cited.

JOURNAL INFORMATION
==============================
NLM Title Abbreviation: Sci Pharm
Journal ID: Sci Pharm
NLM Journal ID: 0026251
Journal ID: Scientia Pharmaceutica

Scientia Pharmaceutica

ISSN: 0036-8709
EISSN: 2218-0532
Publisher: Österreichische Apotheker-Verlagsgesellschaft, m. b. H.

ARTICLE INFORMATION
==============================
DOI: 10.3797/scipharm.cespt.8.PMS04
Article ID: scipharm.2010.78.631
Subjects: Conference abstract PMS04

Heterogeneous Hydrogenations in Continuous Flow

Irfan M.
Glasnov T. N.
Kappe C. O.
Christian Doppler Laboratory for Microwave Chemistry (CDLMC) and Institute of Chemistry, Karl-Franzens-University Graz, Heinrichstrasse 28, A-8010 Graz, Austria
E-mail: muhammad.irfan@edu.uni-graz.at (M. Irfan)
Electronic publication date: 2010 Sep 30
Print publication date: 2010 Jul-Sep
Volume: 78
Issue: 3
First page: 631
Last page: 631
Copyright: © author(s); licensee Österreichische Apotheker-Verlagsgesellschaft m. b. H., Vienna, Austria.
Copyright year: 2010
License: This is an Open Access article distributed under the terms of the Creative Commons Attribution License (http://creativecommons.org/licenses/by/3.0/), which permits unrestricted use, distribution, and reproduction in any medium, provided the original work is properly cited.

JOURNAL INFORMATION
==============================
NLM Title Abbreviation: Sci Pharm
Journal ID: Sci Pharm
NLM Journal ID: 0026251
Journal ID: Scientia Pharmaceutica

Scientia Pharmaceutica

ISSN: 0036-8709
EISSN: 2218-0532
Publisher: Österreichische Apotheker-Verlagsgesellschaft, m. b. H.

ARTICLE INFORMATION
==============================
DOI: 10.3797/scipharm.cespt.8.PMS05
Article ID: scipharm.2010.78.632
Subjects: Conference abstract PMS05

Assessment of Powder Mixing using Positron Emission Tomography and Particle Tracking

Armstrong B. 1
Seville J. 2
Parker D. 3
1 Freeman Technology, Malvern, England
2 School of Engineering, University of Warwick, Warwick, England
3 School of Physics & Astronomy, University of Birmingham, Birmingham, England
E-mail: J.P.K.Seville@warwick.ac.uk (J. Seville)
Electronic publication date: 2010 Sep 30
Print publication date: 2010 Jul-Sep
Volume: 78
Issue: 3
First page: 632
Last page: 632
Copyright: © author(s); licensee Österreichische Apotheker-Verlagsgesellschaft m. b. H., Vienna, Austria.
Copyright year: 2010
License: This is an Open Access article distributed under the terms of the Creative Commons Attribution License (http://creativecommons.org/licenses/by/3.0/), which permits unrestricted use, distribution, and reproduction in any medium, provided the original work is properly cited.

JOURNAL INFORMATION
==============================
NLM Title Abbreviation: Sci Pharm
Journal ID: Sci Pharm
NLM Journal ID: 0026251
Journal ID: Scientia Pharmaceutica

Scientia Pharmaceutica

ISSN: 0036-8709
EISSN: 2218-0532
Publisher: Österreichische Apotheker-Verlagsgesellschaft, m. b. H.

ARTICLE INFORMATION
==============================
DOI: 10.3797/scipharm.cespt.8.PMS06
Article ID: scipharm.2010.78.633
Subjects: Conference abstract PMS06

Simulation Studies for the Effects of Particle Size and Blade Rake Angle on Particle Mixing

Siraj M. S.
Radl S.
Khinast J. G.
Institute for Process and Particle Engineering (IPPE), Graz University of Technology, Graz, Austria
E-mails: m.s.siraj@TUGraz.at (M. S. Siraj), stefan.radl@TUGraz.at (S. Radl), khinast@TUGraz.at (J. G. Khinast)
Electronic publication date: 2010 Sep 30
Print publication date: 2010 Jul-Sep
Volume: 78
Issue: 3
First page: 633
Last page: 633
Copyright: © author(s); licensee Österreichische Apotheker-Verlagsgesellschaft m. b. H., Vienna, Austria.
Copyright year: 2010
License: This is an Open Access article distributed under the terms of the Creative Commons Attribution License (http://creativecommons.org/licenses/by/3.0/), which permits unrestricted use, distribution, and reproduction in any medium, provided the original work is properly cited.

JOURNAL INFORMATION
==============================
NLM Title Abbreviation: Sci Pharm
Journal ID: Sci Pharm
NLM Journal ID: 0026251
Journal ID: Scientia Pharmaceutica

Scientia Pharmaceutica

ISSN: 0036-8709
EISSN: 2218-0532
Publisher: Österreichische Apotheker-Verlagsgesellschaft, m. b. H.

ARTICLE INFORMATION
==============================
DOI: 10.3797/scipharm.cespt.8.PMS07
Article ID: scipharm.2010.78.634
Subjects: Conference abstract PMS07

Quantitative Quasi-Simultaneous and Spatially Resolved Real-time Monitoring of Powder Mixing Processes with a Multiple NIR-Probe Setup

Balak N. 1
Scheibelhofer O. 1
Koller D. M. 1
Khinast J. G. 1 2
1 Research Center Pharmaceutical Engineering GmbH, Graz, Austria
2 Institute for Process and Particle Engineering, Graz University of Technology, Graz, Austria
E-mails: nikolaus.balak@rcpe.at (N. Balak), khinast@tugraz.at (J. G. Khinast)
Electronic publication date: 2010 Sep 30
Print publication date: 2010 Jul-Sep
Volume: 78
Issue: 3
First page: 634
Last page: 634
Copyright: © author(s); licensee Österreichische Apotheker-Verlagsgesellschaft m. b. H., Vienna, Austria.
Copyright year: 2010
License: This is an Open Access article distributed under the terms of the Creative Commons Attribution License (http://creativecommons.org/licenses/by/3.0/), which permits unrestricted use, distribution, and reproduction in any medium, provided the original work is properly cited.

JOURNAL INFORMATION
==============================
NLM Title Abbreviation: Sci Pharm
Journal ID: Sci Pharm
NLM Journal ID: 0026251
Journal ID: Scientia Pharmaceutica

Scientia Pharmaceutica

ISSN: 0036-8709
EISSN: 2218-0532
Publisher: Österreichische Apotheker-Verlagsgesellschaft, m. b. H.

ARTICLE INFORMATION
==============================
DOI: 10.3797/scipharm.cespt.8.PMS08
Article ID: scipharm.2010.78.635
Subjects: Conference abstract PMS08

Linking Rheological Key Parameters of Pharmaceutical Powders to Mixing Properties

Scheibelhofer O. 1
Balak N. 1
Koller D. M. 1
Fraser S. D. 1
Freeman T. 2
Khinast J. G. 1 3
1 Research Center Pharmaceutical Engineering GmbH, Graz, Austria
2 Freeman Technology, Worcestershire, United Kingdom
3 Institute for Process and Particle Engineering, University of Technology, Graz, Austria
E-mails: daniel.koller@rcpe.at (D. M. Koller), khinast@tugraz.at (J. G. Khinast)
Electronic publication date: 2010 Sep 30
Print publication date: 2010 Jul-Sep
Volume: 78
Issue: 3
First page: 635
Last page: 635
Copyright: © author(s); licensee Österreichische Apotheker-Verlagsgesellschaft m. b. H., Vienna, Austria.
Copyright year: 2010
License: This is an Open Access article distributed under the terms of the Creative Commons Attribution License (http://creativecommons.org/licenses/by/3.0/), which permits unrestricted use, distribution, and reproduction in any medium, provided the original work is properly cited.

JOURNAL INFORMATION
==============================
NLM Title Abbreviation: Sci Pharm
Journal ID: Sci Pharm
NLM Journal ID: 0026251
Journal ID: Scientia Pharmaceutica

Scientia Pharmaceutica

ISSN: 0036-8709
EISSN: 2218-0532
Publisher: Österreichische Apotheker-Verlagsgesellschaft, m. b. H.

ARTICLE INFORMATION
==============================
DOI: 10.3797/scipharm.cespt.8.PMS09
Article ID: scipharm.2010.78.636
Subjects: Conference abstract PMS09

Simulation of Fluid Mixing and Dissolution Processes

Hörmann T. 1
Gsöll M. 1
Hofer J. 1
Suzzi D. 1
Khinast J. G. 1 2
1 Research Center Pharmaceutical Engineering GmbH, Graz, Austria
2 Institute for Process and Particle Engineering, Graz University of Technology, Graz, Austria
E-mail: khinast@tugraz.at (J. G. Khinast)
Electronic publication date: 2010 Sep 30
Print publication date: 2010 Jul-Sep
Volume: 78
Issue: 3
First page: 636
Last page: 636
Copyright: © author(s); licensee Österreichische Apotheker-Verlagsgesellschaft m. b. H., Vienna, Austria.
Copyright year: 2010
License: This is an Open Access article distributed under the terms of the Creative Commons Attribution License (http://creativecommons.org/licenses/by/3.0/), which permits unrestricted use, distribution, and reproduction in any medium, provided the original work is properly cited.

JOURNAL INFORMATION
==============================
NLM Title Abbreviation: Sci Pharm
Journal ID: Sci Pharm
NLM Journal ID: 0026251
Journal ID: Scientia Pharmaceutica

Scientia Pharmaceutica

ISSN: 0036-8709
EISSN: 2218-0532
Publisher: Österreichische Apotheker-Verlagsgesellschaft, m. b. H.

ARTICLE INFORMATION
==============================
DOI: 10.3797/scipharm.cespt.8.PMS10
Article ID: scipharm.2010.78.637
Subjects: Conference abstract PMS10

Role of the Surface Free Energy in the Preparation of Granules and in the Selection of a Suitable Device

Korbely A.
Pintye-Hódi K.
Department of Pharmaceutical Technology, University of Szeged, Szeged, Hungary
E-mails: korbely@pharm.u-szeged.hu (A. Korbely), klara.hodi@pharm.u-szeged.hu (K. Pintye-Hódi)
Electronic publication date: 2010 Sep 30
Print publication date: 2010 Jul-Sep
Volume: 78
Issue: 3
First page: 637
Last page: 637
Copyright: © author(s); licensee Österreichische Apotheker-Verlagsgesellschaft m. b. H., Vienna, Austria.
Copyright year: 2010
License: This is an Open Access article distributed under the terms of the Creative Commons Attribution License (http://creativecommons.org/licenses/by/3.0/), which permits unrestricted use, distribution, and reproduction in any medium, provided the original work is properly cited.

JOURNAL INFORMATION
==============================
NLM Title Abbreviation: Sci Pharm
Journal ID: Sci Pharm
NLM Journal ID: 0026251
Journal ID: Scientia Pharmaceutica

Scientia Pharmaceutica

ISSN: 0036-8709
EISSN: 2218-0532
Publisher: Österreichische Apotheker-Verlagsgesellschaft, m. b. H.

ARTICLE INFORMATION
==============================
DOI: 10.3797/scipharm.cespt.8.PMS11
Article ID: scipharm.2010.78.638
Subjects: Conference abstract PMS11

Fluidized Hot Melt Granulation: Influence of Process and Formulation Parameters

Mašić I.
Stojković A.
Parojčić J.
Đurić Z.
Department of Pharmaceutical Technology, Faculty of Pharmacy, University of Belgrade, Serbia
E-mail: ivana.masic@pharmacy.bg.ac.rs (I. Mašić)
Electronic publication date: 2010 Sep 30
Print publication date: 2010 Jul-Sep
Volume: 78
Issue: 3
First page: 638
Last page: 638
Copyright: © author(s); licensee Österreichische Apotheker-Verlagsgesellschaft m. b. H., Vienna, Austria.
Copyright year: 2010
License: This is an Open Access article distributed under the terms of the Creative Commons Attribution License (http://creativecommons.org/licenses/by/3.0/), which permits unrestricted use, distribution, and reproduction in any medium, provided the original work is properly cited.

JOURNAL INFORMATION
==============================
NLM Title Abbreviation: Sci Pharm
Journal ID: Sci Pharm
NLM Journal ID: 0026251
Journal ID: Scientia Pharmaceutica

Scientia Pharmaceutica

ISSN: 0036-8709
EISSN: 2218-0532
Publisher: Österreichische Apotheker-Verlagsgesellschaft, m. b. H.

ARTICLE INFORMATION
==============================
DOI: 10.3797/scipharm.cespt.8.PMS12
Article ID: scipharm.2010.78.639
Subjects: Conference abstract PMS12

Continuous Pelletization – Effect of Process Parameters

Muehlenfeld C. 1
Steiner R. 2
Thommes M. 1
1 Institute of Pharmaceutics and Biopharmaceutics, Heinrich-Heine-University, Duesseldorf, Germany
2 Leistritz Extrusionstechnik GmbH, Nuremberg, Germany
E-mail: christian.muehlenfeld@uni-duesseldorf.de (C. Muehlenfeld)
Electronic publication date: 2010 Sep 30
Print publication date: 2010 Jul-Sep
Volume: 78
Issue: 3
First page: 639
Last page: 639
Copyright: © author(s); licensee Österreichische Apotheker-Verlagsgesellschaft m. b. H., Vienna, Austria.
Copyright year: 2010
License: This is an Open Access article distributed under the terms of the Creative Commons Attribution License (http://creativecommons.org/licenses/by/3.0/), which permits unrestricted use, distribution, and reproduction in any medium, provided the original work is properly cited.

JOURNAL INFORMATION
==============================
NLM Title Abbreviation: Sci Pharm
Journal ID: Sci Pharm
NLM Journal ID: 0026251
Journal ID: Scientia Pharmaceutica

Scientia Pharmaceutica

ISSN: 0036-8709
EISSN: 2218-0532
Publisher: Österreichische Apotheker-Verlagsgesellschaft, m. b. H.

ARTICLE INFORMATION
==============================
DOI: 10.3797/scipharm.cespt.8.PMS13
Article ID: scipharm.2010.78.640
Subjects: Conference abstract PMS13

New Insights into the Pelletization Mechanism by Extrusion/Spheronization

Koester M.
Thommes M.
Institute of Pharmaceutics and Biopharmaceutics, Heinrich-Heine-University, Duesseldorf, Germany
E-mail: martin.koester@uni-duesseldorf.de (M. Koester)
Electronic publication date: 2010 Sep 30
Print publication date: 2010 Jul-Sep
Volume: 78
Issue: 3
First page: 640
Last page: 640
Copyright: © author(s); licensee Österreichische Apotheker-Verlagsgesellschaft m. b. H., Vienna, Austria.
Copyright year: 2010
License: This is an Open Access article distributed under the terms of the Creative Commons Attribution License (http://creativecommons.org/licenses/by/3.0/), which permits unrestricted use, distribution, and reproduction in any medium, provided the original work is properly cited.

Fig. 1. Images of samples taken during spheronization after different for a pure MCC formulation

JOURNAL INFORMATION
==============================
NLM Title Abbreviation: Sci Pharm
Journal ID: Sci Pharm
NLM Journal ID: 0026251
Journal ID: Scientia Pharmaceutica

Scientia Pharmaceutica

ISSN: 0036-8709
EISSN: 2218-0532
Publisher: Österreichische Apotheker-Verlagsgesellschaft, m. b. H.

ARTICLE INFORMATION
==============================
DOI: 10.3797/scipharm.cespt.8.PMS14
Article ID: scipharm.2010.78.641
Subjects: Conference abstract PMS14

Study of the Uniformity of the Coating Thickness of Pellets Coated by Wurster and Swirl Flow Generator Equipped Coater

Luštrik M. 1 2
Dreu R. 1
Srčič S. 1
1 Faculty of Pharmacy, University of Ljubjlana, Ljubljana, Slovenia
2 Brinox d.o.o., Sora 21, 1215 Mevode, Slovenia
E-mail: matevz.lustrik@ffa.uni-lj.si (M. Luštrik)
Electronic publication date: 2010 Sep 30
Print publication date: 2010 Jul-Sep
Volume: 78
Issue: 3
First page: 641
Last page: 641
Copyright: © author(s); licensee Österreichische Apotheker-Verlagsgesellschaft m. b. H., Vienna, Austria.
Copyright year: 2010
License: This is an Open Access article distributed under the terms of the Creative Commons Attribution License (http://creativecommons.org/licenses/by/3.0/), which permits unrestricted use, distribution, and reproduction in any medium, provided the original work is properly cited.

JOURNAL INFORMATION
==============================
NLM Title Abbreviation: Sci Pharm
Journal ID: Sci Pharm
NLM Journal ID: 0026251
Journal ID: Scientia Pharmaceutica

Scientia Pharmaceutica

ISSN: 0036-8709
EISSN: 2218-0532
Publisher: Österreichische Apotheker-Verlagsgesellschaft, m. b. H.

ARTICLE INFORMATION
==============================
DOI: 10.3797/scipharm.cespt.8.PMS15
Article ID: scipharm.2010.78.642
Subjects: Conference abstract PMS15

Study of the Recrystallization in Coated Pellets

Regdon G. Jr. 1
Nikowitz K. 1
Pintye-Hódi K. 1
Griesser U. J. 2
1 Department of Pharmaceutical Technology, University of Szeged, Szeged, Hungary
2 Institute of Pharmacy, University of Innsbruck, Innsbruck, Austria
E-mail: geza.regdon@pharm.u-szeged.hu (G. Regdon Jr.)
Electronic publication date: 2010 Sep 30
Print publication date: 2010 Jul-Sep
Volume: 78
Issue: 3
First page: 642
Last page: 642
Copyright: © author(s); licensee Österreichische Apotheker-Verlagsgesellschaft m. b. H., Vienna, Austria.
Copyright year: 2010
License: This is an Open Access article distributed under the terms of the Creative Commons Attribution License (http://creativecommons.org/licenses/by/3.0/), which permits unrestricted use, distribution, and reproduction in any medium, provided the original work is properly cited.

JOURNAL INFORMATION
==============================
NLM Title Abbreviation: Sci Pharm
Journal ID: Sci Pharm
NLM Journal ID: 0026251
Journal ID: Scientia Pharmaceutica

Scientia Pharmaceutica

ISSN: 0036-8709
EISSN: 2218-0532
Publisher: Österreichische Apotheker-Verlagsgesellschaft, m. b. H.

ARTICLE INFORMATION
==============================
DOI: 10.3797/scipharm.cespt.8.PMS16
Article ID: scipharm.2010.78.643
Subjects: Conference abstract PMS16

Novel Modeling Technique for Simulations of Turbulent Bubbly Flows with Coalescence and Breakup

Sungkorn R. 1
Derksen J. J. 2
Khinast J. G. 1
1 Institute for Process and Particle Engineering, Grz University of Technology, Graz, Austria
2 Chemical and Material Engineering Department, Univeristy of Alberta, Edmonton, Alberta, Canada
E-mail: r.sungkorn@tugraz.at (R. Sungkorn)
Electronic publication date: 2010 Sep 30
Print publication date: 2010 Jul-Sep
Volume: 78
Issue: 3
First page: 643
Last page: 643
Copyright: © author(s); licensee Österreichische Apotheker-Verlagsgesellschaft m. b. H., Vienna, Austria.
Copyright year: 2010
License: This is an Open Access article distributed under the terms of the Creative Commons Attribution License (http://creativecommons.org/licenses/by/3.0/), which permits unrestricted use, distribution, and reproduction in any medium, provided the original work is properly cited.

JOURNAL INFORMATION
==============================
NLM Title Abbreviation: Sci Pharm
Journal ID: Sci Pharm
NLM Journal ID: 0026251
Journal ID: Scientia Pharmaceutica

Scientia Pharmaceutica

ISSN: 0036-8709
EISSN: 2218-0532
Publisher: Österreichische Apotheker-Verlagsgesellschaft, m. b. H.

ARTICLE INFORMATION
==============================
DOI: 10.3797/scipharm.cespt.8.PMS17
Article ID: scipharm.2010.78.644
Subjects: Conference abstract PMS17

Large-Scale GPU Simulations of Granular Flows and Fluidized Beds: An LBM-DEM Coupling Approach

Radeke C. 1
Radl S. 2
Sungkorn R. 2
Suzzi D. 1
Khinast J. G. 1 2
1 Research Center Pharmaceutical Engineering (RCPE), Graz, Austria
2 Institute for Process and Particle Engineering (IPPT), Graz University of Technology, Graz, Austria
E-mails: charles.radeke@rcpe.at (C. Radeke), khinast@tugraz.at (J. G. Khinast)
Electronic publication date: 2010 Sep 30
Print publication date: 2010 Jul-Sep
Volume: 78
Issue: 3
First page: 644
Last page: 644
Copyright: © author(s); licensee Österreichische Apotheker-Verlagsgesellschaft m. b. H., Vienna, Austria.
Copyright year: 2010
License: This is an Open Access article distributed under the terms of the Creative Commons Attribution License (http://creativecommons.org/licenses/by/3.0/), which permits unrestricted use, distribution, and reproduction in any medium, provided the original work is properly cited.

JOURNAL INFORMATION
==============================
NLM Title Abbreviation: Sci Pharm
Journal ID: Sci Pharm
NLM Journal ID: 0026251
Journal ID: Scientia Pharmaceutica

Scientia Pharmaceutica

ISSN: 0036-8709
EISSN: 2218-0532
Publisher: Österreichische Apotheker-Verlagsgesellschaft, m. b. H.

ARTICLE INFORMATION
==============================
DOI: 10.3797/scipharm.cespt.8.PMS18
Article ID: scipharm.2010.78.645
Subjects: Conference abstract PMS18

Simulation of Particles with Different Coefficient of Restitution in Fluid Bed Coater

Šibanc R.
Dreu R.
Srčič S.
Faculty of Pharmacy, University of Ljubljana, Ljubljana, Slovenia
E-mail: rok.sibanc@ffa.uni-lj.si (R. Šibanc)
Electronic publication date: 2010 Sep 30
Print publication date: 2010 Jul-Sep
Volume: 78
Issue: 3
First page: 645
Last page: 645
Copyright: © author(s); licensee Österreichische Apotheker-Verlagsgesellschaft m. b. H., Vienna, Austria.
Copyright year: 2010
License: This is an Open Access article distributed under the terms of the Creative Commons Attribution License (http://creativecommons.org/licenses/by/3.0/), which permits unrestricted use, distribution, and reproduction in any medium, provided the original work is properly cited.

JOURNAL INFORMATION
==============================
NLM Title Abbreviation: Sci Pharm
Journal ID: Sci Pharm
NLM Journal ID: 0026251
Journal ID: Scientia Pharmaceutica

Scientia Pharmaceutica

ISSN: 0036-8709
EISSN: 2218-0532
Publisher: Österreichische Apotheker-Verlagsgesellschaft, m. b. H.

ARTICLE INFORMATION
==============================
DOI: 10.3797/scipharm.cespt.8.PMS19
Article ID: scipharm.2010.78.646
Subjects: Conference abstract PMS19

An Investigation into the Use of Polyox® as the Binding and Coating Agent in Fluidized-Bed Apparatus

Stojkovic A.
Masic I.
Parojcic J.
Djuric Z.
Department of Pharmaceutical Technology, Faculty of Pharmacy, University of Belgrade, Serbia
E-mail: sandric26@gmail.com (A. Stojkovic)
Electronic publication date: 2010 Sep 30
Print publication date: 2010 Jul-Sep
Volume: 78
Issue: 3
First page: 646
Last page: 646
Copyright: © author(s); licensee Österreichische Apotheker-Verlagsgesellschaft m. b. H., Vienna, Austria.
Copyright year: 2010
License: This is an Open Access article distributed under the terms of the Creative Commons Attribution License (http://creativecommons.org/licenses/by/3.0/), which permits unrestricted use, distribution, and reproduction in any medium, provided the original work is properly cited.

This work was supported by the Ministry of Science and Technological Development, Republic of Serbia project TR23015.

JOURNAL INFORMATION
==============================
NLM Title Abbreviation: Sci Pharm
Journal ID: Sci Pharm
NLM Journal ID: 0026251
Journal ID: Scientia Pharmaceutica

Scientia Pharmaceutica

ISSN: 0036-8709
EISSN: 2218-0532
Publisher: Österreichische Apotheker-Verlagsgesellschaft, m. b. H.

ARTICLE INFORMATION
==============================
DOI: 10.3797/scipharm.cespt.8.PMS20
Article ID: scipharm.2010.78.647
Subjects: Conference abstract PMS20

Melt Granulation in Fluidised Bed: Screening of the Process Variables by Factorial Design

Calogerá G. 1
Passerini N. 1
Albertini B. 1
Hasa D. 2
Voinovich D. 2
1 Department of Pharmaceutical Sciences, University of Bologna, Bologna, Italy
2 Department of Pharmaceutical Sciences, University of Trieste, Trieste, Italy
E-mails: nadia.passerini@unibo.it (N. Passerini), vojnovic@units.it (D. Voinovich)
Electronic publication date: 2010 Sep 30
Print publication date: 2010 Jul-Sep
Volume: 78
Issue: 3
First page: 647
Last page: 647
Copyright: © author(s); licensee Österreichische Apotheker-Verlagsgesellschaft m. b. H., Vienna, Austria.
Copyright year: 2010
License: This is an Open Access article distributed under the terms of the Creative Commons Attribution License (http://creativecommons.org/licenses/by/3.0/), which permits unrestricted use, distribution, and reproduction in any medium, provided the original work is properly cited.

JOURNAL INFORMATION
==============================
NLM Title Abbreviation: Sci Pharm
Journal ID: Sci Pharm
NLM Journal ID: 0026251
Journal ID: Scientia Pharmaceutica

Scientia Pharmaceutica

ISSN: 0036-8709
EISSN: 2218-0532
Publisher: Österreichische Apotheker-Verlagsgesellschaft, m. b. H.

ARTICLE INFORMATION
==============================
DOI: 10.3797/scipharm.cespt.8.PMS21
Article ID: scipharm.2010.78.648
Subjects: Conference abstract PMS21

A modification of Pr equation for Measurement of Tablet’s Compactibility

Ilić I. 1
Kása P. Jr 2
Dreu R. 1
Pintye-Hódi K. 2
Srčič S. 1
1 Faculty of Pharmacy, University of Ljubljana, Ljubljana, Slovenia
2 Faculty of Pharmacy, University of Szeged, Szeged, Hungary
E-mail: ilija.ilic@ffa.uni-lj.si (I. Ilic)
Electronic publication date: 2010 Sep 30
Print publication date: 2010 Jul-Sep
Volume: 78
Issue: 3
First page: 648
Last page: 648
Copyright: © author(s); licensee Österreichische Apotheker-Verlagsgesellschaft m. b. H., Vienna, Austria.
Copyright year: 2010
License: This is an Open Access article distributed under the terms of the Creative Commons Attribution License (http://creativecommons.org/licenses/by/3.0/), which permits unrestricted use, distribution, and reproduction in any medium, provided the original work is properly cited.

JOURNAL INFORMATION
==============================
NLM Title Abbreviation: Sci Pharm
Journal ID: Sci Pharm
NLM Journal ID: 0026251
Journal ID: Scientia Pharmaceutica

Scientia Pharmaceutica

ISSN: 0036-8709
EISSN: 2218-0532
Publisher: Österreichische Apotheker-Verlagsgesellschaft, m. b. H.

ARTICLE INFORMATION
==============================
DOI: 10.3797/scipharm.cespt.8.PMS22
Article ID: scipharm.2010.78.649
Subjects: Conference abstract PMS22

Computer-Aided Design of Pharmaceutical Tablet Formulations

Kimber J. A. 1
Kazarian S. G. 1
Štěpánek F. 1 2
1 Department of Chemical Engineering, Imperial College London, London, United Kingdom
2 Institue of Chemical Technology, Prague, Czech Republic
E-mails: james.kimber03@imperial.ac.uk (J. A. Kimber), Frantisek.Stepanek@vscht.cz (F. Štěpánek)
Electronic publication date: 2010 Sep 30
Print publication date: 2010 Jul-Sep
Volume: 78
Issue: 3
First page: 649
Last page: 649
Copyright: © author(s); licensee Österreichische Apotheker-Verlagsgesellschaft m. b. H., Vienna, Austria.
Copyright year: 2010
License: This is an Open Access article distributed under the terms of the Creative Commons Attribution License (http://creativecommons.org/licenses/by/3.0/), which permits unrestricted use, distribution, and reproduction in any medium, provided the original work is properly cited.

JOURNAL INFORMATION
==============================
NLM Title Abbreviation: Sci Pharm
Journal ID: Sci Pharm
NLM Journal ID: 0026251
Journal ID: Scientia Pharmaceutica

Scientia Pharmaceutica

ISSN: 0036-8709
EISSN: 2218-0532
Publisher: Österreichische Apotheker-Verlagsgesellschaft, m. b. H.

ARTICLE INFORMATION
==============================
DOI: 10.3797/scipharm.cespt.8.PMS23
Article ID: scipharm.2010.78.650
Subjects: Conference abstract PMS23

The “Drill & Fill” Method

Philippe C. 1 2
Haeusler D. 1 2
Mien L.-K. 1 2
Kletter K. 1
Dudczak R. 1
Wadsak W. 1 3
Viernstein H. 2
Mitterhauser M. 1 2 4
1 Dept of Nuclear Medicine, Medical University of Vienna, Austria
2 Dept of Pharmaceutical Technology and Biopharmaceutics, University of Vienna, Austria
3 Dept of Inorganic Chemistry, University of Vienna, Austria
4 Hospital Pharmacy of the General Hospital of Vienna, Austria
E-Mails: cecile.philippe@meduniwien.ac.at (C. Philippe), helmut.viernstein@univie.ac.at (H. Viernstein), markus.mitterhauser@meduniwien.ac.at (M. Mitterhauser)
Electronic publication date: 2010 Sep 30
Print publication date: 2010 Jul-Sep
Volume: 78
Issue: 3
First page: 650
Last page: 650
Copyright: © author(s); licensee Österreichische Apotheker-Verlagsgesellschaft m. b. H., Vienna, Austria.
Copyright year: 2010
License: This is an Open Access article distributed under the terms of the Creative Commons Attribution License (http://creativecommons.org/licenses/by/3.0/), which permits unrestricted use, distribution, and reproduction in any medium, provided the original work is properly cited.

JOURNAL INFORMATION
==============================
NLM Title Abbreviation: Sci Pharm
Journal ID: Sci Pharm
NLM Journal ID: 0026251
Journal ID: Scientia Pharmaceutica

Scientia Pharmaceutica

ISSN: 0036-8709
EISSN: 2218-0532
Publisher: Österreichische Apotheker-Verlagsgesellschaft, m. b. H.

ARTICLE INFORMATION
==============================
DOI: 10.3797/scipharm.cespt.8.PMS24
Article ID: scipharm.2010.78.651
Subjects: Conference abstract PMS24

Tracking of Physicochemical Properties in Free Films Containing an API

Bölcskei É. 1
Regdon G. 1
Süvegh K. 2
Marek T. 3
Pintye-hódi K. 1
1 Department of Pharmaceutical Technology, University of Szeged, Szeged, Hungary
2 Department of Nuclear Chemistry, Eötvös Loránd University, Budapest, Hungary
3 Hungarian Academy of Sciences, Research Group for Nuclear Techniques in Structural Chemistry, Eötvös Loránd University, Budapest, Hungary
E-mail: bolcskei@pharm.u-szeged.hu (É. Bölcskei)
Electronic publication date: 2010 Sep 30
Print publication date: 2010 Jul-Sep
Volume: 78
Issue: 3
First page: 651
Last page: 651
Copyright: © author(s); licensee Österreichische Apotheker-Verlagsgesellschaft m. b. H., Vienna, Austria.
Copyright year: 2010
License: This is an Open Access article distributed under the terms of the Creative Commons Attribution License (http://creativecommons.org/licenses/by/3.0/), which permits unrestricted use, distribution, and reproduction in any medium, provided the original work is properly cited.

JOURNAL INFORMATION
==============================
NLM Title Abbreviation: Sci Pharm
Journal ID: Sci Pharm
NLM Journal ID: 0026251
Journal ID: Scientia Pharmaceutica

Scientia Pharmaceutica

ISSN: 0036-8709
EISSN: 2218-0532
Publisher: Österreichische Apotheker-Verlagsgesellschaft, m. b. H.

ARTICLE INFORMATION
==============================
DOI: 10.3797/scipharm.cespt.8.PMS25
Article ID: scipharm.2010.78.652
Subjects: Conference abstract PMS25

Impact of Different Drying Processes on the Cellular Activity of Enterococcus faecium M74

Stummer S.
Rabenreither M. C
Viernstein H.
Wirth M.
Toegel S.
Salar-behzadi S.
Department of Pharmaceutical Technology and Biopharmaceutics, University of Vienna, Althanstraße 14, 1090 Vienna, Austria
E-mails: stefanie.stummer@univie.ac.at (S. Stummer), sharareh.salar-behzadi@univie.ac.at (S. Salar-Behzadi)
Electronic publication date: 2010 Sep 30
Print publication date: 2010 Jul-Sep
Volume: 78
Issue: 3
First page: 652
Last page: 652
Copyright: © author(s); licensee Österreichische Apotheker-Verlagsgesellschaft m. b. H., Vienna, Austria.
Copyright year: 2010
License: This is an Open Access article distributed under the terms of the Creative Commons Attribution License (http://creativecommons.org/licenses/by/3.0/), which permits unrestricted use, distribution, and reproduction in any medium, provided the original work is properly cited.

JOURNAL INFORMATION
==============================
NLM Title Abbreviation: Sci Pharm
Journal ID: Sci Pharm
NLM Journal ID: 0026251
Journal ID: Scientia Pharmaceutica

Scientia Pharmaceutica

ISSN: 0036-8709
EISSN: 2218-0532
Publisher: Österreichische Apotheker-Verlagsgesellschaft, m. b. H.

ARTICLE INFORMATION
==============================
DOI: 10.3797/scipharm.cespt.8.PMS26
Article ID: scipharm.2010.78.653
Subjects: Conference abstract PMS26

Can Collapse Freeze Drying Provide High Density Protein Sugar Particles for Ballistic Powder Injection?

Kis E. E.
Winter G.
Myschik J.
Department of Pharmacy, Ludwig-Maximilians-University, Munich, Germany
E-mail: julia.myschik@cup.uni-muenchen.de (J. Myschik)
Electronic publication date: 2010 Sep 30
Print publication date: 2010 Jul-Sep
Volume: 78
Issue: 3
First page: 653
Last page: 653
Copyright: © author(s); licensee Österreichische Apotheker-Verlagsgesellschaft m. b. H., Vienna, Austria.
Copyright year: 2010
License: This is an Open Access article distributed under the terms of the Creative Commons Attribution License (http://creativecommons.org/licenses/by/3.0/), which permits unrestricted use, distribution, and reproduction in any medium, provided the original work is properly cited.

JOURNAL INFORMATION
==============================
NLM Title Abbreviation: Sci Pharm
Journal ID: Sci Pharm
NLM Journal ID: 0026251
Journal ID: Scientia Pharmaceutica

Scientia Pharmaceutica

ISSN: 0036-8709
EISSN: 2218-0532
Publisher: Österreichische Apotheker-Verlagsgesellschaft, m. b. H.

ARTICLE INFORMATION
==============================
DOI: 10.3797/scipharm.cespt.8.PMS27
Article ID: scipharm.2010.78.654
Subjects: Conference abstract PMS27

Hyperbranched Poly(ester amides) – New Solubilization Enhancers for Poorly Water-Soluble Slimepiride

Reven S. 1
Grdadolnik J. 2
Kristl J. 3
Žagar E. 2
1 Lek farmacevtska družba d.d., Razvoj in raziskave, Verovškova 57, Ljubljana, Slovenija
2 National Institute of Chemistry, Hajdrihova 19, Ljubljana, Slovenia
3 University of Ljubljana, Faculty of Pharmacy, Aškerčeva 7, 1000 Ljubljana, Slovenia
E-mails: sebastjan.reven@sandoz.com (S. Reven), ema.zagar@ki.si (E. Žagar)
Electronic publication date: 2010 Sep 30
Print publication date: 2010 Jul-Sep
Volume: 78
Issue: 3
First page: 654
Last page: 654
Copyright: © author(s); licensee Österreichische Apotheker-Verlagsgesellschaft m. b. H., Vienna, Austria.
Copyright year: 2010
License: This is an Open Access article distributed under the terms of the Creative Commons Attribution License (http://creativecommons.org/licenses/by/3.0/), which permits unrestricted use, distribution, and reproduction in any medium, provided the original work is properly cited.

JOURNAL INFORMATION
==============================
NLM Title Abbreviation: Sci Pharm
Journal ID: Sci Pharm
NLM Journal ID: 0026251
Journal ID: Scientia Pharmaceutica

Scientia Pharmaceutica

ISSN: 0036-8709
EISSN: 2218-0532
Publisher: Österreichische Apotheker-Verlagsgesellschaft, m. b. H.

ARTICLE INFORMATION
==============================
DOI: 10.3797/scipharm.cespt.8.PMS28
Article ID: scipharm.2010.78.655
Subjects: Conference abstract PMS28

Novel Approach in Formulation of Low Soluble Drugs: Gliclazide as Model Substance

Jović M.
Jezdić M.
Petrović J.
Ibrić S.
Institute of Pharmaceutical Technology, Faculty of Pharmacy, University of Belgrade, Belgrade, Serbia
E-mail: jpetrovic@pharmacy.bg.ac.rs (J. Petrović)
Electronic publication date: 2010 Sep 30
Print publication date: 2010 Jul-Sep
Volume: 78
Issue: 3
First page: 655
Last page: 655
Copyright: © author(s); licensee Österreichische Apotheker-Verlagsgesellschaft m. b. H., Vienna, Austria.
Copyright year: 2010
License: This is an Open Access article distributed under the terms of the Creative Commons Attribution License (http://creativecommons.org/licenses/by/3.0/), which permits unrestricted use, distribution, and reproduction in any medium, provided the original work is properly cited.

JOURNAL INFORMATION
==============================
NLM Title Abbreviation: Sci Pharm
Journal ID: Sci Pharm
NLM Journal ID: 0026251
Journal ID: Scientia Pharmaceutica

Scientia Pharmaceutica

ISSN: 0036-8709
EISSN: 2218-0532
Publisher: Österreichische Apotheker-Verlagsgesellschaft, m. b. H.

ARTICLE INFORMATION
==============================
DOI: 10.3797/scipharm.cespt.8.PMS29
Article ID: scipharm.2010.78.656
Subjects: Conference abstract PMS29

Preparation and Evaluation of Solid Oral Lipid-Based Diazepam Preparations

Radoman N.
Radivojša M.
petrović J.
Ibrić S.
Institute of Pharmaceutical Technology, Faculty of Pharmacy, University of Belgrade, Belgrade, Serbia
E-mails: rad_nikola@yahoo.com (N. Radoman), maja_radivojsa@yahoo.com (M. Radivojša), jpetrovic@pharmacy.bg.ac.rs (J. Petrović)
Electronic publication date: 2010 Sep 30
Print publication date: 2010 Jul-Sep
Volume: 78
Issue: 3
First page: 656
Last page: 656
Copyright: © author(s); licensee Österreichische Apotheker-Verlagsgesellschaft m. b. H., Vienna, Austria.
Copyright year: 2010
License: This is an Open Access article distributed under the terms of the Creative Commons Attribution License (http://creativecommons.org/licenses/by/3.0/), which permits unrestricted use, distribution, and reproduction in any medium, provided the original work is properly cited.

JOURNAL INFORMATION
==============================
NLM Title Abbreviation: Sci Pharm
Journal ID: Sci Pharm
NLM Journal ID: 0026251
Journal ID: Scientia Pharmaceutica

Scientia Pharmaceutica

ISSN: 0036-8709
EISSN: 2218-0532
Publisher: Österreichische Apotheker-Verlagsgesellschaft, m. b. H.

ARTICLE INFORMATION
==============================
DOI: 10.3797/scipharm.cespt.8PMS30
Article ID: scipharm.2010.78.657
Subjects: Conference abstract PMS30

Physical Stability of Carvedilol in Solid Dispersions with Porous Silica

Kovačič B. 1 2
Planinšek O. 2
Vrečer F. 1 2
1 Krka d.d., Šmarješka cesta 6, SI-8501 Novo mesto, Slovenia
2 University of Ljubljana, Faculty of Pharmacy, Aškerčeva 7, SI-1000 Ljubljana, Slovenia
E-mail: borut.kovacic@ffa.uni-lj.si (B. Kovačič)
Electronic publication date: 2010 Sep 30
Print publication date: 2010 Jul-Sep
Volume: 78
Issue: 3
First page: 657
Last page: 657
Copyright: © author(s); licensee Österreichische Apotheker-Verlagsgesellschaft m. b. H., Vienna, Austria.
Copyright year: 2010
License: This is an Open Access article distributed under the terms of the Creative Commons Attribution License (http://creativecommons.org/licenses/by/3.0/), which permits unrestricted use, distribution, and reproduction in any medium, provided the original work is properly cited.

The authors acknowledge to KRKA, d.d., Novo mesto for support in the study. Present work was partly financed by the European Union, European Social Fund.

JOURNAL INFORMATION
==============================
NLM Title Abbreviation: Sci Pharm
Journal ID: Sci Pharm
NLM Journal ID: 0026251
Journal ID: Scientia Pharmaceutica

Scientia Pharmaceutica

ISSN: 0036-8709
EISSN: 2218-0532
Publisher: Österreichische Apotheker-Verlagsgesellschaft, m. b. H.

ARTICLE INFORMATION
==============================
DOI: 10.3797/scipharm.cespt.8.PMS31
Article ID: scipharm.2010.78.658
Subjects: Conference abstract PMS31

Taste Masking Technology: Recent Developments and Approaches

Gluhak A. Tomljenović 1
Simčić I. 1
Matijašević G. 2
1 PLIVA Croatia Ltd., Zagreb, Croatia
2 Faculty of Chemical Engineering and Technology, University of Zagreb, Croatia
E-mail: anamarija.tomljenovic-gluhak@pliva.com (A. Tomljenović Gluhak)
Electronic publication date: 2010 Sep 30
Print publication date: 2010 Jul-Sep
Volume: 78
Issue: 3
First page: 658
Last page: 658
Copyright: © author(s); licensee Österreichische Apotheker-Verlagsgesellschaft m. b. H., Vienna, Austria.
Copyright year: 2010
License: This is an Open Access article distributed under the terms of the Creative Commons Attribution License (http://creativecommons.org/licenses/by/3.0/), which permits unrestricted use, distribution, and reproduction in any medium, provided the original work is properly cited.

JOURNAL INFORMATION
==============================
NLM Title Abbreviation: Sci Pharm
Journal ID: Sci Pharm
NLM Journal ID: 0026251
Journal ID: Scientia Pharmaceutica

Scientia Pharmaceutica

ISSN: 0036-8709
EISSN: 2218-0532
Publisher: Österreichische Apotheker-Verlagsgesellschaft, m. b. H.

ARTICLE INFORMATION
==============================
DOI: 10.3797/scipharm.cespt.8.PMS32
Article ID: scipharm.2010.78.659
Subjects: Conference abstract PMS32

Examination of High Pressure Micro Systems by μPIV and CFD Simulations Regarding Abrasion, Particle Deposition and Stress Fields

Gothsch T. 1
Beinert S. 1
Finke J. H. 2
Lesche C. 3
Büttgenbach S. 3
Müller-Goymann C. 2
Kwade A. 1
1 Institute for Particle Technology, Technische Universität Braunschweig, Braunschweig, Germany
2 Institute for Pharmaceutical Technology, Technische Universität Braunschweig, Braunschweig, Germany
3 Institute for Microtechnology, Technische Universität Braunschweig, Braunschweig, Germany
E-mail: t.gothsch@tu-bs.de (T. Gothsch)
Electronic publication date: 2010 Sep 30
Print publication date: 2010 Jul-Sep
Volume: 78
Issue: 3
First page: 659
Last page: 659
Copyright: © author(s); licensee Österreichische Apotheker-Verlagsgesellschaft m. b. H., Vienna, Austria.
Copyright year: 2010
License: This is an Open Access article distributed under the terms of the Creative Commons Attribution License (http://creativecommons.org/licenses/by/3.0/), which permits unrestricted use, distribution, and reproduction in any medium, provided the original work is properly cited.

The authors gratefully acknowledge the DFG for financial support within the DFG research group 856 “Mikrosysteme für partikuläre Life-Science-Produkte“ (mikroPART).

JOURNAL INFORMATION
==============================
NLM Title Abbreviation: Sci Pharm
Journal ID: Sci Pharm
NLM Journal ID: 0026251
Journal ID: Scientia Pharmaceutica

Scientia Pharmaceutica

ISSN: 0036-8709
EISSN: 2218-0532
Publisher: Österreichische Apotheker-Verlagsgesellschaft, m. b. H.

ARTICLE INFORMATION
==============================
DOI: 10.3797/scipharm.cespt.8.PMS33
Article ID: scipharm.2010.78.660
Subjects: Conference abstract PMS33

Oxazoline-Based Hydro-, Amphi- and Lipogels from Microwave-Assisted Synthesis

Wiesbrock F. 1
Hecke A. 2
Wirnsberger B. 1
Kelly A. M. 2
Stelzer F. 1
1 Graz University of Technology, Institute for Chemistry and Technology of Materials, Stremayrgasse 16, AT-8010 Graz, Austria
2 Polymer Competence Center Leoben GmbH (PCCL), Roseggerstrasse 12, AT-8700 Leoben, Austria
E-mail: f.wiesbrock@tugraz.at (F.Wiesbrock)
Electronic publication date: 2010 Sep 30
Print publication date: 2010 Jul-Sep
Volume: 78
Issue: 3
First page: 660
Last page: 660
Copyright: © author(s); licensee Österreichische Apotheker-Verlagsgesellschaft m. b. H., Vienna, Austria.
Copyright year: 2010
License: This is an Open Access article distributed under the terms of the Creative Commons Attribution License (http://creativecommons.org/licenses/by/3.0/), which permits unrestricted use, distribution, and reproduction in any medium, provided the original work is properly cited.

JOURNAL INFORMATION
==============================
NLM Title Abbreviation: Sci Pharm
Journal ID: Sci Pharm
NLM Journal ID: 0026251
Journal ID: Scientia Pharmaceutica

Scientia Pharmaceutica

ISSN: 0036-8709
EISSN: 2218-0532
Publisher: Österreichische Apotheker-Verlagsgesellschaft, m. b. H.

ARTICLE INFORMATION
==============================
DOI: 10.3797/scipharm.cespt.8.PMS34
Article ID: scipharm.2010.78.661
Subjects: Conference abstract PMS34

Experimental Study of Filter Cake Cracking During Deliquoring

Barua A. 1
Eagles W. 2
Giorgio G. 2
Ricard F. 2
Stepanek F. 1
1 Department of Chemical Engineering, Imperial College London, South Kensington, SW7 2AZ, United Kingdom
2 GlaxoSmithKline Ltd., Gunnels Wood Road, Stevenage, Hertfordshire, SG1 2NY, United Kingdom
E-mail: ashok.barua08@imperial.ac.uk (A. Barua)
Electronic publication date: 2010 Sep 30
Print publication date: 2010 Jul-Sep
Volume: 78
Issue: 3
First page: 661
Last page: 661
Copyright: © author(s); licensee Österreichische Apotheker-Verlagsgesellschaft m. b. H., Vienna, Austria.
Copyright year: 2010
License: This is an Open Access article distributed under the terms of the Creative Commons Attribution License (http://creativecommons.org/licenses/by/3.0/), which permits unrestricted use, distribution, and reproduction in any medium, provided the original work is properly cited.

JOURNAL INFORMATION
==============================
NLM Title Abbreviation: Sci Pharm
Journal ID: Sci Pharm
NLM Journal ID: 0026251
Journal ID: Scientia Pharmaceutica

Scientia Pharmaceutica

ISSN: 0036-8709
EISSN: 2218-0532
Publisher: Österreichische Apotheker-Verlagsgesellschaft, m. b. H.

ARTICLE INFORMATION
==============================
DOI: 10.3797/scipharm.cespt.8.PMS35
Article ID: scipharm.2010.78.662
Subjects: Conference abstract PMS35

Impact of Tablets Hardness on Dissolution Rate of Metoprolol Tartrate 100 mg Tablets

Vehabović M.
Pilipović B.
Hadžović E.
Kostić S.
Bosnalijek d.d. Jukićeva 53, Sarajevo, Bosnia and Herzegovina
E-mail: berina.p@bosnalijek.ba (B. Pilipović)
Electronic publication date: 2010 Sep 30
Print publication date: 2010 Jul-Sep
Volume: 78
Issue: 3
First page: 662
Last page: 662
Copyright: © author(s); licensee Österreichische Apotheker-Verlagsgesellschaft m. b. H., Vienna, Austria.
Copyright year: 2010
License: This is an Open Access article distributed under the terms of the Creative Commons Attribution License (http://creativecommons.org/licenses/by/3.0/), which permits unrestricted use, distribution, and reproduction in any medium, provided the original work is properly cited.

Tab. 1. Min	5	10	15	20	25	30	f2	
Sample 1	
	
% dissolution	24,29	47,88	68,32	86,01	98,99	100,35		
SD	1,29	3,31	3,99	4,44	3,54	1,39		
RSD	5,33	6,91	5,84	5,17	3,58	1,39	75.21	
	
Sample 2	
	
% dissolution	21,42	43,00	62,35	78,86	91,72	97,85		
SD	2,57	4,07	5,07	5,69	4,95	1,99		
RSD	11,98	9,46	8,13	7,22	5,40	2,04	78,22	
	
Sample3	
	
% dissolution	22,18	44,78	65,45	82,85	95,47	98,47		
SD	3,60	5,78	6,65	6,38	4,20	1,02		
RSD	16,21	12,90	10,15	7,70	4,40	1,04	99.18	
	
Reference drug	
	
% dissolution	22,28	44,96	65,41	82,66	94,84	98,52		
SD	4,00	8,20	9,90	12,10	11,10	6,41		
RSD	17,95	18,25	15,13	14,64	11,70	6,51		

JOURNAL INFORMATION
==============================
NLM Title Abbreviation: Sci Pharm
Journal ID: Sci Pharm
NLM Journal ID: 0026251
Journal ID: Scientia Pharmaceutica

Scientia Pharmaceutica

ISSN: 0036-8709
EISSN: 2218-0532
Publisher: Österreichische Apotheker-Verlagsgesellschaft, m. b. H.

ARTICLE INFORMATION
==============================
DOI: 10.3797/scipharm.cespt.8.PMS36
Article ID: scipharm.2010.78.663
Subjects: Conference abstract PMS36

Numerical Simulation of Film Formation in Tablet Coating

Toschkoff G. 1
Suzzi D. 1
Reiter F. 3
Tritthart W. 3
Schlingmann M. 3
Khinast J. G. 1 2
1 Research Center Pharmaceutical Engineering, Graz, Austria
2 Institute for Process Engineering, Graz University of Technology, Graz, Austria
3 G.L. Pharma GmbH, Lannach, Austria
E-mail: gregor.toschkoff@rcpe.at (G. Toschkoff), khinast@tugraz.at (J. G. Khinast)
Electronic publication date: 2010 Sep 30
Print publication date: 2010 Jul-Sep
Volume: 78
Issue: 3
First page: 663
Last page: 663
Copyright: © author(s); licensee Österreichische Apotheker-Verlagsgesellschaft m. b. H., Vienna, Austria.
Copyright year: 2010
License: This is an Open Access article distributed under the terms of the Creative Commons Attribution License (http://creativecommons.org/licenses/by/3.0/), which permits unrestricted use, distribution, and reproduction in any medium, provided the original work is properly cited.

Fig. 1 Film formation on a single vertical tablet after 0.3 s in the spray zone.

JOURNAL INFORMATION
==============================
NLM Title Abbreviation: Sci Pharm
Journal ID: Sci Pharm
NLM Journal ID: 0026251
Journal ID: Scientia Pharmaceutica

Scientia Pharmaceutica

ISSN: 0036-8709
EISSN: 2218-0532
Publisher: Österreichische Apotheker-Verlagsgesellschaft, m. b. H.

ARTICLE INFORMATION
==============================
DOI: 10.3797/scipharm.cespt.8.PMS37
Article ID: scipharm.2010.78.664
Subjects: Conference abstract PMS37

Crystallization of APIs in a Continuously Seeded Tubular Crystallizer

Eder R. J. P.
Schmitt E.
Grill J.
Maier M.
Innerhofer S.
Radl S.
Gruber-Wölfler H.
Khinast J. G.
Institute for Process and Particle Engineering, Graz University of Technology, Graz, Austria
E-mail: khinast@tugraz.at (J. G. Khinast)
Electronic publication date: 2010 Sep 30
Print publication date: 2010 Jul-Sep
Volume: 78
Issue: 3
First page: 664
Last page: 664
Copyright: © author(s); licensee Österreichische Apotheker-Verlagsgesellschaft m. b. H., Vienna, Austria.
Copyright year: 2010
License: This is an Open Access article distributed under the terms of the Creative Commons Attribution License (http://creativecommons.org/licenses/by/3.0/), which permits unrestricted use, distribution, and reproduction in any medium, provided the original work is properly cited.

JOURNAL INFORMATION
==============================
NLM Title Abbreviation: Sci Pharm
Journal ID: Sci Pharm
NLM Journal ID: 0026251
Journal ID: Scientia Pharmaceutica

Scientia Pharmaceutica

ISSN: 0036-8709
EISSN: 2218-0532
Publisher: Österreichische Apotheker-Verlagsgesellschaft, m. b. H.

ARTICLE INFORMATION
==============================
DOI: 10.3797/scipharm.cespt.8.PMS38
Article ID: scipharm.2010.78.665
Subjects: Conference abstract PMS38

Multivariate Image Analysis, Histogram Matching and Statistical Process Control Chart Assisted Nucleation Detection of Pharmaceutical Crystallization processes

Simon L. L. 1
Abbou oucherif K. 2
Nagy Z. K. 3
Hungerbuhler K. 1
1 ETH Zurich, Institute of Chemical and Bioengineering, Zurich, Switzerland
2 New-Mexico Institute of Technology, Department of Chemical Engineering, Socorro, USA
3 Loughborough University, Chemical Engineering Department, Loughborough, United Kingdom
E-mail: Levente.simon@chem.ethz.ch (L. L. Simon)
Electronic publication date: 2010 Sep 30
Print publication date: 2010 Jul-Sep
Volume: 78
Issue: 3
First page: 665
Last page: 665
Copyright: © author(s); licensee Österreichische Apotheker-Verlagsgesellschaft m. b. H., Vienna, Austria.
Copyright year: 2010
License: This is an Open Access article distributed under the terms of the Creative Commons Attribution License (http://creativecommons.org/licenses/by/3.0/), which permits unrestricted use, distribution, and reproduction in any medium, provided the original work is properly cited.

JOURNAL INFORMATION
==============================
NLM Title Abbreviation: Sci Pharm
Journal ID: Sci Pharm
NLM Journal ID: 0026251
Journal ID: Scientia Pharmaceutica

Scientia Pharmaceutica

ISSN: 0036-8709
EISSN: 2218-0532
Publisher: Österreichische Apotheker-Verlagsgesellschaft, m. b. H.

ARTICLE INFORMATION
==============================
DOI: 10.3797/scipharm.cespt.8.PMS39
Article ID: scipharm.2010.78.666
Subjects: Conference abstract PMS39

Evaluation of Binding Agent’s Effect on the Compressibility of Poorly Compactable Drugs

Sáska Z. 1
Dredán J. 1
Balogh E. 1
Luhn O. 2
Antal I. 1
1 Semmelweis University, Department of Pharmaceutics, Budapest, Hungary
2 Beneo-Palatinit GmbH, Mannheim, Germany
E-mail: saska.zsofia@gmail.com (Z. Sáska); antist@gyok.sote.hu (I. Antal)
Electronic publication date: 2010 Sep 30
Print publication date: 2010 Jul-Sep
Volume: 78
Issue: 3
First page: 666
Last page: 666
Copyright: © author(s); licensee Österreichische Apotheker-Verlagsgesellschaft m. b. H., Vienna, Austria.
Copyright year: 2010
License: This is an Open Access article distributed under the terms of the Creative Commons Attribution License (http://creativecommons.org/licenses/by/3.0/), which permits unrestricted use, distribution, and reproduction in any medium, provided the original work is properly cited.

JOURNAL INFORMATION
==============================
NLM Title Abbreviation: Sci Pharm
Journal ID: Sci Pharm
NLM Journal ID: 0026251
Journal ID: Scientia Pharmaceutica

Scientia Pharmaceutica

ISSN: 0036-8709
EISSN: 2218-0532
Publisher: Österreichische Apotheker-Verlagsgesellschaft, m. b. H.

ARTICLE INFORMATION
==============================
DOI: 10.3797/scipharm.cespt.8.PMS40
Article ID: scipharm.2010.78.667
Subjects: Conference abstract PMS40

Simulation of Mixing Processes by Means of Mesh-free Particle Approach

Kussmann C.
Drage P.
qpunkt GmbH, Graz, Austria
E-mail: christian.kussmann@qpunkt.at (C. Kussmann)
Electronic publication date: 2010 Sep 30
Print publication date: 2010 Jul-Sep
Volume: 78
Issue: 3
First page: 667
Last page: 667
Copyright: © author(s); licensee Österreichische Apotheker-Verlagsgesellschaft m. b. H., Vienna, Austria.
Copyright year: 2010
License: This is an Open Access article distributed under the terms of the Creative Commons Attribution License (http://creativecommons.org/licenses/by/3.0/), which permits unrestricted use, distribution, and reproduction in any medium, provided the original work is properly cited.

JOURNAL INFORMATION
==============================
NLM Title Abbreviation: Sci Pharm
Journal ID: Sci Pharm
NLM Journal ID: 0026251
Journal ID: Scientia Pharmaceutica

Scientia Pharmaceutica

ISSN: 0036-8709
EISSN: 2218-0532
Publisher: Österreichische Apotheker-Verlagsgesellschaft, m. b. H.

ARTICLE INFORMATION
==============================
DOI: 10.3797/scipharm.cespt.8.PMS41
Article ID: scipharm.2010.78.668
Subjects: Conference abstract PMS41

Continuous Flow Manufacturing for API: From Potential Evaluation to Production Scale Realization

Kirschneck D.
Microinnova Engineering GmbH, Reininghausstrasse 13a, 8020 Graz, Austria
E-mail: Dirk.Kirschneck@microinnova.com
Electronic publication date: 2010 Sep 30
Print publication date: 2010 Jul-Sep
Volume: 78
Issue: 3
First page: 668
Last page: 668
Copyright: © author(s); licensee Österreichische Apotheker-Verlagsgesellschaft m. b. H., Vienna, Austria.
Copyright year: 2010
License: This is an Open Access article distributed under the terms of the Creative Commons Attribution License (http://creativecommons.org/licenses/by/3.0/), which permits unrestricted use, distribution, and reproduction in any medium, provided the original work is properly cited.

JOURNAL INFORMATION
==============================
NLM Title Abbreviation: Sci Pharm
Journal ID: Sci Pharm
NLM Journal ID: 0026251
Journal ID: Scientia Pharmaceutica

Scientia Pharmaceutica

ISSN: 0036-8709
EISSN: 2218-0532
Publisher: Österreichische Apotheker-Verlagsgesellschaft, m. b. H.

ARTICLE INFORMATION
==============================
DOI: 10.3797/scipharm.cespt.8.PMS42
Article ID: scipharm.2010.78.669
Subjects: Conference abstract PMS42

Optimization of Freeze Drying Process Using Isomalt as Bulking Agent

Kovács K. 1
Kállai N. 1
Stampf G. 1
Klebovich I. 1
Ludányi K. 1
Luhn O. 2
Antal I. 1
1 Department of Pharmaceutics, Semmelweis University, Budapest, Hungary
2 BENEO-Palatinit GmbH, Mannheim, Germany
E-mail: kovkrist@gyok.sote.hu (K. Kovács)
Electronic publication date: 2010 Sep 30
Print publication date: 2010 Jul-Sep
Volume: 78
Issue: 3
First page: 669
Last page: 669
Copyright: © author(s); licensee Österreichische Apotheker-Verlagsgesellschaft m. b. H., Vienna, Austria.
Copyright year: 2010
License: This is an Open Access article distributed under the terms of the Creative Commons Attribution License (http://creativecommons.org/licenses/by/3.0/), which permits unrestricted use, distribution, and reproduction in any medium, provided the original work is properly cited.

JOURNAL INFORMATION
==============================
NLM Title Abbreviation: Sci Pharm
Journal ID: Sci Pharm
NLM Journal ID: 0026251
Journal ID: Scientia Pharmaceutica

Scientia Pharmaceutica

ISSN: 0036-8709
EISSN: 2218-0532
Publisher: Österreichische Apotheker-Verlagsgesellschaft, m. b. H.

ARTICLE INFORMATION
==============================
DOI: 10.3797/scipharm.cespt.8.PMS43
Article ID: scipharm.2010.78.670
Subjects: Conference abstract PMS43

Micro-Systems Production: A Promising New Technique with Low Energy Consumption

Dalmoro A. 1
Barba A. A. 2
d'Amore M. 2
Lamberti G. 1
1 Dipartimento di Ingegneria Chimica e Alimentare, Università di Salerno, Fisciano, Italia
2 Dipartimento di Scienze Farmaceutiche, Università di Salerno, Fisciano, Italia
E-mails: adalmoro@unisa.it (A. Dalmoro), aabarba@unisa.it (A.A. Barba), mdamore@unisa.it (M. d’Amore), glamberti@unisa.it (G. Lamberti)
Electronic publication date: 2010 Sep 30
Print publication date: 2010 Jul-Sep
Volume: 78
Issue: 3
First page: 670
Last page: 670
Copyright: © author(s); licensee Österreichische Apotheker-Verlagsgesellschaft m. b. H., Vienna, Austria.
Copyright year: 2010
License: This is an Open Access article distributed under the terms of the Creative Commons Attribution License (http://creativecommons.org/licenses/by/3.0/), which permits unrestricted use, distribution, and reproduction in any medium, provided the original work is properly cited.

JOURNAL INFORMATION
==============================
NLM Title Abbreviation: Sci Pharm
Journal ID: Sci Pharm
NLM Journal ID: 0026251
Journal ID: Scientia Pharmaceutica

Scientia Pharmaceutica

ISSN: 0036-8709
EISSN: 2218-0532
Publisher: Österreichische Apotheker-Verlagsgesellschaft, m. b. H.

ARTICLE INFORMATION
==============================
DOI: 10.3797/scipharm.cespt.8.PNM01
Article ID: scipharm.2010.78.671
Subjects: Conference abstract PNM01

Liposomal PEG-Free Creams with Herbal Extracts: Stability Study

Arsić I. 1
Tadić V. 1
Runjaić-Antić D. 1
Đorđević S. 1
Homšek I. 2
1 Institute for Medicinal Plants Research “Dr Josif Pančić”, Tadeuša Košćuška 1, Belgrade, Serbia
2 Galenika Institute, A.D., Batajnički drum B.B., 11000 Belgrade, Serbia
E-mail: iarsic@mocbilja.rs (I. Arsic)
Electronic publication date: 2010 Sep 30
Print publication date: 2010 Jul-Sep
Volume: 78
Issue: 3
First page: 671
Last page: 671
Copyright: © author(s); licensee Österreichische Apotheker-Verlagsgesellschaft m. b. H., Vienna, Austria.
Copyright year: 2010
License: This is an Open Access article distributed under the terms of the Creative Commons Attribution License (http://creativecommons.org/licenses/by/3.0/), which permits unrestricted use, distribution, and reproduction in any medium, provided the original work is properly cited.

JOURNAL INFORMATION
==============================
NLM Title Abbreviation: Sci Pharm
Journal ID: Sci Pharm
NLM Journal ID: 0026251
Journal ID: Scientia Pharmaceutica

Scientia Pharmaceutica

ISSN: 0036-8709
EISSN: 2218-0532
Publisher: Österreichische Apotheker-Verlagsgesellschaft, m. b. H.

ARTICLE INFORMATION
==============================
DOI: 10.3797/scipharm.cespt.8.pnm02
Article ID: scipharm.2010.78.672
Subjects: Conference abstract PNM02

Influence of Carrier Particle Morphology on the Performance of Dry Powder inhalers

Littringer E. M. 1
Mescher A. 2
Schröttner H. 3
Walzel P. 2
Urbanetz N. A. 1
1 Research Center Pharmaceutical Engineering GmbH Graz, Austria
2 Department of Biochemical and Chemical Engineeering, TU Dortmund, Düsseldorf, Germany
3 Austrian Centre for Electron Microscopy and Nanoanalysis, TU Graz, Austria
E-mails: eva.littringer@tugraz.at (E. M. Littringer), axel.mescher@bci.tu-dortmund.de (A. Mescher), hartmuth.schroettner@felmi-zfe.at (H. Schröttner), p.walzel@bci.uni-dortmund.de (P. Walzel), nora.urbanetz@tugraz.at (N. A. Urbanetz)
Electronic publication date: 2010 Sep 30
Print publication date: 2010 Jul-Sep
Volume: 78
Issue: 3
First page: 672
Last page: 672
Copyright: © author(s); licensee Österreichische Apotheker-Verlagsgesellschaft m. b. H., Vienna, Austria.
Copyright year: 2010
License: This is an Open Access article distributed under the terms of the Creative Commons Attribution License (http://creativecommons.org/licenses/by/3.0/), which permits unrestricted use, distribution, and reproduction in any medium, provided the original work is properly cited.

JOURNAL INFORMATION
==============================
NLM Title Abbreviation: Sci Pharm
Journal ID: Sci Pharm
NLM Journal ID: 0026251
Journal ID: Scientia Pharmaceutica

Scientia Pharmaceutica

ISSN: 0036-8709
EISSN: 2218-0532
Publisher: Österreichische Apotheker-Verlagsgesellschaft, m. b. H.

ARTICLE INFORMATION
==============================
DOI: 10.3797/scipharm.cespt.8.PNM03
Article ID: scipharm.2010.78.673
Subjects: Conference abstract PNM03

Determination of the Membrane-Buffer Partition Coefficient with Laurdan Labeled Liposomes

Först G.
Schubert R.
Department of Pharmaceutical Technology and Biopharmacy, University of Freiburg, Freiburg, Germany
E-mail: gesche.foerst@pharmazie.uni-freiburg.de (G. Först)
Electronic publication date: 2010 Sep 30
Print publication date: 2010 Jul-Sep
Volume: 78
Issue: 3
First page: 673
Last page: 673
Copyright: © author(s); licensee Österreichische Apotheker-Verlagsgesellschaft m. b. H., Vienna, Austria.
Copyright year: 2010
License: This is an Open Access article distributed under the terms of the Creative Commons Attribution License (http://creativecommons.org/licenses/by/3.0/), which permits unrestricted use, distribution, and reproduction in any medium, provided the original work is properly cited.

JOURNAL INFORMATION
==============================
NLM Title Abbreviation: Sci Pharm
Journal ID: Sci Pharm
NLM Journal ID: 0026251
Journal ID: Scientia Pharmaceutica

Scientia Pharmaceutica

ISSN: 0036-8709
EISSN: 2218-0532
Publisher: Österreichische Apotheker-Verlagsgesellschaft, m. b. H.

ARTICLE INFORMATION
==============================
DOI: 10.3797/scipharm.cespt.8.PNM04
Article ID: scipharm.2010.78.674
Subjects: Conference abstract PNM04

Integrin- and IGF1-Receptor-Mediated Liposomal siRNA Delivery to Alveolar Rhabdomyosarcoma Cells

Striepe S. 1
Rössler J. 2
Süss R. 1
1 Department of Pharmaceutical Technology and Biopharmacy, University of Freiburg, Freiburg, Germany
2 Department of Pediatrics and Adolescent Medicine, Division of Pediatric Hematology and Oncology, University of Freiburg, Freiburg, Germany
E-mail: stefanie.striepe@pharmazie.uni-freiburg.de (S. Striepe)
Electronic publication date: 2010 Sep 30
Print publication date: 2010 Jul-Sep
Volume: 78
Issue: 3
First page: 674
Last page: 674
Copyright: © author(s); licensee Österreichische Apotheker-Verlagsgesellschaft m. b. H., Vienna, Austria.
Copyright year: 2010
License: This is an Open Access article distributed under the terms of the Creative Commons Attribution License (http://creativecommons.org/licenses/by/3.0/), which permits unrestricted use, distribution, and reproduction in any medium, provided the original work is properly cited.

JOURNAL INFORMATION
==============================
NLM Title Abbreviation: Sci Pharm
Journal ID: Sci Pharm
NLM Journal ID: 0026251
Journal ID: Scientia Pharmaceutica

Scientia Pharmaceutica

ISSN: 0036-8709
EISSN: 2218-0532
Publisher: Österreichische Apotheker-Verlagsgesellschaft, m. b. H.

ARTICLE INFORMATION
==============================
DOI: 10.3797/scipharm.cespt.8.PNM05
Article ID: scipharm.2010.78.675
Subjects: Conference abstract PNM05

Fibrin Targeting with Ultrasound Active Antibody-Conjugated Liposomes

Marxer E. E. J. 1
Brüßler J. 1
Becker A. 2
Nimsky C. 2
Bakowsky U. 1
1 Department of Pharmaceutical Technology and Biopharmaceutics, Philipps-Universität, Marburg, Germany
2 Department of neuro surgery, Universitätsklinikum Gießen und Marburg GmbH, Philipps-Universität, Marburg, Germany
E-mails: elena.marxer@staff.uni-marburg.de (E. E. J. Marxer), ubakowsky@aol.com (U. Bakowsky)
Electronic publication date: 2010 Sep 30
Print publication date: 2010 Jul-Sep
Volume: 78
Issue: 3
First page: 675
Last page: 675
Copyright: © author(s); licensee Österreichische Apotheker-Verlagsgesellschaft m. b. H., Vienna, Austria.
Copyright year: 2010
License: This is an Open Access article distributed under the terms of the Creative Commons Attribution License (http://creativecommons.org/licenses/by/3.0/), which permits unrestricted use, distribution, and reproduction in any medium, provided the original work is properly cited.

The authors would like to thank the DFG “Forschergruppe Nanohale 695” for support.

JOURNAL INFORMATION
==============================
NLM Title Abbreviation: Sci Pharm
Journal ID: Sci Pharm
NLM Journal ID: 0026251
Journal ID: Scientia Pharmaceutica

Scientia Pharmaceutica

ISSN: 0036-8709
EISSN: 2218-0532
Publisher: Österreichische Apotheker-Verlagsgesellschaft, m. b. H.

ARTICLE INFORMATION
==============================
DOI: 10.3797/scipharm.cespt.8.PNM06
Article ID: scipharm.2010.78.676
Subjects: Conference abstract PNM06

Freeze-Drying of Melatonin-Loaded Lecithin/Chitosan Nanoparticles

Hafner A. 1
Dürrigl M. 2
Filipović-Grčić J. 1
1 Dept. of Pharmaceutics, Faculty of Pharmacy and Biochemistry, University of Zagreb, Zagreb, Croatia
2 PLIVA Croatia Ltd., Research and Development, Zagreb, Croatia
E-mails: ahafner@pharma.hr (A. Hafner), marjana.durrigl@pliva.com (M. Dürrigl), jfilipov@pharma.hr (J. Filipović-Grčić)
Electronic publication date: 2010 Sep 30
Print publication date: 2010 Jul-Sep
Volume: 78
Issue: 3
First page: 676
Last page: 676
Copyright: © author(s); licensee Österreichische Apotheker-Verlagsgesellschaft m. b. H., Vienna, Austria.
Copyright year: 2010
License: This is an Open Access article distributed under the terms of the Creative Commons Attribution License (http://creativecommons.org/licenses/by/3.0/), which permits unrestricted use, distribution, and reproduction in any medium, provided the original work is properly cited.

JOURNAL INFORMATION
==============================
NLM Title Abbreviation: Sci Pharm
Journal ID: Sci Pharm
NLM Journal ID: 0026251
Journal ID: Scientia Pharmaceutica

Scientia Pharmaceutica

ISSN: 0036-8709
EISSN: 2218-0532
Publisher: Österreichische Apotheker-Verlagsgesellschaft, m. b. H.

ARTICLE INFORMATION
==============================
DOI: 10.3797/scipharm.cespt.8.PNM07
Article ID: scipharm.2010.78.677
Subjects: Conference abstract PNM07

Enhanced Imaging of Atherosclerotic Lesions in ApoE-deficient Mice using Nanotechnology

Almer G. 1
Wernig K. 2
Saba-Lepek M. 3
Haj-Yahya S. 1
Kellner J. 4
Rattenberger J. 5
Gries A. 4
Prassl R. 3
Zimmer A. 2
Mangge H. 1
1 Clinical Institute for Medical and Chemical Diagnosis, Medical University, Graz, Austria
2 Institute of Pharmaceutical Science, Department of Pharmaceutical Technology, University of Graz, Austria
3 Institute of Biophysics and Nanosystems Research, Austrian Academy of Sciences, Graz, Austria
4 Institute of Physiology, Medical University, Graz, Austria
5 Institute for Electron Microscopy and Fine Structure Research, Graz University of Technology, Austria
E-mails: gunter.almer@edu.uni-graz.at (G. Almer), harald.mangge@meduni-graz.at (H. Mangge)
Electronic publication date: 2010 Sep 30
Print publication date: 2010 Jul-Sep
Volume: 78
Issue: 3
First page: 677
Last page: 677
Copyright: © author(s); licensee Österreichische Apotheker-Verlagsgesellschaft m. b. H., Vienna, Austria.
Copyright year: 2010
License: This is an Open Access article distributed under the terms of the Creative Commons Attribution License (http://creativecommons.org/licenses/by/3.0/), which permits unrestricted use, distribution, and reproduction in any medium, provided the original work is properly cited.

JOURNAL INFORMATION
==============================
NLM Title Abbreviation: Sci Pharm
Journal ID: Sci Pharm
NLM Journal ID: 0026251
Journal ID: Scientia Pharmaceutica

Scientia Pharmaceutica

ISSN: 0036-8709
EISSN: 2218-0532
Publisher: Österreichische Apotheker-Verlagsgesellschaft, m. b. H.

ARTICLE INFORMATION
==============================
DOI: 10.3797/scipharm.cespt.8.PNM08
Article ID: scipharm.2010.78.678
Subjects: Conference abstract PNM08

Permeability Studies on the Ocular Absorbance of Nanostructured Materials Across the Cornea

Blass S. 1
Teubl B. 1
Fröhlich E. 2
Meindl C. 2
Rabensteiner D. F. 3
Trummer G. 3
Schmut O. 3
Zimmer A. 1
Roblegg E. 1
1 Institute of Pharmaceutical Sciences/ Pharmaceutical Technology, Karl-Franzens University Graz, Austria
2 Center of Medical Research, Medical University Graz, Austria
3 Department of Ophthalmology, Medical University Graz, Austria
E-mail: sandra.blass@uni-graz.at (S. Blass)
Electronic publication date: 2010 Sep 30
Print publication date: 2010 Jul-Sep
Volume: 78
Issue: 3
First page: 678
Last page: 678
Copyright: © author(s); licensee Österreichische Apotheker-Verlagsgesellschaft m. b. H., Vienna, Austria.
Copyright year: 2010
License: This is an Open Access article distributed under the terms of the Creative Commons Attribution License (http://creativecommons.org/licenses/by/3.0/), which permits unrestricted use, distribution, and reproduction in any medium, provided the original work is properly cited.

JOURNAL INFORMATION
==============================
NLM Title Abbreviation: Sci Pharm
Journal ID: Sci Pharm
NLM Journal ID: 0026251
Journal ID: Scientia Pharmaceutica

Scientia Pharmaceutica

ISSN: 0036-8709
EISSN: 2218-0532
Publisher: Österreichische Apotheker-Verlagsgesellschaft, m. b. H.

ARTICLE INFORMATION
==============================
DOI: 10.3797/scipharm.cespt.8.PNM09
Article ID: scipharm.2010.78.679
Subjects: Conference abstract PNM09

The Comparison of Methods to Assess In Vitro Drug Release from Nanoparticles

Grabnar P. A. 1
Kerč J. 2
Homar M. 2
Kristl J. 1
1 University of Ljubljana, Faculty of Pharmacy, 1000 Ljubljana, Slovenia
2 Lek Pharmaceuticals d. d., Research and Development, 1000 Ljubljana, Slovenia
E-mail: ahlinp@ffa.uni-lj.si (P. A. Grabnar)
Electronic publication date: 2010 Sep 30
Print publication date: 2010 Jul-Sep
Volume: 78
Issue: 3
First page: 679
Last page: 679
Copyright: © author(s); licensee Österreichische Apotheker-Verlagsgesellschaft m. b. H., Vienna, Austria.
Copyright year: 2010
License: This is an Open Access article distributed under the terms of the Creative Commons Attribution License (http://creativecommons.org/licenses/by/3.0/), which permits unrestricted use, distribution, and reproduction in any medium, provided the original work is properly cited.

JOURNAL INFORMATION
==============================
NLM Title Abbreviation: Sci Pharm
Journal ID: Sci Pharm
NLM Journal ID: 0026251
Journal ID: Scientia Pharmaceutica

Scientia Pharmaceutica

ISSN: 0036-8709
EISSN: 2218-0532
Publisher: Österreichische Apotheker-Verlagsgesellschaft, m. b. H.

ARTICLE INFORMATION
==============================
DOI: 10.3797/scipharm.cespt.8.PNM10
Article ID: scipharm.2010.78.680
Subjects: Conference abstract PNM10

Ketoprofen Loaded Poly(D,L-lactic acid) Nanospheres for Potential Transdermal/Dermal Application: Preparation and Physical Characterization

Vučen S. T. 1
Vuleta G. 2
Ignjatović N. 3
Uskoković D. 3
1 Faculty of Medicine, Department of Pharmacy, University of Banja Luka, Bosnia and Herzegovina
2 Department of the pharmaceutical technology and cosmetology, Faculty of Pharmacy, University of Belgrade, Serbia
3 Institute of technical sciences of the Serbian Academy of Sciences and Arts, Belgrade, Serbia
E-mail: sonjatorbica@gmail.com (S. T. Vučen)
Electronic publication date: 2010 Sep 30
Print publication date: 2010 Jul-Sep
Volume: 78
Issue: 3
First page: 680
Last page: 680
Copyright: © author(s); licensee Österreichische Apotheker-Verlagsgesellschaft m. b. H., Vienna, Austria.
Copyright year: 2010
License: This is an Open Access article distributed under the terms of the Creative Commons Attribution License (http://creativecommons.org/licenses/by/3.0/), which permits unrestricted use, distribution, and reproduction in any medium, provided the original work is properly cited.

JOURNAL INFORMATION
==============================
NLM Title Abbreviation: Sci Pharm
Journal ID: Sci Pharm
NLM Journal ID: 0026251
Journal ID: Scientia Pharmaceutica

Scientia Pharmaceutica

ISSN: 0036-8709
EISSN: 2218-0532
Publisher: Österreichische Apotheker-Verlagsgesellschaft, m. b. H.

ARTICLE INFORMATION
==============================
DOI: 10.3797/scipharm.cespt.8.PNM11
Article ID: scipharm.2010.78.681
Subjects: Conference abstract PNM11

Polyglycerol Fatty Acid Ester Based Lipid Nanoparticles: Physical Stability and Crystalline Status

Kovacevic A. 1
Savic S. 1
Milic J. 1
Müller R. H. 2
Keck C. M. 2 3
1 Department of Pharmaceutical Technology and Cosmetology, Faculty of Pharmacy, University of Belgrade, Serbia
2 Department of Pharmaceutics, Biopharmaceutics & NutriCosmetics, Free University of Berlin, Germany
3 Fachhochschule Kaiserslautern, University of Applied Sciences Kaiserslautern, Angewandte Logistik- und Polymerwissenschaften Pirmasens, Carl-Schurz-Straße 10-16, 66953 Pirmasens, Germany
E-mails: andjelkakovacevic@yahoo.com (A. Kovacevic), nanoteam@gmx.com (R. H. Müller),
Electronic publication date: 2010 Sep 30
Print publication date: 2010 Jul-Sep
Volume: 78
Issue: 3
First page: 681
Last page: 681
Copyright: © author(s); licensee Österreichische Apotheker-Verlagsgesellschaft m. b. H., Vienna, Austria.
Copyright year: 2010
License: This is an Open Access article distributed under the terms of the Creative Commons Attribution License (http://creativecommons.org/licenses/by/3.0/), which permits unrestricted use, distribution, and reproduction in any medium, provided the original work is properly cited.

The authors would like to acknowledge the financial support obtained from DAAD (Deutscher Akademischer Austauschdienst) for the present research.

JOURNAL INFORMATION
==============================
NLM Title Abbreviation: Sci Pharm
Journal ID: Sci Pharm
NLM Journal ID: 0026251
Journal ID: Scientia Pharmaceutica

Scientia Pharmaceutica

ISSN: 0036-8709
EISSN: 2218-0532
Publisher: Österreichische Apotheker-Verlagsgesellschaft, m. b. H.

ARTICLE INFORMATION
==============================
DOI: 10.3797/scipharm.cespt.8.PNM12
Article ID: scipharm.2010.78.682
Subjects: Conference abstract PNM12

Distinct Trafficking of Solid Lipid Nanoparticles within Cells

Kristl J.
Teskač K.
Pajk S.
Pečar S.
Faculty of Pharmacy, University of Ljubljana, Ljubljana, Slovenia
E-mail: julijana.kristl@ffa.uni-lj.si (J. Kristl)
Electronic publication date: 2010 Sep 30
Print publication date: 2010 Jul-Sep
Volume: 78
Issue: 3
First page: 682
Last page: 682
Copyright: © author(s); licensee Österreichische Apotheker-Verlagsgesellschaft m. b. H., Vienna, Austria.
Copyright year: 2010
License: This is an Open Access article distributed under the terms of the Creative Commons Attribution License (http://creativecommons.org/licenses/by/3.0/), which permits unrestricted use, distribution, and reproduction in any medium, provided the original work is properly cited.

Fig. 1. Imaging of internalized SLN (marked with arrows) located next to the nuclei (rounded structure). A - SLN-SPP are seen distinctly as (blue) dots, while B - SLN-C are hardly recognized due to the broadly distributed fluorescence from 6-coumarin (green). Bar is 10 μm.

JOURNAL INFORMATION
==============================
NLM Title Abbreviation: Sci Pharm
Journal ID: Sci Pharm
NLM Journal ID: 0026251
Journal ID: Scientia Pharmaceutica

Scientia Pharmaceutica

ISSN: 0036-8709
EISSN: 2218-0532
Publisher: Österreichische Apotheker-Verlagsgesellschaft, m. b. H.

ARTICLE INFORMATION
==============================
DOI: 10.3797/scipharm.cespt.8.PNM13
Article ID: scipharm.2010.78.683
Subjects: Conference abstract PNM13

Effects of Mixing Mode on the Formation of Silica Nanoparticles Synthesized via Aqueous Sol-Gel Process

Andreani T. 1
Souza A. L. R. 1 2
Silva A. M. 1 3
Martins C. L. 4
Souto E. B. 4
1 Department of Biology and Environment, University of Trás-os-Montes and Alto Douro, Vila Real, Portugal
2 Faculty of Pharmaceutical Sciences, UNESP, Araraquara, Brazil
3 Centre for Research and Technology of Agro-Environmental and Biological Sciences, Vila Real, Portugal
4 Department of Pharmaceutical Technology, Faculty of Health Sciences, UFP, Porto, Portugal
E-mail: ttandreani@hotmail.com (T. Andreani)
Electronic publication date: 2010 Sep 30
Print publication date: 2010 Jul-Sep
Volume: 78
Issue: 3
First page: 683
Last page: 683
Copyright: © author(s); licensee Österreichische Apotheker-Verlagsgesellschaft m. b. H., Vienna, Austria.
Copyright year: 2010
License: This is an Open Access article distributed under the terms of the Creative Commons Attribution License (http://creativecommons.org/licenses/by/3.0/), which permits unrestricted use, distribution, and reproduction in any medium, provided the original work is properly cited.

This work was supported by FCT, SFRH/BD/60640/2009 to T. Andreani and by Capes to A.L.R. Souza. Whe are thankyfull to Dr. L. Fernandes for TEM assistance.

JOURNAL INFORMATION
==============================
NLM Title Abbreviation: Sci Pharm
Journal ID: Sci Pharm
NLM Journal ID: 0026251
Journal ID: Scientia Pharmaceutica

Scientia Pharmaceutica

ISSN: 0036-8709
EISSN: 2218-0532
Publisher: Österreichische Apotheker-Verlagsgesellschaft, m. b. H.

ARTICLE INFORMATION
==============================
DOI: 10.3797/scipharm.cespt.8.PNM14
Article ID: scipharm.2010.78.684
Subjects: Conference abstract PNM14

Itraconazol-Loaded Nanostructured Lipid Carriers (NLC) for Pulmonary Application

Pardeike J. 1
Weber S. 1
Zarfl H. P. 2
Zimmer A. 1
1 Institute of Pharmaceutical Sciences, Department of Pharmaceutical Technology, Karl-Franzens Universität Graz, Universitätsplatz 1, 8010 Graz, Austria
2 Private Veterinary Sugery, Auen 23, 9400 Wolfsberg, Austria
E-mails: jana.pardeike@uni-graz.at (J. Pardeike), h.p.zarfl@gmail.com (H.P. Zarfl)
Electronic publication date: 2010 Sep 30
Print publication date: 2010 Jul-Sep
Volume: 78
Issue: 3
First page: 684
Last page: 684
Copyright: © author(s); licensee Österreichische Apotheker-Verlagsgesellschaft m. b. H., Vienna, Austria.
Copyright year: 2010
License: This is an Open Access article distributed under the terms of the Creative Commons Attribution License (http://creativecommons.org/licenses/by/3.0/), which permits unrestricted use, distribution, and reproduction in any medium, provided the original work is properly cited.

JOURNAL INFORMATION
==============================
NLM Title Abbreviation: Sci Pharm
Journal ID: Sci Pharm
NLM Journal ID: 0026251
Journal ID: Scientia Pharmaceutica

Scientia Pharmaceutica

ISSN: 0036-8709
EISSN: 2218-0532
Publisher: Österreichische Apotheker-Verlagsgesellschaft, m. b. H.

ARTICLE INFORMATION
==============================
DOI: 10.3797/scipharm.cespt.8.PNM15
Article ID: scipharm.2010.78.685
Subjects: Conference abstract PNM15

Spray Drying as a Novel Method for Producing Nanoparticles Intended for the Application in DPI’s

Schnepfleitner S.
Urbanetz N. A.
Institute for Process and Particle Engineering, University of Technology, Graz, Austria
E-mail: sabrina.schnepfleitner@tugraz.at (S. Schnepfleitner), nora.urbanetz@tugraz.at (N. A. Urbanetz)
Electronic publication date: 2010 Sep 30
Print publication date: 2010 Jul-Sep
Volume: 78
Issue: 3
First page: 685
Last page: 685
Copyright: © author(s); licensee Österreichische Apotheker-Verlagsgesellschaft m. b. H., Vienna, Austria.
Copyright year: 2010
License: This is an Open Access article distributed under the terms of the Creative Commons Attribution License (http://creativecommons.org/licenses/by/3.0/), which permits unrestricted use, distribution, and reproduction in any medium, provided the original work is properly cited.

JOURNAL INFORMATION
==============================
NLM Title Abbreviation: Sci Pharm
Journal ID: Sci Pharm
NLM Journal ID: 0026251
Journal ID: Scientia Pharmaceutica

Scientia Pharmaceutica

ISSN: 0036-8709
EISSN: 2218-0532
Publisher: Österreichische Apotheker-Verlagsgesellschaft, m. b. H.

ARTICLE INFORMATION
==============================
DOI: 10.3797/scipharm.cespt.8.PNM16
Article ID: scipharm.2010.78.686
Subjects: Conference abstract PNM16

Protamine Based siRNA-Nanoparticles: Particle Composition Dependant Influences on RNA Interference Efficiency

Reischl D. 1
Jantscher-Krenn E. 1
Bernhart E. 2
Sattler W. 2
Zimmer A. 1
1 Institute of Pharmaceutical Sciences, Department of Pharmaceutical Technology, Karl-Franzens-University Graz, Austria
2 Institute of Molecular Biology and Biochemistry, Medical University Graz, Austria
E-mail: daniela.reischl@uni-graz.at (D. Reischl)
Electronic publication date: 2010 Sep 30
Print publication date: 2010 Jul-Sep
Volume: 78
Issue: 3
First page: 686
Last page: 686
Copyright: © author(s); licensee Österreichische Apotheker-Verlagsgesellschaft m. b. H., Vienna, Austria.
Copyright year: 2010
License: This is an Open Access article distributed under the terms of the Creative Commons Attribution License (http://creativecommons.org/licenses/by/3.0/), which permits unrestricted use, distribution, and reproduction in any medium, provided the original work is properly cited.

The authors would like to acknowledge the financial support of the study by FFG, Bridge-program: P810994.

JOURNAL INFORMATION
==============================
NLM Title Abbreviation: Sci Pharm
Journal ID: Sci Pharm
NLM Journal ID: 0026251
Journal ID: Scientia Pharmaceutica

Scientia Pharmaceutica

ISSN: 0036-8709
EISSN: 2218-0532
Publisher: Österreichische Apotheker-Verlagsgesellschaft, m. b. H.

ARTICLE INFORMATION
==============================
DOI: 10.3797/scipharm.cespt.8.PNM17
Article ID: scipharm.2010.78.687
Subjects: Conference abstract PNM17

Polyacrylic Acid-Protamine Nanoparticles for Scale-Up Experiments in a Microreactor

Petschacher C. 1
Eitzlmayr A. 2
Radl S. 2
Suzzi D. 2
Khinast J. G. 2
Zimmer A. 1
1 Institute of Pharmaceutical Sciences, Karl - Franzens - University, Graz, Austria
2 Research Center Pharmaceutical Engineering, Graz, Austria
E-Mail: christina.petschacher@uni-graz.at (C. Petschacher)
Electronic publication date: 2010 Sep 30
Print publication date: 2010 Jul-Sep
Volume: 78
Issue: 3
First page: 687
Last page: 687
Copyright: © author(s); licensee Österreichische Apotheker-Verlagsgesellschaft m. b. H., Vienna, Austria.
Copyright year: 2010
License: This is an Open Access article distributed under the terms of the Creative Commons Attribution License (http://creativecommons.org/licenses/by/3.0/), which permits unrestricted use, distribution, and reproduction in any medium, provided the original work is properly cited.

JOURNAL INFORMATION
==============================
NLM Title Abbreviation: Sci Pharm
Journal ID: Sci Pharm
NLM Journal ID: 0026251
Journal ID: Scientia Pharmaceutica

Scientia Pharmaceutica

ISSN: 0036-8709
EISSN: 2218-0532
Publisher: Österreichische Apotheker-Verlagsgesellschaft, m. b. H.

ARTICLE INFORMATION
==============================
DOI: 10.3797/scipharm.cespt.8.PNM18
Article ID: scipharm.2010.78.688
Subjects: Conference abstract PNM18

In Vitro Sonothrombolysis of Human Blood Clots: Preliminary Results

Petit B. 1 2
Gaud E. 2
Polliart F. 1 2
Colevret D. 2
Schneider M. 2
Guilbert-Brigger I. 2
Yan F. 2
Tranquart F. 2
Allémann E. 1
1 School of Pharmaceutical Sciences, University of Geneva, University of Lausanne, Geneva, Switzerland
2 Bracco Research S.A, Plan-les-Ouates, Geneva, Switzerland
E-mails: benedicte.petit@unige.ch (B. Petit), eric.allemann@unige.ch (E. Allémann)
Electronic publication date: 2010 Sep 30
Print publication date: 2010 Jul-Sep
Volume: 78
Issue: 3
First page: 688
Last page: 688
Copyright: © author(s); licensee Österreichische Apotheker-Verlagsgesellschaft m. b. H., Vienna, Austria.
Copyright year: 2010
License: This is an Open Access article distributed under the terms of the Creative Commons Attribution License (http://creativecommons.org/licenses/by/3.0/), which permits unrestricted use, distribution, and reproduction in any medium, provided the original work is properly cited.

JOURNAL INFORMATION
==============================
NLM Title Abbreviation: Sci Pharm
Journal ID: Sci Pharm
NLM Journal ID: 0026251
Journal ID: Scientia Pharmaceutica

Scientia Pharmaceutica

ISSN: 0036-8709
EISSN: 2218-0532
Publisher: Österreichische Apotheker-Verlagsgesellschaft, m. b. H.

ARTICLE INFORMATION
==============================
DOI: 10.3797/scipharm.cespt.8.PNM19
Article ID: scipharm.2010.78.689
Subjects: Conference abstract PNM19

Bioavailability of Dexamethasone from Nonionic Surfactant/Chitosan Micelle System

Pepić I.
Hafner A.
Lovrić J.
Filipović-Grčić J.
Dept. of Pharmaceutics, Faculty of Pharmacy and Biochemistry, University of Zagreb, Zagreb, Croatia
E-mails: ipepic@pharma.hr (I. Pepić), ahafner@pharma.hr (A. Hafner), jlovric@pharma.hr (J. Lovrić), jfilipov@pharma.hr (J. Filipović-Grčić)
Electronic publication date: 2010 Sep 30
Print publication date: 2010 Jul-Sep
Volume: 78
Issue: 3
First page: 689
Last page: 689
Copyright: © author(s); licensee Österreichische Apotheker-Verlagsgesellschaft m. b. H., Vienna, Austria.
Copyright year: 2010
License: This is an Open Access article distributed under the terms of the Creative Commons Attribution License (http://creativecommons.org/licenses/by/3.0/), which permits unrestricted use, distribution, and reproduction in any medium, provided the original work is properly cited.

JOURNAL INFORMATION
==============================
NLM Title Abbreviation: Sci Pharm
Journal ID: Sci Pharm
NLM Journal ID: 0026251
Journal ID: Scientia Pharmaceutica

Scientia Pharmaceutica

ISSN: 0036-8709
EISSN: 2218-0532
Publisher: Österreichische Apotheker-Verlagsgesellschaft, m. b. H.

ARTICLE INFORMATION
==============================
DOI: 10.3797/scipharm.cespt.8.PPAT01
Article ID: scipharm.2010.78.690
Subjects: Conference abstract PPAT01

Lecithin Based Pseudoternary Phase Diagrams

Gosenca M.
Gašperlin M.
Faculty of pharmacy, University of Ljubljana, Ljubljana, Slovenia
E-mail: mirjam.gosenca@ffa.uni-lj.si (M. Gosenca)
Electronic publication date: 2010 Sep 30
Print publication date: 2010 Jul-Sep
Volume: 78
Issue: 3
First page: 690
Last page: 690
Copyright: © author(s); licensee Österreichische Apotheker-Verlagsgesellschaft m. b. H., Vienna, Austria.
Copyright year: 2010
License: This is an Open Access article distributed under the terms of the Creative Commons Attribution License (http://creativecommons.org/licenses/by/3.0/), which permits unrestricted use, distribution, and reproduction in any medium, provided the original work is properly cited.

JOURNAL INFORMATION
==============================
NLM Title Abbreviation: Sci Pharm
Journal ID: Sci Pharm
NLM Journal ID: 0026251
Journal ID: Scientia Pharmaceutica

Scientia Pharmaceutica

ISSN: 0036-8709
EISSN: 2218-0532
Publisher: Österreichische Apotheker-Verlagsgesellschaft, m. b. H.

ARTICLE INFORMATION
==============================
DOI: 10.3797/scipharm.cespt.8.PPAT02
Article ID: scipharm.2010.78.691
Subjects: Conference abstract PPAT02

The Use of Small and Wide Angle X-Ray Scattering (SWAXS) for the Physico-Chemical Characterization of Solid Dispersions

Metzler M. 1
Hodzic A. 1
Laggner P. 2 3
Urbanetz N. A. 1 4
1 Research Center Pharmaceutical Engineering GmbH, Graz, Austria
2 Institut of Biophysics and X-Ray Structure Research, Austrian Academy of Sciences, Graz, Austria
3 HECUS X-Ray Systems, Graz, Austria
4 Institute for Process and Particle Engineering, Graz University of technology, Graz, Austria
E-mail: aden.hodzic@tugraz.at (A. Hodzic)
Electronic publication date: 2010 Sep 30
Print publication date: 2010 Jul-Sep
Volume: 78
Issue: 3
First page: 691
Last page: 691
Copyright: © author(s); licensee Österreichische Apotheker-Verlagsgesellschaft m. b. H., Vienna, Austria.
Copyright year: 2010
License: This is an Open Access article distributed under the terms of the Creative Commons Attribution License (http://creativecommons.org/licenses/by/3.0/), which permits unrestricted use, distribution, and reproduction in any medium, provided the original work is properly cited.

JOURNAL INFORMATION
==============================
NLM Title Abbreviation: Sci Pharm
Journal ID: Sci Pharm
NLM Journal ID: 0026251
Journal ID: Scientia Pharmaceutica

Scientia Pharmaceutica

ISSN: 0036-8709
EISSN: 2218-0532
Publisher: Österreichische Apotheker-Verlagsgesellschaft, m. b. H.

ARTICLE INFORMATION
==============================
DOI: 10.3797/scipharm.cespt.8.PPAT03
Article ID: scipharm.2010.78.692
Subjects: Conference abstract PPAT03

Qualification of NIR Spectral Imaging for the Pharmaceutical Industry

Moor J. 1
Koller D. M. 1
Heigl N. 1
Balak N. 1
Burgstaller M. 2
Kerschhaggl P. 2
Khinast J. G. 1 3
1 Research Center Pharmaceutical Engineering GmbH, Graz, Austria
2 EVK DI KERSCHHAGGL GmbH, Raaba, Austria
3 Institute for Process and Particle Engineering, Graz University of Technology, Graz, Austria
E-mails: daniel.koller@rcpe.at (D. M. Koller), khinast@tugraz.at (J. G. Khinast)
Electronic publication date: 2010 Sep 30
Print publication date: 2010 Jul-Sep
Volume: 78
Issue: 3
First page: 692
Last page: 692
Copyright: © author(s); licensee Österreichische Apotheker-Verlagsgesellschaft m. b. H., Vienna, Austria.
Copyright year: 2010
License: This is an Open Access article distributed under the terms of the Creative Commons Attribution License (http://creativecommons.org/licenses/by/3.0/), which permits unrestricted use, distribution, and reproduction in any medium, provided the original work is properly cited.

JOURNAL INFORMATION
==============================
NLM Title Abbreviation: Sci Pharm
Journal ID: Sci Pharm
NLM Journal ID: 0026251
Journal ID: Scientia Pharmaceutica

Scientia Pharmaceutica

ISSN: 0036-8709
EISSN: 2218-0532
Publisher: Österreichische Apotheker-Verlagsgesellschaft, m. b. H.

ARTICLE INFORMATION
==============================
DOI: 10.3797/scipharm.cespt.8.PPAT04
Article ID: scipharm.2010.78.693
Subjects: Conference abstract PPAT04

Evaluation of API-Distribution and Coating Thickness by NIR Spectroscopy and Raman Chemical Mapping

Hörl G. 1
Heigl N. 1
Koller D. M. 1
Tritthart W. 3
Reiter F. 3
Schlingmann M. 3
Khinast J. G. 1 2
1 Research Center Pharmaceutical Engineering GmbH, Graz, Austria
2 Institute for Process and Particle Engineering, Graz University of Technology, Graz, Austria
3 G.L.Pharma GmbH, Lannach, Austria
E-mails: gudrun.hoerl@rcpe.at (G. Hörl), khinast@tugraz.at (J. G. Khinast)
Electronic publication date: 2010 Sep 30
Print publication date: 2010 Jul-Sep
Volume: 78
Issue: 3
First page: 693
Last page: 693
Copyright: © author(s); licensee Österreichische Apotheker-Verlagsgesellschaft m. b. H., Vienna, Austria.
Copyright year: 2010
License: This is an Open Access article distributed under the terms of the Creative Commons Attribution License (http://creativecommons.org/licenses/by/3.0/), which permits unrestricted use, distribution, and reproduction in any medium, provided the original work is properly cited.

JOURNAL INFORMATION
==============================
NLM Title Abbreviation: Sci Pharm
Journal ID: Sci Pharm
NLM Journal ID: 0026251
Journal ID: Scientia Pharmaceutica

Scientia Pharmaceutica

ISSN: 0036-8709
EISSN: 2218-0532
Publisher: Österreichische Apotheker-Verlagsgesellschaft, m. b. H.

ARTICLE INFORMATION
==============================
DOI: 10.3797/scipharm.cespt.8.PPAT05
Article ID: scipharm.2010.78.694
Subjects: Conference abstract PPAT05

Pharmaceutical Powder Compaction and Dissolution Analytics by Laboratory Small and Wide Angle X-Ray Scattering (SWAXS)

Hodzic A. 1
Kriechbaum M. 2 3
Tritthart W. 4
Reiter F. 4
Fraser S. 1
Khinast J. G. 1 5
Laggner P. 2 3
1 Research Center Pharmaceutical Engineering GmbH, Graz, Austria
2 Hecus X-Ray Systems GmbH, Graz, Austria
3 Institute of Biophysics and Nanosystems Research, Austrian Academy of Sciences, Graz, Austria
4 G.L. Pharma GmbH, Lannach, Austria
5 Institute for Process and Particle Engineering (IPPE), Graz University of Technology, Graz, Austria
E-mail: aden.hodzic@tugraz.at (A. Hodzic)
Electronic publication date: 2010 Sep 30
Print publication date: 2010 Jul-Sep
Volume: 78
Issue: 3
First page: 694
Last page: 694
Copyright: © author(s); licensee Österreichische Apotheker-Verlagsgesellschaft m. b. H., Vienna, Austria.
Copyright year: 2010
License: This is an Open Access article distributed under the terms of the Creative Commons Attribution License (http://creativecommons.org/licenses/by/3.0/), which permits unrestricted use, distribution, and reproduction in any medium, provided the original work is properly cited.

JOURNAL INFORMATION
==============================
NLM Title Abbreviation: Sci Pharm
Journal ID: Sci Pharm
NLM Journal ID: 0026251
Journal ID: Scientia Pharmaceutica

Scientia Pharmaceutica

ISSN: 0036-8709
EISSN: 2218-0532
Publisher: Österreichische Apotheker-Verlagsgesellschaft, m. b. H.

ARTICLE INFORMATION
==============================
DOI: 10.3797/scipharm.cespt.8.PPAT06
Article ID: scipharm.2010.78.695
Subjects: Conference abstract PPAT06

Kinetic Pathway Analysis of Aggregation of Therapeutic Proteins

Roessl U. 1 2
Wiesbauer J. 1 2
Leitgeb S. 1 2
Nidetzky B. 2
1 Research Center Pharmaceutical Engineering GmbH, Graz, Austria
2 Institute of Biotechnology and Biochemical Engineering, University of Technology Graz, Graz, Austria
E-mail: Ulrich.roessl@rcpe.at (U. Roessl)
Electronic publication date: 2010 Sep 30
Print publication date: 2010 Jul-Sep
Volume: 78
Issue: 3
First page: 695
Last page: 695
Copyright: © author(s); licensee Österreichische Apotheker-Verlagsgesellschaft m. b. H., Vienna, Austria.
Copyright year: 2010
License: This is an Open Access article distributed under the terms of the Creative Commons Attribution License (http://creativecommons.org/licenses/by/3.0/), which permits unrestricted use, distribution, and reproduction in any medium, provided the original work is properly cited.

JOURNAL INFORMATION
==============================
NLM Title Abbreviation: Sci Pharm
Journal ID: Sci Pharm
NLM Journal ID: 0026251
Journal ID: Scientia Pharmaceutica

Scientia Pharmaceutica

ISSN: 0036-8709
EISSN: 2218-0532
Publisher: Österreichische Apotheker-Verlagsgesellschaft, m. b. H.

ARTICLE INFORMATION
==============================
DOI: 10.3797/scipharm.cespt.8.PPAT07
Article ID: scipharm.2010.78.696
Subjects: Conference abstract PPAT07

Experimental and Mathematical Analysis of Hepatic Uptake

Grandjean T. 1
Lench A. 2
Yates J. W. T. 3
Chappell M.J. 1
O‘Donnell C. J. 3
1 School of Engineering, University of Warwick, Coventry, CV4 7AL, United Kingdom
2 Departments of Pharmacy and Pharmacology, University of Bath, Bath, United Kingdom
3 DMPK Department, Astrazeneca, Alderley Park, Macclesfield, United Kingdom
E-mail: james.yates@astrazeneca.com (J. Yates)
Electronic publication date: 2010 Sep 30
Print publication date: 2010 Jul-Sep
Volume: 78
Issue: 3
First page: 696
Last page: 696
Copyright: © author(s); licensee Österreichische Apotheker-Verlagsgesellschaft m. b. H., Vienna, Austria.
Copyright year: 2010
License: This is an Open Access article distributed under the terms of the Creative Commons Attribution License (http://creativecommons.org/licenses/by/3.0/), which permits unrestricted use, distribution, and reproduction in any medium, provided the original work is properly cited.

JOURNAL INFORMATION
==============================
NLM Title Abbreviation: Sci Pharm
Journal ID: Sci Pharm
NLM Journal ID: 0026251
Journal ID: Scientia Pharmaceutica

Scientia Pharmaceutica

ISSN: 0036-8709
EISSN: 2218-0532
Publisher: Österreichische Apotheker-Verlagsgesellschaft, m. b. H.

ARTICLE INFORMATION
==============================
DOI: 10.3797/scipharm.cespt.8.PPAT08
Article ID: scipharm.2010.78.697
Subjects: Conference abstract PPAT08

Chemical Structure Role of Cell-Membrane Hydrocarbon Chain in Interactions with Antihypertensive Drugs (SARTANs)

Hodzic A. 1
Mavromoustakos T. 2
Zoumpoulakis P. 2
Rappolt M. 3
Laggner P. 3
Pabst G. 3
1 Research Center Pharmaceutical Engineering GmbH, Graz, Austria
2 National Hellenic Research Foundation, Institute of Organic and Pharmaceutical Chemistry, Athens, Greece
3 Austrian Academy of Sciences, Institute of Biophysics and Nanosystems Research, Graz, Austria
E-mail: aden.hodzic@tugraz.at (A. Hodzic)
Electronic publication date: 2010 Sep 30
Print publication date: 2010 Jul-Sep
Volume: 78
Issue: 3
First page: 697
Last page: 697
Copyright: © author(s); licensee Österreichische Apotheker-Verlagsgesellschaft m. b. H., Vienna, Austria.
Copyright year: 2010
License: This is an Open Access article distributed under the terms of the Creative Commons Attribution License (http://creativecommons.org/licenses/by/3.0/), which permits unrestricted use, distribution, and reproduction in any medium, provided the original work is properly cited.

JOURNAL INFORMATION
==============================
NLM Title Abbreviation: Sci Pharm
Journal ID: Sci Pharm
NLM Journal ID: 0026251
Journal ID: Scientia Pharmaceutica

Scientia Pharmaceutica

ISSN: 0036-8709
EISSN: 2218-0532
Publisher: Österreichische Apotheker-Verlagsgesellschaft, m. b. H.

ARTICLE INFORMATION
==============================
DOI: 10.3797/scipharm.cespt.8.PPAT09
Article ID: scipharm.2010.78.698
Subjects: Conference abstract PPAT09

HS-SPME-GCMS Method Development for Sensitive Diisopropylphenol Analysis Suited for Adsorption/Absorption Studies and Cleaning Validations

Pickl K. E. 1
Adamek V. 2
Gorges R. 2
Sinner F. M. 1
1 Institute of Medical Technologies and Healthmanagement, JOANNEUM RESEARCH, Graz, Austria
2 Research Center for Pharmaceutical Engineering, Graz, Austria
E-mail: karin.pickl@joanneum.at (K. E. Pickl)
Electronic publication date: 2010 Sep 30
Print publication date: 2010 Jul-Sep
Volume: 78
Issue: 3
First page: 698
Last page: 698
Copyright: © author(s); licensee Österreichische Apotheker-Verlagsgesellschaft m. b. H., Vienna, Austria.
Copyright year: 2010
License: This is an Open Access article distributed under the terms of the Creative Commons Attribution License (http://creativecommons.org/licenses/by/3.0/), which permits unrestricted use, distribution, and reproduction in any medium, provided the original work is properly cited.

JOURNAL INFORMATION
==============================
NLM Title Abbreviation: Sci Pharm
Journal ID: Sci Pharm
NLM Journal ID: 0026251
Journal ID: Scientia Pharmaceutica

Scientia Pharmaceutica

ISSN: 0036-8709
EISSN: 2218-0532
Publisher: Österreichische Apotheker-Verlagsgesellschaft, m. b. H.

ARTICLE INFORMATION
==============================
DOI: 10.3797/scipharm.cespt.8.PPAT10
Article ID: scipharm.2010.78.699
Subjects: Conference abstract PPAT10

Use of Forced Degradation Studies on S-(−)-Amlodipine Besylate to Generate Information on the Degradation Products

Hadžidedić Š. 1
Uzunović A. 1
Pilipović S. 1
El-Arino S. Kocova 2
1 Agency for Medicinal Products and Medical Devices of B&H, 71000 Sarajevo, Bosnia and Herzegovina
2 National Research Centre, Tahrir Street, Cairo, Egypt
E-mail: s.hadzidedic@alims.gov.ba (S. Hadžidedić)
Electronic publication date: 2010 Sep 30
Print publication date: 2010 Jul-Sep
Volume: 78
Issue: 3
First page: 699
Last page: 699
Copyright: © author(s); licensee Österreichische Apotheker-Verlagsgesellschaft m. b. H., Vienna, Austria.
Copyright year: 2010
License: This is an Open Access article distributed under the terms of the Creative Commons Attribution License (http://creativecommons.org/licenses/by/3.0/), which permits unrestricted use, distribution, and reproduction in any medium, provided the original work is properly cited.

This work was supported by ALIMS B&H.

JOURNAL INFORMATION
==============================
NLM Title Abbreviation: Sci Pharm
Journal ID: Sci Pharm
NLM Journal ID: 0026251
Journal ID: Scientia Pharmaceutica

Scientia Pharmaceutica

ISSN: 0036-8709
EISSN: 2218-0532
Publisher: Österreichische Apotheker-Verlagsgesellschaft, m. b. H.

ARTICLE INFORMATION
==============================
DOI: 10.3797/scipharm.cespt.8.PPAT11
Article ID: scipharm.2010.78.700
Subjects: Conference abstract PPAT11

Quality-by-Design Based Characterization of Pharmaceutical Processes by Means of Numerical Simulations

Adam S. 1
Suzzi D. 1
Radl S. 1 2
Toschkoff G. 1
Radeke C. 1
Hörmann T. 1
Iannuccelli M. 1
Khinast J. G. 1 2
1 Research Center Pharmaceutical Engineering GmbH, Graz, Austria
2 Graz University of Technology, Institute for Process and Particle Engineering, Graz, Austria
E-mails: siegfried.adam@rcpe.at (S. Adam), khinast@tugraz.at (J. G. Khinast),
Electronic publication date: 2010 Sep 30
Print publication date: 2010 Jul-Sep
Volume: 78
Issue: 3
First page: 700
Last page: 700
Copyright: © author(s); licensee Österreichische Apotheker-Verlagsgesellschaft m. b. H., Vienna, Austria.
Copyright year: 2010
License: This is an Open Access article distributed under the terms of the Creative Commons Attribution License (http://creativecommons.org/licenses/by/3.0/), which permits unrestricted use, distribution, and reproduction in any medium, provided the original work is properly cited.

JOURNAL INFORMATION
==============================
NLM Title Abbreviation: Sci Pharm
Journal ID: Sci Pharm
NLM Journal ID: 0026251
Journal ID: Scientia Pharmaceutica

Scientia Pharmaceutica

ISSN: 0036-8709
EISSN: 2218-0532
Publisher: Österreichische Apotheker-Verlagsgesellschaft, m. b. H.

ARTICLE INFORMATION
==============================
DOI: 10.3797/scipharm.cespt.8.PPAT12
Article ID: scipharm.2010.78.701
Subjects: Conference abstract PPAT12

Application of Experimental Design for Screening Study of Dissolution Test Conditions: Levothyroxine Sodium Immediate-Release Tablets

Kocic I.
Homsek I.
Dacevic M.
R&D Institute, Galenika ad, Belgrade, Serbia
E-mail: ivana_kocic@yahoo.com (I. Kocic)
Electronic publication date: 2010 Sep 30
Print publication date: 2010 Jul-Sep
Volume: 78
Issue: 3
First page: 701
Last page: 701
Copyright: © author(s); licensee Österreichische Apotheker-Verlagsgesellschaft m. b. H., Vienna, Austria.
Copyright year: 2010
License: This is an Open Access article distributed under the terms of the Creative Commons Attribution License (http://creativecommons.org/licenses/by/3.0/), which permits unrestricted use, distribution, and reproduction in any medium, provided the original work is properly cited.

JOURNAL INFORMATION
==============================
NLM Title Abbreviation: Sci Pharm
Journal ID: Sci Pharm
NLM Journal ID: 0026251
Journal ID: Scientia Pharmaceutica

Scientia Pharmaceutica

ISSN: 0036-8709
EISSN: 2218-0532
Publisher: Österreichische Apotheker-Verlagsgesellschaft, m. b. H.

ARTICLE INFORMATION
==============================
DOI: 10.3797/scipharm.cespt.8.PPAT13
Article ID: scipharm.2010.78.702
Subjects: Conference abstract PPAT13

In Vitro-In Silico Tools to Identify Biorelevant Dissolution Specifications for the Selected Poorly-Soluble Model Drugs

Grbic S.
Parojcic J.
Djuric Z.
Department of Pharmaceutical Technology and Cosmetology, Faculty of Pharmacy, University of Belgrade, Belgrade, Serbia
E-mail: gsandra@pharmacy.bg.ac.rs (S. Grbic)
Electronic publication date: 2010 Sep 30
Print publication date: 2010 Jul-Sep
Volume: 78
Issue: 3
First page: 702
Last page: 702
Copyright: © author(s); licensee Österreichische Apotheker-Verlagsgesellschaft m. b. H., Vienna, Austria.
Copyright year: 2010
License: This is an Open Access article distributed under the terms of the Creative Commons Attribution License (http://creativecommons.org/licenses/by/3.0/), which permits unrestricted use, distribution, and reproduction in any medium, provided the original work is properly cited.

JOURNAL INFORMATION
==============================
NLM Title Abbreviation: Sci Pharm
Journal ID: Sci Pharm
NLM Journal ID: 0026251
Journal ID: Scientia Pharmaceutica

Scientia Pharmaceutica

ISSN: 0036-8709
EISSN: 2218-0532
Publisher: Österreichische Apotheker-Verlagsgesellschaft, m. b. H.

ARTICLE INFORMATION
==============================
DOI: 10.3797/scipharm.cespt.8.PPAT14
Article ID: scipharm.2010.78.703
Subjects: Conference abstract PPAT14

Tailored Stationary Phases for Continuous Electro-Chromatographic Separation of APIs

Gruber-Woelfler H. 1
Feenstra P. W. 1
Braunbruck M.-G. 1
Laskowski R. 2
Bart H. J. 2
Khinast J. G. 1
1 Institute for Process and Particle Engineering, Graz University of Technology, Graz, Austria
2 Lehrstuhl für Thermische Verfahrenstechnik, Techn. Universität Kaiserslautern, Kaiserslautern, Germany
E-mail: woelfler@tugraz.at (H. Gruber-Woelfler)
Electronic publication date: 2010 Sep 30
Print publication date: 2010 Jul-Sep
Volume: 78
Issue: 3
First page: 703
Last page: 703
Copyright: © author(s); licensee Österreichische Apotheker-Verlagsgesellschaft m. b. H., Vienna, Austria.
Copyright year: 2010
License: This is an Open Access article distributed under the terms of the Creative Commons Attribution License (http://creativecommons.org/licenses/by/3.0/), which permits unrestricted use, distribution, and reproduction in any medium, provided the original work is properly cited.

JOURNAL INFORMATION
==============================
NLM Title Abbreviation: Sci Pharm
Journal ID: Sci Pharm
NLM Journal ID: 0026251
Journal ID: Scientia Pharmaceutica

Scientia Pharmaceutica

ISSN: 0036-8709
EISSN: 2218-0532
Publisher: Österreichische Apotheker-Verlagsgesellschaft, m. b. H.

ARTICLE INFORMATION
==============================
DOI: 10.3797/scipharm.cespt.8.PPAT15
Article ID: scipharm.2010.78.704
Subjects: Conference abstract PPAT15

A Simple High-Throughput Method for Determination of Pregabalin in Pharmaceutical Dosage Forms using a Microplate Fluorescence Reader

Vovk T.
Martinc B.
Grabnar I.
Faculty of Pharmacy, University of Ljubljana, Ljubljana, Slovenia
E-mail: tomaz.vovk@ffa.uni-lj.si (T. Vovk)
Electronic publication date: 2010 Sep 30
Print publication date: 2010 Jul-Sep
Volume: 78
Issue: 3
First page: 704
Last page: 704
Copyright: © author(s); licensee Österreichische Apotheker-Verlagsgesellschaft m. b. H., Vienna, Austria.
Copyright year: 2010
License: This is an Open Access article distributed under the terms of the Creative Commons Attribution License (http://creativecommons.org/licenses/by/3.0/), which permits unrestricted use, distribution, and reproduction in any medium, provided the original work is properly cited.

JOURNAL INFORMATION
==============================
NLM Title Abbreviation: Sci Pharm
Journal ID: Sci Pharm
NLM Journal ID: 0026251
Journal ID: Scientia Pharmaceutica

Scientia Pharmaceutica

ISSN: 0036-8709
EISSN: 2218-0532
Publisher: Österreichische Apotheker-Verlagsgesellschaft, m. b. H.

ARTICLE INFORMATION
==============================
DOI: 10.3797/scipharm.cespt.8.PPAT16
Article ID: scipharm.2010.78.705
Subjects: Conference abstract PPAT16

Evaluation of Sodium Naproxen Hydrated Forms by Isoconversional Analysis

Malaj L. 1
Martena V. 2
Corridoni A. 2
Bacchiocchi C. 2
Di Martino P. 2
1 University of Tirana, Tirana, Albania
2 University of Camerino, Camerino, Italy
E-mail: piera.dimartino@unicam.it (P. Di Martino).
Electronic publication date: 2010 Sep 30
Print publication date: 2010 Jul-Sep
Volume: 78
Issue: 3
First page: 705
Last page: 705
Copyright: © author(s); licensee Österreichische Apotheker-Verlagsgesellschaft m. b. H., Vienna, Austria.
Copyright year: 2010
License: This is an Open Access article distributed under the terms of the Creative Commons Attribution License (http://creativecommons.org/licenses/by/3.0/), which permits unrestricted use, distribution, and reproduction in any medium, provided the original work is properly cited.

JOURNAL INFORMATION
==============================
NLM Title Abbreviation: Sci Pharm
Journal ID: Sci Pharm
NLM Journal ID: 0026251
Journal ID: Scientia Pharmaceutica

Scientia Pharmaceutica

ISSN: 0036-8709
EISSN: 2218-0532
Publisher: Österreichische Apotheker-Verlagsgesellschaft, m. b. H.

ARTICLE INFORMATION
==============================
DOI: 10.3797/scipharm.cespt.8.PPAT17
Article ID: scipharm.2010.78.706
Subjects: Conference abstract PPAT17

Multidisciplinary Approach on Characterizing Composites of Vinpocetine and Crospovidone Obtained by Solid State Activation

Hasa D. 1
Voinovich D. 1
Perissutti B. 1
Bonifacio A. 2
Grassi M. 2
Franceschinis E. 3
Dall’Acqua S. 3
Speh M. 1–4
Plavec J. 4
1 Department of Pharmaceutical Sciences, University of Trieste, Trieste, Italy
2 Department of Chemical Engineering, University of Trieste, Trieste, Italy
3 Department of Pharmaceutical Sciences, University of Padua, Padua, Italy
4 Slovenian NMR Centre, National Institute of Chemistry, Ljubljana, Slovenia
E-mail: vojnovic@units.it (D. Voinovich)
Electronic publication date: 2010 Sep 30
Print publication date: 2010 Jul-Sep
Volume: 78
Issue: 3
First page: 706
Last page: 706
Copyright: © author(s); licensee Österreichische Apotheker-Verlagsgesellschaft m. b. H., Vienna, Austria.
Copyright year: 2010
License: This is an Open Access article distributed under the terms of the Creative Commons Attribution License (http://creativecommons.org/licenses/by/3.0/), which permits unrestricted use, distribution, and reproduction in any medium, provided the original work is properly cited.

The authors thank Linnea for the kind gift of Vinpocetine and Fondazione CRTrieste for funding.

JOURNAL INFORMATION
==============================
NLM Title Abbreviation: Sci Pharm
Journal ID: Sci Pharm
NLM Journal ID: 0026251
Journal ID: Scientia Pharmaceutica

Scientia Pharmaceutica

ISSN: 0036-8709
EISSN: 2218-0532
Publisher: Österreichische Apotheker-Verlagsgesellschaft, m. b. H.

ARTICLE INFORMATION
==============================
DOI: 10.3797/scipharm.cespt.8.PPAT18
Article ID: scipharm.2010.78.707
Subjects: Conference abstract PPAT18

Enzyme-Catalyzed Synthesis of 2-Azido-2-deoxy-D-glucose under Catalysis by Recombinant Cellobiose Phosphorylase (CeP)

Lubura B. 1
Gabra N. 1
Starkl P. 2
Viernstein H. 1
Unger F. M. 1
1 Department of Pharmaceutical Technology and Biopharmaceutics, University of Vienna, Althanstrasse 14, 1090 Vienna, Austria
2 Department of Pathophysiology, Medical University of Vienna, Waehringer Guertel 18–20, 1090 Vienna, Austria
E-mail: borjana.lubura@univie.ac.at (B. Lubura)
Electronic publication date: 2010 Sep 30
Print publication date: 2010 Jul-Sep
Volume: 78
Issue: 3
First page: 707
Last page: 707
Copyright: © author(s); licensee Österreichische Apotheker-Verlagsgesellschaft m. b. H., Vienna, Austria.
Copyright year: 2010
License: This is an Open Access article distributed under the terms of the Creative Commons Attribution License (http://creativecommons.org/licenses/by/3.0/), which permits unrestricted use, distribution, and reproduction in any medium, provided the original work is properly cited.

JOURNAL INFORMATION
==============================
NLM Title Abbreviation: Sci Pharm
Journal ID: Sci Pharm
NLM Journal ID: 0026251
Journal ID: Scientia Pharmaceutica

Scientia Pharmaceutica

ISSN: 0036-8709
EISSN: 2218-0532
Publisher: Österreichische Apotheker-Verlagsgesellschaft, m. b. H.

ARTICLE INFORMATION
==============================
DOI: 10.3797/scipharm.cespt.8.PPAT19
Article ID: scipharm.2010.78.708
Subjects: Conference abstract PPAT19

Characterization of Biomedical Surfaces by Streaming Potential Measurements

Luxbacher T.
Anton Paar GmbH, Graz, Austria
E-mail: thomas.luxbacher@anton-paar.com (Thomas Luxbacher)
Electronic publication date: 2010 Sep 30
Print publication date: 2010 Jul-Sep
Volume: 78
Issue: 3
First page: 708
Last page: 708
Copyright: © author(s); licensee Österreichische Apotheker-Verlagsgesellschaft m. b. H., Vienna, Austria.
Copyright year: 2010
License: This is an Open Access article distributed under the terms of the Creative Commons Attribution License (http://creativecommons.org/licenses/by/3.0/), which permits unrestricted use, distribution, and reproduction in any medium, provided the original work is properly cited.

JOURNAL INFORMATION
==============================
NLM Title Abbreviation: Sci Pharm
Journal ID: Sci Pharm
NLM Journal ID: 0026251
Journal ID: Scientia Pharmaceutica

Scientia Pharmaceutica

ISSN: 0036-8709
EISSN: 2218-0532
Publisher: Österreichische Apotheker-Verlagsgesellschaft, m. b. H.

ARTICLE INFORMATION
==============================
DOI: 10.3797/scipharm.cespt.8.PPAT20
Article ID: scipharm.2010.78.709
Subjects: Conference abstract PPAT20

Application of Dynamic Neural Networks and Fuzzy Algorithms in the Modeling of Drug Release

Petrović J. 1
Ibrić S. 1
Betz G. 2
Đurić Z. 1
1 Institute of Pharmaceutical Technology, Faculty of Pharmacy, University of Belgrade, Belgrade, Serbia
2 Institute of Pharmaceutical Technology, Pharmacenter, University of Basel, Basel, Switzerland
E-mail: jpetrovic@pharmacy.bg.ac.rs (J. Petrović)
Electronic publication date: 2010 Sep 30
Print publication date: 2010 Jul-Sep
Volume: 78
Issue: 3
First page: 709
Last page: 709
Copyright: © author(s); licensee Österreichische Apotheker-Verlagsgesellschaft m. b. H., Vienna, Austria.
Copyright year: 2010
License: This is an Open Access article distributed under the terms of the Creative Commons Attribution License (http://creativecommons.org/licenses/by/3.0/), which permits unrestricted use, distribution, and reproduction in any medium, provided the original work is properly cited.

JOURNAL INFORMATION
==============================
NLM Title Abbreviation: Sci Pharm
Journal ID: Sci Pharm
NLM Journal ID: 0026251
Journal ID: Scientia Pharmaceutica

Scientia Pharmaceutica

ISSN: 0036-8709
EISSN: 2218-0532
Publisher: Österreichische Apotheker-Verlagsgesellschaft, m. b. H.

ARTICLE INFORMATION
==============================
DOI: 10.3797/scipharm.cespt.8.PPAT21
Article ID: scipharm.2010.78.710
Subjects: Conference abstract PPAT21

Innovative Concepts for Personnel Locks in Clean Room Technology

Liebminger S. 1
Suzzi D. 1
Fraser S. 1
Schinagl G. 1
Radl S. 1 2
Oberauner L. 1 3
Aichner M. 1 3
Cardinale M. 3
Khinast J. 1 2
Berg G. 3
1 Research Center Pharmaceutical Engineering GmbH, Graz, Austria
2 Institute for Process- and Particle Engineering, University of Technology, Graz, Austria
3 Institute for Environmental Biotechnology, University of Technology, Graz, Austria
E-mail: stefan.liebminger@rcpe.at (S. Liebminger)
Electronic publication date: 2010 Sep 30
Print publication date: 2010 Jul-Sep
Volume: 78
Issue: 3
First page: 710
Last page: 710
Copyright: © author(s); licensee Österreichische Apotheker-Verlagsgesellschaft m. b. H., Vienna, Austria.
Copyright year: 2010
License: This is an Open Access article distributed under the terms of the Creative Commons Attribution License (http://creativecommons.org/licenses/by/3.0/), which permits unrestricted use, distribution, and reproduction in any medium, provided the original work is properly cited.

JOURNAL INFORMATION
==============================
NLM Title Abbreviation: Sci Pharm
Journal ID: Sci Pharm
NLM Journal ID: 0026251
Journal ID: Scientia Pharmaceutica

Scientia Pharmaceutica

ISSN: 0036-8709
EISSN: 2218-0532
Publisher: Österreichische Apotheker-Verlagsgesellschaft, m. b. H.

ARTICLE INFORMATION
==============================
DOI: 10.3797/scipharm.cespt.8.PPAT22
Article ID: scipharm.2010.78.711
Subjects: Conference abstract PPAT22

Mechanical Influences During Dissolution Testing in a Novel Peristaltic Movement Simulating Stirring Device

Bogataj M.
Glavač A.
Nagelj Kovačič N.
Mrhar A.
Faculty of Pharmacy, University of Ljubljana, Askerceva 7, SI-1000 Ljubljana, Slovenia
E-mail: marija.bogataj@ffa.uni-lj.si (M. Bogataj)
Electronic publication date: 2010 Sep 30
Print publication date: 2010 Jul-Sep
Volume: 78
Issue: 3
First page: 711
Last page: 711
Copyright: © author(s); licensee Österreichische Apotheker-Verlagsgesellschaft m. b. H., Vienna, Austria.
Copyright year: 2010
License: This is an Open Access article distributed under the terms of the Creative Commons Attribution License (http://creativecommons.org/licenses/by/3.0/), which permits unrestricted use, distribution, and reproduction in any medium, provided the original work is properly cited.

JOURNAL INFORMATION
==============================
NLM Title Abbreviation: Sci Pharm
Journal ID: Sci Pharm
NLM Journal ID: 0026251
Journal ID: Scientia Pharmaceutica

Scientia Pharmaceutica

ISSN: 0036-8709
EISSN: 2218-0532
Publisher: Österreichische Apotheker-Verlagsgesellschaft, m. b. H.

ARTICLE INFORMATION
==============================
DOI: 10.3797/scipharm.cespt.8.PPAT23
Article ID: scipharm.2010.78.712
Subjects: Conference abstract PPAT23

Caffeine Nucleation Detection Using a Matrix of External Turbidity Probes: Bulk Video Imaging (BVI)

Simon L. L. 1
Nagy Z. K. 2
Hungerbuhler K. 1
1 ETH Zurich, Institute of Chemical and Bioengineering, Zurich, Switzerland
2 Loughborough University, Chemical Engineering Department, Loughborough, United Kingdom
E-mail: Levente.simon@chem.ethz.ch (L. L. Simon)
Electronic publication date: 2010 Sep 30
Print publication date: 2010 Jul-Sep
Volume: 78
Issue: 3
First page: 712
Last page: 712
Copyright: © author(s); licensee Österreichische Apotheker-Verlagsgesellschaft m. b. H., Vienna, Austria.
Copyright year: 2010
License: This is an Open Access article distributed under the terms of the Creative Commons Attribution License (http://creativecommons.org/licenses/by/3.0/), which permits unrestricted use, distribution, and reproduction in any medium, provided the original work is properly cited.

JOURNAL INFORMATION
==============================
NLM Title Abbreviation: Sci Pharm
Journal ID: Sci Pharm
NLM Journal ID: 0026251
Journal ID: Scientia Pharmaceutica

Scientia Pharmaceutica

ISSN: 0036-8709
EISSN: 2218-0532
Publisher: Österreichische Apotheker-Verlagsgesellschaft, m. b. H.

ARTICLE INFORMATION
==============================
DOI: 10.3797/scipharm.cespt.8.PPAT24
Article ID: scipharm.2010.78.713
Subjects: Conference abstract PPAT24

Morphologically Directed Raman Spectroscopy as a Novel Tool to Monitor Changes in Particle Size and Shape in Solid Dosage Blends and Formulations

Levoguer C. L. 1
Kidder L. 2
Haber K. 2
1 Malvern Instruments Limited, Malvern, United Kingdom
2 Malvern Instruments Inc., Columbia MA, USA
E-mail: carl.levoguer@malvern.com (C. L. Levoguer)
Electronic publication date: 2010 Sep 30
Print publication date: 2010 Jul-Sep
Volume: 78
Issue: 3
First page: 713
Last page: 713
Copyright: © author(s); licensee Österreichische Apotheker-Verlagsgesellschaft m. b. H., Vienna, Austria.
Copyright year: 2010
License: This is an Open Access article distributed under the terms of the Creative Commons Attribution License (http://creativecommons.org/licenses/by/3.0/), which permits unrestricted use, distribution, and reproduction in any medium, provided the original work is properly cited.

JOURNAL INFORMATION
==============================
NLM Title Abbreviation: Sci Pharm
Journal ID: Sci Pharm
NLM Journal ID: 0026251
Journal ID: Scientia Pharmaceutica

Scientia Pharmaceutica

ISSN: 0036-8709
EISSN: 2218-0532
Publisher: Österreichische Apotheker-Verlagsgesellschaft, m. b. H.

ARTICLE INFORMATION
==============================
DOI: 10.3797/scipharm.cespt.8.PPAT25
Article ID: scipharm.2010.78.714
Subjects: Conference abstract PPAT25

Application of 14N NQR to the Study of Sulfanilamide, Piroxicam, and Nifedipine Polymorphism

Lavrič Z. 1
Pirnat J. 2
Lužnik J. 2
Seliger J. 3
Žagar V. 3
Trontelj Z. 2
Srčič S. 1
1 Faculty of Pharmacy, University of Ljubljana, Ljubljana, Slovenia
2 Institute of Mathematics, Physics and Mechanics, Ljubljana, Slovenia
3 Institute J. Stefan, Ljubljana, Slovenia
E-mail: zoran.lavric@ffa.uni-lj.si (Z. Lavrič)
Electronic publication date: 2010 Sep 30
Print publication date: 2010 Jul-Sep
Volume: 78
Issue: 3
First page: 714
Last page: 714
Copyright: © author(s); licensee Österreichische Apotheker-Verlagsgesellschaft m. b. H., Vienna, Austria.
Copyright year: 2010
License: This is an Open Access article distributed under the terms of the Creative Commons Attribution License (http://creativecommons.org/licenses/by/3.0/), which permits unrestricted use, distribution, and reproduction in any medium, provided the original work is properly cited.

JOURNAL INFORMATION
==============================
NLM Title Abbreviation: Sci Pharm
Journal ID: Sci Pharm
NLM Journal ID: 0026251
Journal ID: Scientia Pharmaceutica

Scientia Pharmaceutica

ISSN: 0036-8709
EISSN: 2218-0532
Publisher: Österreichische Apotheker-Verlagsgesellschaft, m. b. H.

ARTICLE INFORMATION
==============================
DOI: 10.3797/scipharm.cespt.8.PPAT26
Article ID: scipharm.2010.78.715
Subjects: Conference abstract PPAT26

Protein Characterization in Solution by Small-Angle X-Ray Scattering

Santner H.
Anton Paar GmbH, Graz, Austria
E-mail: heiner.Santner@anton-paar.com
Electronic publication date: 2010 Sep 30
Print publication date: 2010 Jul-Sep
Volume: 78
Issue: 3
First page: 715
Last page: 715
Copyright: © author(s); licensee Österreichische Apotheker-Verlagsgesellschaft m. b. H., Vienna, Austria.
Copyright year: 2010
License: This is an Open Access article distributed under the terms of the Creative Commons Attribution License (http://creativecommons.org/licenses/by/3.0/), which permits unrestricted use, distribution, and reproduction in any medium, provided the original work is properly cited.

JOURNAL INFORMATION
==============================
NLM Title Abbreviation: Sci Pharm
Journal ID: Sci Pharm
NLM Journal ID: 0026251
Journal ID: Scientia Pharmaceutica

Scientia Pharmaceutica

ISSN: 0036-8709
EISSN: 2218-0532
Publisher: Österreichische Apotheker-Verlagsgesellschaft, m. b. H.

ARTICLE INFORMATION
==============================
DOI: 10.3797/scipharm.cespt.8.POT01
Article ID: scipharm.2010.78.716
Subjects: Conference abstract POT01

The Potential of Sucrose Esters to be used as Oral Absorption Enhancers

Kis L. 1 2
Szűts A. 2
Otomo N. 3
Szabó-Révész P. 2
Deli M. A. 1
1 Laboratory of Molecular Neurobiology, Institute of Biophysics, Biological Research Center, Hungarian Academy of Sciences, Szeged, Hungary
2 Department of Pharmaceutical Technology, University of Szeged, Szeged, Hungary
3 Mitsubishi-Kagaku Foods Corporation, Tokyo, Japan
E-mail: kisslori@brc.hu (L. Kis)
Electronic publication date: 2010 Sep 30
Print publication date: 2010 Jul-Sep
Volume: 78
Issue: 3
First page: 716
Last page: 716
Copyright: © author(s); licensee Österreichische Apotheker-Verlagsgesellschaft m. b. H., Vienna, Austria.
Copyright year: 2010
License: This is an Open Access article distributed under the terms of the Creative Commons Attribution License (http://creativecommons.org/licenses/by/3.0/), which permits unrestricted use, distribution, and reproduction in any medium, provided the original work is properly cited.

JOURNAL INFORMATION
==============================
NLM Title Abbreviation: Sci Pharm
Journal ID: Sci Pharm
NLM Journal ID: 0026251
Journal ID: Scientia Pharmaceutica

Scientia Pharmaceutica

ISSN: 0036-8709
EISSN: 2218-0532
Publisher: Österreichische Apotheker-Verlagsgesellschaft, m. b. H.

ARTICLE INFORMATION
==============================
DOI: 10.3797/scipharm.cespt.8.POT02
Article ID: scipharm.2010.78.717
Subjects: Conference abstract POT02

Development of Intermediate Layer with Improved Diffusional Barrier

Jaklič M. T.
Jurečič R.
Lek Pharmaceuticals,d.d., Sandoz Development Center Slovenia, Verovškova 57, 1526 Ljubljana, Slovenia
E-mail: miha.jaklic@sandoz.com (M. T. Jaklič), rok.jurecic@sandoz.com (R. Jurečič)
Electronic publication date: 2010 Sep 30
Print publication date: 2010 Jul-Sep
Volume: 78
Issue: 3
First page: 717
Last page: 717
Copyright: © author(s); licensee Österreichische Apotheker-Verlagsgesellschaft m. b. H., Vienna, Austria.
Copyright year: 2010
License: This is an Open Access article distributed under the terms of the Creative Commons Attribution License (http://creativecommons.org/licenses/by/3.0/), which permits unrestricted use, distribution, and reproduction in any medium, provided the original work is properly cited.

JOURNAL INFORMATION
==============================
NLM Title Abbreviation: Sci Pharm
Journal ID: Sci Pharm
NLM Journal ID: 0026251
Journal ID: Scientia Pharmaceutica

Scientia Pharmaceutica

ISSN: 0036-8709
EISSN: 2218-0532
Publisher: Österreichische Apotheker-Verlagsgesellschaft, m. b. H.

ARTICLE INFORMATION
==============================
DOI: 10.3797/scipharm.cespt.8.POT03
Article ID: scipharm.2010.78.718
Subjects: Conference abstract POT03

A Coupled Kinetic-Dynamic Model Characterising Cell Cycle Response to an Anti-Cancer Agent

Atari M. I. 1
Chappell M. J. 1
Errington R. J. 2
Smith P. J. 2
Evans N. D. 1
1 School of Engineering, University of Warwick, Coventry, United Kingdom
2 University of Wales College of Medicine, Cardiff University, Cardiff, United Kingdom
E-mail: m.i.atari@warwick.ac.uk (M. I. Atari)
Electronic publication date: 2010 Sep 30
Print publication date: 2010 Jul-Sep
Volume: 78
Issue: 3
First page: 718
Last page: 718
Copyright: © author(s); licensee Österreichische Apotheker-Verlagsgesellschaft m. b. H., Vienna, Austria.
Copyright year: 2010
License: This is an Open Access article distributed under the terms of the Creative Commons Attribution License (http://creativecommons.org/licenses/by/3.0/), which permits unrestricted use, distribution, and reproduction in any medium, provided the original work is properly cited.

JOURNAL INFORMATION
==============================
NLM Title Abbreviation: Sci Pharm
Journal ID: Sci Pharm
NLM Journal ID: 0026251
Journal ID: Scientia Pharmaceutica

Scientia Pharmaceutica

ISSN: 0036-8709
EISSN: 2218-0532
Publisher: Österreichische Apotheker-Verlagsgesellschaft, m. b. H.

ARTICLE INFORMATION
==============================
DOI: 10.3797/scipharm.cespt.8.POT04
Article ID: scipharm.2010.78.719
Subjects: Conference abstract POT04

Natural Surfactant of Alkyl Polyglucoside Type: A Physicochemical Characterization of New Mixed Emulsifier

Lukic M. 1
Jaksic I. 1
Daniels R. 2
Vuleta G. 1
Savic S. 1
1 Department of Pharmaceutical Technology and Cosmetology, University of Belgrade, Belgrade, Serbia
2 Department of Pharmaceutical Technology, Eberhard-Karls Universität Tübingen, Tübingen, Germany
E-mail: mlukic@pharmacy.bg.rs (M. Lukic)
Electronic publication date: 2010 Sep 30
Print publication date: 2010 Jul-Sep
Volume: 78
Issue: 3
First page: 719
Last page: 719
Copyright: © author(s); licensee Österreichische Apotheker-Verlagsgesellschaft m. b. H., Vienna, Austria.
Copyright year: 2010
License: This is an Open Access article distributed under the terms of the Creative Commons Attribution License (http://creativecommons.org/licenses/by/3.0/), which permits unrestricted use, distribution, and reproduction in any medium, provided the original work is properly cited.

JOURNAL INFORMATION
==============================
NLM Title Abbreviation: Sci Pharm
Journal ID: Sci Pharm
NLM Journal ID: 0026251
Journal ID: Scientia Pharmaceutica

Scientia Pharmaceutica

ISSN: 0036-8709
EISSN: 2218-0532
Publisher: Österreichische Apotheker-Verlagsgesellschaft, m. b. H.

ARTICLE INFORMATION
==============================
DOI: 10.3797/scipharm.cespt.8.POT05
Article ID: scipharm.2010.78.720
Subjects: Conference abstract POT05

The Stability of Salmon Calcitonin Against Trypsin, Chymotrypsin and Neutrophil Elastase in Human Lung Epithelial Cells

Baginski L. 1 2
Buckley S. T. 1
Bakowsky U. 2
Ehrhardt C. 1
1 School of Pharmacy and Pharmaceutical Sciences, Trinity College Dublin, Ireland
2 Department of Pharmaceutical Technology and Biopharmaceutics, Philipps-Universität Marburg, Germany
E-mail: baginske@tcd.ie (L. Baginski), ehrhardc@tcd.ie (C. Ehrhardt)
Electronic publication date: 2010 Sep 30
Print publication date: 2010 Jul-Sep
Volume: 78
Issue: 3
First page: 720
Last page: 720
Copyright: © author(s); licensee Österreichische Apotheker-Verlagsgesellschaft m. b. H., Vienna, Austria.
Copyright year: 2010
License: This is an Open Access article distributed under the terms of the Creative Commons Attribution License (http://creativecommons.org/licenses/by/3.0/), which permits unrestricted use, distribution, and reproduction in any medium, provided the original work is properly cited.

JOURNAL INFORMATION
==============================
NLM Title Abbreviation: Sci Pharm
Journal ID: Sci Pharm
NLM Journal ID: 0026251
Journal ID: Scientia Pharmaceutica

Scientia Pharmaceutica

ISSN: 0036-8709
EISSN: 2218-0532
Publisher: Österreichische Apotheker-Verlagsgesellschaft, m. b. H.

ARTICLE INFORMATION
==============================
DOI: 10.3797/scipharm.cespt.8.POT06
Article ID: scipharm.2010.78.721
Subjects: Conference abstract POT06

Modern Dressings for Wound Healing

Rošic R.
Kristl J.
Baumgartner S.
Faculty of pharmacy, University of Ljubljana, Ljubljana, Slovenia
E-mail: romana.rosic@ffa.uni-lj.si (R. Rošic)
Electronic publication date: 2010 Sep 30
Print publication date: 2010 Jul-Sep
Volume: 78
Issue: 3
First page: 721
Last page: 721
Copyright: © author(s); licensee Österreichische Apotheker-Verlagsgesellschaft m. b. H., Vienna, Austria.
Copyright year: 2010
License: This is an Open Access article distributed under the terms of the Creative Commons Attribution License (http://creativecommons.org/licenses/by/3.0/), which permits unrestricted use, distribution, and reproduction in any medium, provided the original work is properly cited.

JOURNAL INFORMATION
==============================
NLM Title Abbreviation: Sci Pharm
Journal ID: Sci Pharm
NLM Journal ID: 0026251
Journal ID: Scientia Pharmaceutica

Scientia Pharmaceutica

ISSN: 0036-8709
EISSN: 2218-0532
Publisher: Österreichische Apotheker-Verlagsgesellschaft, m. b. H.

ARTICLE INFORMATION
==============================
DOI: 10.3797/scipharm.cespt.8.POT07
Article ID: scipharm.2010.78.722
Subjects: Conference abstract POT07

Defining Carbohydrate Targets on Osteoarthritic Chondrocytes using RT-qPCR and LC-ESI-MS

Wu S.Q. 1
Toegel S. 1
Pabst M. 2
Grass J. 2
Chiari C. 3
Unger F. M. 1
Altmann F. 2
Viernstein H. 1
1 Department of Pharmaceutical Technology and Biopharmaceutics, University of Vienna, Vienna, Austria
2 Department of Chemistry, University of Natural Resources and Applied Life Sciences, Vienna, Austria
3 Department of Orthopedics, Medical University Vienna, Vienna, Austria
E-mail: stefan.toegel@univie.ac.at (S. Toegel)
Electronic publication date: 2010 Sep 30
Print publication date: 2010 Jul-Sep
Volume: 78
Issue: 3
First page: 722
Last page: 722
Copyright: © author(s); licensee Österreichische Apotheker-Verlagsgesellschaft m. b. H., Vienna, Austria.
Copyright year: 2010
License: This is an Open Access article distributed under the terms of the Creative Commons Attribution License (http://creativecommons.org/licenses/by/3.0/), which permits unrestricted use, distribution, and reproduction in any medium, provided the original work is properly cited.

JOURNAL INFORMATION
==============================
NLM Title Abbreviation: Sci Pharm
Journal ID: Sci Pharm
NLM Journal ID: 0026251
Journal ID: Scientia Pharmaceutica

Scientia Pharmaceutica

ISSN: 0036-8709
EISSN: 2218-0532
Publisher: Österreichische Apotheker-Verlagsgesellschaft, m. b. H.

ARTICLE INFORMATION
==============================
DOI: 10.3797/scipharm.cespt.8.POT08
Article ID: scipharm.2010.78.723
Subjects: Conference abstract POT08

Interactions Of Different Sartans with the Bilayer Interface Studied by Saxs

Rappolt M. 1
Sartori B. 1
Hodzic A. 1 2
Pabst G. 1
Mavromoustakos T. 3
1 Institute of Biophysics and Nanosystems Research, Austrian Academy of Sciences, 8042 Graz, Austria
2 Research Center Pharmaceutical Engineering GmbH, 8010 Graz, Austria
3 Chemistry Department, National and Kapodistrian University of Athens, Panepistimioupolis Zographou 15771, Greece
E-mail: michael.rappolt@elettra.trieste.it (M. Rappolt)
Electronic publication date: 2010 Sep 30
Print publication date: 2010 Jul-Sep
Volume: 78
Issue: 3
First page: 723
Last page: 723
Copyright: © author(s); licensee Österreichische Apotheker-Verlagsgesellschaft m. b. H., Vienna, Austria.
Copyright year: 2010
License: This is an Open Access article distributed under the terms of the Creative Commons Attribution License (http://creativecommons.org/licenses/by/3.0/), which permits unrestricted use, distribution, and reproduction in any medium, provided the original work is properly cited.

JOURNAL INFORMATION
==============================
NLM Title Abbreviation: Sci Pharm
Journal ID: Sci Pharm
NLM Journal ID: 0026251
Journal ID: Scientia Pharmaceutica

Scientia Pharmaceutica

ISSN: 0036-8709
EISSN: 2218-0532
Publisher: Österreichische Apotheker-Verlagsgesellschaft, m. b. H.

ARTICLE INFORMATION
==============================
DOI: 10.3797/scipharm.cespt.8.POT09
Article ID: scipharm.2010.78.724
Subjects: Conference abstract POT09

Density Functional Theory Calculations on Methylated β-Cyclodextrins

Viernstein H. 1
Karpfen A. 2
Liedl E. 2
Snor W. 2
Wolschann P. 2
1 Department of Pharmaceutical Technology and Biopharmaceutics, University of Vienna, 1090 Vienna, Austria
2 Institute of Theoretical Chemistry, University of Vienna, 1090 Vienna, Austria
E-mail: helmut.viernstein@univie.ac.at (H. Viernstein)
Electronic publication date: 2010 Sep 30
Print publication date: 2010 Jul-Sep
Volume: 78
Issue: 3
First page: 724
Last page: 724
Copyright: © author(s); licensee Österreichische Apotheker-Verlagsgesellschaft m. b. H., Vienna, Austria.
Copyright year: 2010
License: This is an Open Access article distributed under the terms of the Creative Commons Attribution License (http://creativecommons.org/licenses/by/3.0/), which permits unrestricted use, distribution, and reproduction in any medium, provided the original work is properly cited.

JOURNAL INFORMATION
==============================
NLM Title Abbreviation: Sci Pharm
Journal ID: Sci Pharm
NLM Journal ID: 0026251
Journal ID: Scientia Pharmaceutica

Scientia Pharmaceutica

ISSN: 0036-8709
EISSN: 2218-0532
Publisher: Österreichische Apotheker-Verlagsgesellschaft, m. b. H.

ARTICLE INFORMATION
==============================
DOI: 10.3797/scipharm.cespt.8.POT10
Article ID: scipharm.2010.78.725
Subjects: Conference abstract POT10

Impact of Heat Stress on the Cellular Activity and Culturability of Probiotic Microorganisms

Salar-behzadi S. 1
Toegel S. 1
Hofrichter M. 1
Altenburger I. 1
Stummer S. 1
Basholli M. 1 2
Wirth M. 1
Unger F. M. 1
Viernstein H. 1
1 Department of Pharmaceutical Technology and Biopharmaceutics, University of Vienna, Vienna, Austria
2 Department of Pharmacy, University of Prishtina, QKUK, Prishtina, Republic of Kosova
E-mail: sharareh.salar-behzadi@univie.ac.at (S. Salar-Behzadi)
Electronic publication date: 2010 Sep 30
Print publication date: 2010 Jul-Sep
Volume: 78
Issue: 3
First page: 725
Last page: 725
Copyright: © author(s); licensee Österreichische Apotheker-Verlagsgesellschaft m. b. H., Vienna, Austria.
Copyright year: 2010
License: This is an Open Access article distributed under the terms of the Creative Commons Attribution License (http://creativecommons.org/licenses/by/3.0/), which permits unrestricted use, distribution, and reproduction in any medium, provided the original work is properly cited.

JOURNAL INFORMATION
==============================
NLM Title Abbreviation: Sci Pharm
Journal ID: Sci Pharm
NLM Journal ID: 0026251
Journal ID: Scientia Pharmaceutica

Scientia Pharmaceutica

ISSN: 0036-8709
EISSN: 2218-0532
Publisher: Österreichische Apotheker-Verlagsgesellschaft, m. b. H.

ARTICLE INFORMATION
==============================
DOI: 10.3797/scipharm.cespt.8.POT11
Article ID: scipharm.2010.78.726
Subjects: Conference abstract POT11

P-gp Inhibitory Potential of Phospholipids

Simon S.
Schubert R.
Department of Pharmaceutical Technology and Biopharmacy, University of Freiburg, Germany
E-mail: silke.simon@pharmazie.uni-freiburg.de (S. Simon)
Electronic publication date: 2010 Sep 30
Print publication date: 2010 Jul-Sep
Volume: 78
Issue: 3
First page: 726
Last page: 726
Copyright: © author(s); licensee Österreichische Apotheker-Verlagsgesellschaft m. b. H., Vienna, Austria.
Copyright year: 2010
License: This is an Open Access article distributed under the terms of the Creative Commons Attribution License (http://creativecommons.org/licenses/by/3.0/), which permits unrestricted use, distribution, and reproduction in any medium, provided the original work is properly cited.

JOURNAL INFORMATION
==============================
NLM Title Abbreviation: Sci Pharm
Journal ID: Sci Pharm
NLM Journal ID: 0026251
Journal ID: Scientia Pharmaceutica

Scientia Pharmaceutica

ISSN: 0036-8709
EISSN: 2218-0532
Publisher: Österreichische Apotheker-Verlagsgesellschaft, m. b. H.

ARTICLE INFORMATION
==============================
DOI: 10.3797/scipharm.cespt.8.POT12
Article ID: scipharm.2010.78.727
Subjects: Conference abstract POT12

Compartmental Modelling of the Pharmacokinetics of an Efflux Transporter

Grandjean T. R. B. 1
Yates J. T. W. 2
Chappell M. J. 1
1 School of Engineering, Warwick University, Coventry, United Kingdom
2 AstraZeneca R&D, Alderley Park, Cheshire, United Kingdom
E-mail: M.J.Chappell@warwick.ac.uk (M. J. Chappell)
Electronic publication date: 2010 Sep 30
Print publication date: 2010 Jul-Sep
Volume: 78
Issue: 3
First page: 727
Last page: 727
Copyright: © author(s); licensee Österreichische Apotheker-Verlagsgesellschaft m. b. H., Vienna, Austria.
Copyright year: 2010
License: This is an Open Access article distributed under the terms of the Creative Commons Attribution License (http://creativecommons.org/licenses/by/3.0/), which permits unrestricted use, distribution, and reproduction in any medium, provided the original work is properly cited.

This work was supported by a J3-9448 grant to T.L.R. and a young researcher grant to P.B., both from the Slovenian Research Agency.

This work was supported by FFG, Land Steiermark and Steirische Wirtschaftsförderung (SFG)

This work was supported by the Grant VEGA No. 1/ 0320/ 08

This work was supported by the De Reuter Fondation, Geneva, Switzerland.

This work was supported by the Grant VEGA No. 1/ 0320/ 08

This work was supported by the Grant VEGA No. 1/ 0320/ 08

This work was supported by the Austrian Science Fund (Project No. 19410)

This work was supported by Evonik Industries AG.

This work was supported by “Stiftung Aktion Österreich-Ungarn“.

This work was supported by scientifically assistance and knowledge share of Gordana Matijašević and Ivan Simčić in performing analytical tests.

This work was supported by the UK Engineering and Physical Sciences Research Council (CASE grant number 0900052) and by GlaxoSmithKline Ltd.

This work was supported by Serbian Ministry of Science and Technological Development, project number TR 20137.

This work was supported by Grant 006-0061117-1244 of the Ministry of Science, Education and Sports of the Republic of Croatia.

This work was supported by MedicActiv Vertriebs-GmbH (www.medicactiv.de).

The project is supported by one-A engineering austria, Prager Elektronik and the Austrian NANO Initiative (Nano-HEALTH).

This work was supported by Grant 006-0061117-1244 of the Ministry of Science, Education and Sports of the Republic of Croatia

This work was supported by FFG, Land Steiermark and Steirische Wirktschaftsförderung (SFG).

This work was supported by AZ & TG was supported by AZ and the MRC Capacity Building initiative.

This work was supported by the RCPE K1 Competence Center – Initiated by the Federal Ministry of Transport, Innovation & Technology (MMVIT) and the Federal Ministry of Economics & Labour (BMWA). Funded by FFG, Land Steiermark and Steirische Wirtschaftsförderung (SFG).

This work was done under the project Biopharmaceutical Characterization of the selected BCS Class II and III drugs: In Vitro and In Silico Methods Evaluation (TR-23015) supported by the Ministry of Science and Technological Development, Republic of Serbia.

This work is supported by the 7th Research Framework Programme of the European Commission (Grant agreement number: NMP2-SL-2008-206707).

This work was supported by the FFG, SFG, Das Land Steiermark, Ortner Clean Rooms Unlimited and Dastex.

This work was supported by TÁMOP-Hungary research project: Development of teranostics in cardiovascular, metabolic, and inflammatory diseases (TÁMOP-4.2.2-08/1-2008-0013).

MIA is funded by the University of Warwick Vice Chancellor’s PhD Scholarship.

This work was funded by a Strategic Research Cluster grant (07/SRC/B1154) under the National Development Plan co-funded by EU Structural Funds and Science Foundation Ireland and by an IRCSET Embark Postgraduate Scholarship.

This work was financially supported by Phospholipid e.V., Heidelberg, Germany

Work supported by the UK MRC Capacity Building Intitiative & AstraZeneca.

